# Efficacy and safety of 11 oral preparations of single-source traditional Chinese medicines in the treatment of unstable angina pectoris: a systematic review and network meta-analysis

**DOI:** 10.3389/fphar.2025.1582661

**Published:** 2025-06-24

**Authors:** Xiuchong Li, Xiaohui Li, Xing Zhu, Xin Xin, Ran Sheng, Te Wang, Junming Kan, Yongsheng Huang

**Affiliations:** ^1^ College of Traditional Chinese Medicine, Changchun University of Chinese Medicine, Changchun, Jilin, China; ^2^ Heart Center, The First Affiliated Hospital of Henan University of Chinese Medicine, Zhengzhou, Henan, China; ^3^ Department of Cardiovascular, The Affiliated Hospital of Changchun University of Chinese Medicine, Changchun, Jilin, China; ^4^ Department of Cardiovascular, The Third Affiliated Hospital of Changchun University of Chinese Medicine, Changchun, Jilin, China

**Keywords:** network meta-analysis, traditional Chinese medicine, unstable angina pectoris, oral preparation, ethnopharmacology research

## Abstract

**Background:**

Unstable angina pectoris (UAP) is a cardiovascular disease with high morbidity and can cause serious cardiovascular complications. Oral preparations of single-source traditional Chinese medicines (SSTCM-OPs) are increasingly used as adjuncts to conventional treatments (CT) for UAP, providing complementary therapeutic advantages with favorable safety profiles. However, the comparative efficacy and safety of these SSTCM-OPs remain unclear. This network meta-analysis (NMA) evaluates the efficacy and safety of 11 approved SSTCM-OPs to guide clinical practice in UAP treatment.

**Methods:**

A comprehensive literature search was conducted across eight databases: China National Knowledge Infrastructure (CNKI), China Science and Technology Journal Database (VIP), Wanfang Database, China Biomedical Literature Database (CBM), Web of Science, PubMed, EMBASE, and the Cochrane Library. Only randomized controlled trials (RCTs) examining SSTCM-OPs combined with CT for UAP were included. The search covered publications up to 4 December 2024. Quality assessment was performed using RevMan 5.4.1, and certainty of evidence was evaluated with GRADEpro software 3.6.1. A frequentist random-effects model was employed for NMA. Statistical analysis was performed using Stata 18.0.

**Results:**

A total of 72 RCTs involving 11 SSTCM-OPs and 7,360 patients were included. The NMA results demonstrated that Maixuekang oral preparation combined with CT and Xinyue oral preparation combined with CT showed superiority in terms of angina efficacy; Xindakang oral preparation combined with CT and Yinxingtongzhi oral preparation combined with CT showed superiority in terms of Electrocardiogram (ECG) efficacy and had an advantage in reducing nitroglycerin dosages; Xindakang oral preparation combined with CT and Yinxingtongzhi oral preparation combined with CT showed superiority in reducing nitroglycerin dosages; Zhenyuan oral preparation combined with CT and Diaoxinxuekang oral preparation combined with CT showed superiority in reducing frequency of angina; Xinnaoshutong oral preparation combined with CT and Yinxingtongzhi oral preparation combined with CT showed superiority in reducing duration of angina; Xuezhikang oral preparation combined with CT showed superiority in improving TC, LDL-C, and HDL-C levels as well as reducing the occurrence of MACEs; Yinxingtongzhi oral preparation combined with CT showed superiority in improving TG and PV levels; Lastly, Xuesaitong oral preparation combined with CT and Yinxingye oral preparation showed superiority in reducing hs-CRP levels.

**Conclusion:**

All 11 SSTCM-OPs combined with CT showed advantages over CT alone in treating UAP. Notably, Xinnaoshutong + CT did not significantly reduce angina frequency, but it was effective in other outcomes. These findings suggest incorporating traditional Chinese medicine into standardized treatment regimens may enhance UAP management.

**Systematic Review Registration:**

https://www.crd.york.ac.uk/PROSPERO/, identifier CRD42024618094.

## 1 Introduction

Unstable angina pectoris (UAP) is classically defined as a common acute coronary syndrome characterized by myocardial ischemia in the absence of myocardial necrosis (Braunwald and Morrow, 2013; Reichlin et al., 2013; Zaacks et al., 1999). The China Cardiovascular Health and Disease Report 2023 reveals that the number of individuals diagnosed with coronary heart disease (CHD) in China has escalated to 11.39 million, while the annual admissions of adult patients hospitalized for CHD have surged to 6.127 million. Among these cases, unstable angina pectoris constitutes the predominant discharge diagnosis, representing 38.1% of the total (National Center for Cardiovascular Disease, 2024). Chronic angina pectoris frequently leads to severe cardiovascular complications, including myocardial infarction and heart failure, thereby markedly elevating the risk to patient health and safety.

Notably, the pathophysiological mechanisms of unstable angina pectoris and acute coronary syndrome (ACS, including myocardial infarction) have traditionally been attributed to the rupture of a single vulnerable plaque, characterized by a thin fibrous cap, large lipid core, and substantial inflammatory cell infiltration (e.g., macrophages and lymphocytes) (Hansson et al., 2015; Libby, 2001). This rupture leads to local thrombus formation, resulting in myocardial ischemia and acute symptoms (Libby, 2001). However, studies have shown that in unstable angina patients, neutrophils and other inflammatory cells are activated throughout the coronary artery system, not just at the site of the “culprit lesion” (Buffon et al., 2002; Libby et al., 2018). Thus, the pathogenesis of ACS is now understood as a combination of local plaque rupture and a systemic inflammatory response.

The currently recognized pharmacological treatments for UAP primarily include nitrates, β-blockers, statins, cyclooxygenase inhibitors, P2Y12 receptor antagonists, and anticoagulants (Amsterdam et al., 2014; Chinese Society of Cardiology and Editorial Board of Chinese Journal of Cardiology, 2024; Collet et al., 2021). Given the diverse mechanisms of action and side effects of antianginal medications, their use should be tailored to the patient’s medical history and potential drug interactions. Nevertheless, individualized pharmacotherapy continues to pose considerable challenges, due to the limited availability of reliable data from large randomized controlled trials (RCTs) (Manfredi et al., 2022). Moreover, current evidence reveals that no single class of drugs has been found to simultaneously improve quality of life and prevent cardiovascular events, which presents another major challenge (Ferrari et al., 2018).

Traditional Chinese medicine (TCM) plays a significant role in complementary and alternative medicine (CAM), with its holistic regulatory approach and emphasis on personalized treatment aligning closely with the core principles of CAM (Ding et al., 2024; Wang and Zhang, 2017). With the advancement of modern biotechnology, an increasing number of traditional Chinese medicines with remarkable bioactive properties have been extracted and standardized, then processed into preparations under strict quality control, ultimately applied to disease treatment (Fu et al., 2024; Sun et al., 2015). At present, traditional Chinese medicine preparations are divided into compound preparations and single-source preparations according to the *Pharmacopoeia of the People’s Republic of China* (National Pharmacopoeia Commission, 2020). In this context, oral preparations of single-source traditional Chinese medicines (SSTCM-OPs) are emerging as a promising complementary approach for unstable angina pectoris, which offer additional benefits such as anti-inflammatory, antioxidant effects, inhibition of platelet aggregation, and vasodilatory effects (Tao et al., 2022; Zhang W. et al., 2020; Zhao et al., 2025). These properties are particularly valuable in addressing some of the gaps left by conventional treatments (CT). Thus, we manually searched the 2024 edition of China’s National Catalogue of Medicines for Basic Medical Insurance, Work Injury Insurance and Maternity Insurance, and identified the following 11 SSTCM-OPs approved for the treatment of UAP: Zhenyuan oral preparation (ZY), Xuesaitong oral preparation (XST), Xinyue oral preparation (XY), Diaoxinxuekang oral preparation (DAXXK), Xuezhikang oral preparation (XZK), Yinxingye oral preparation (YXY), Yinxingtongzhi oral preparation (YXTZ), Xindakang oral preparation (XDK), Xinnaoshutong oral preparation (XNST), Dazhuhongjingtian oral preparation (DZHJT) and Maixuekang oral preparation (MXK).

Several meta-analyses (Liu et al., 2024a; Man et al., 2020; Song et al., 2017; Yang et al., 2013) have shown that single-source TCM preparations, including XST, DZHJT, YXY, and YXTZ, when used in combination with conventional treatments, reduce symptoms, improve patient outcomes, and maintain a favorable safety profile for UAP. Although some of these studies included both oral and injectable forms, our analysis focuses specifically on the oral preparations. Additionally, oral preparations such as XZK and MXK have shown efficacy in improving lipid levels in UAP patients, thereby providing safer, more effective cardiovascular treatment options (Cui et al., 2015; Zhou, 2019). Despite these advantages, the lack of head-to-head trials for these preparations makes it difficult to determine which specific drugs may offer the greatest therapeutic advantage as adjunctive therapies, leading to uncertainty among clinicians when selecting treatments in clinical practice. Network meta-analysis (NMA) integrates both direct and indirect evidence, allowing for the ranking of the effectiveness of multiple interventions (Nino and Brignardello-Petersen, 2023). Therefore, we employed NMA to assess the efficacy of various interventions based on RCTs and report their safety profiles, aiming to assist clinicians in identifying the optimal treatment options.

## 2 Materials and methods

### 2.1 Registration and reporting

This systematic review and network meta-analysis were registered in the international prospective systematic review registration database PROSPERO (ID: CRD42024618094). This study strictly followed the guidelines outlined in the Preferred Reporting Items for Systematic Reviews and Meta-Analyses (PRISMA) and the PRISMA-extension statement for NMA (Hutton et al., 2015; Shamseer et al., 2015).

### 2.2 Standard evaluation of traditional Chinese medicine

In order to address terminological ambiguity and avoid misuse, we standardized the scientific names of the included Chinese medicines by referring to the relevant recommendations (Rivera et al., 2013; Wu et al., 2022), and the following three websites: “The World Flora Online” (WFO, http://www.worldfloraonline.org/), “China Animal Scientific Database” (CASD, http://www.zoology.csdb.cn/) and “Species Diversity Data Platform” (SDDP, http://www.especies.cn/). The main details of the Chinese medicine oral preparations are shown in Table 1. Supplementary Material S1 provides detailed information on the chemical metabolites of oral preparations of single-source traditional Chinese medicines.

**TABLE 1:** Main details of the Chinese medicine oral preparations.

Drug name	Latin name	Family	Drug part used	Main bioactive metabolites
Zhenyuan oral preparation	*Panax ginseng C.A.Mey.*	Araliaceae	Fruit	Notoginsenoside (R1, *etc.*) and ginsenoside (Rg1, Rb1, Re, Rf, *etc.*)
Xuesaitong oral preparation	*Panax notoginseng (Burkill) F.H.Chen*	Araliaceae	Root and rhizome	Notoginsenoside (R1, *etc.*) and Ginsenoside (Rg1, Rb1, Re, Rf, Rd, *etc.*)
Xinyue oral preparation	*Panax quinquefolius L.*	Araliaceae	Stem and foliage	Ginsenoside (Rg1, Rb1, Re, Rd, *etc.*) and Pseudoginsenoside F11
Diaoxinxuekang oral preparation	*Dioscorea panthaica Prain & Burkill or Dioscorea nipponica Makino*	Dioscoreaceae	Stem and foliage	Protodioscin, Dioscin and Pseudoprodioscin
Xuezhikang oral preparation	*Monascus purpureus Went.*	—	—	Lovastatin hydroxy acid, Mevastatin and Lovastatin, *etc.*
Yinxingye oral preparation	*Ginkgo biloba L.*	Ginkgoaceae	Foliage	Ginkgolide (A, B, C, J(M)), bilobalide, rutin, quercetin, kaempferitin, and isorhamnetin, *etc.*
Yinxingtongzhi oral preparation	*Ginkgo biloba L.*	Ginkgoaceae	Foliage	Ginkgolide (A, B, C, J(M)), bilobalide, rutin, quercetin, kaempferitin, and isorhamnetin, *etc.*
Xindakang oral preparation	*Hippophae rhamnoides L.*	Elaeagnaceae	Fruit	Quercetin, kaempferitin and isorhamnetin
Xinnaoshutong oral preparation	*Tribulus terrestris L.*	Zygophyllaceae	Grass	Terrestrinin, rutin, quercetin, kaempferitin, and isorhamnetin, *etc.*
Dazhuhongjingtian oral preparation	*Rhodiola crenulata (Hook.f. and Thomson) H.Ohba*	Crassulaceae	Root and rhizome	Gallic acid, 5-hydroxymethylfurfural, rhodiocyanoside A, 3,4-dihydroxybenzoic acid, spruce glycoside and rhodioloside, *etc.*
Maixuekang oral preparation	*Whitmania pigra Whitman or Whitmania acranutata Whitman or Hirudo nipponica Whitman*	Hirudinidae	Dry whole	Hirudin and thrombin-inhibiting peptide, *etc.*

### 2.3 Search strategy

**FIGURE 1:**
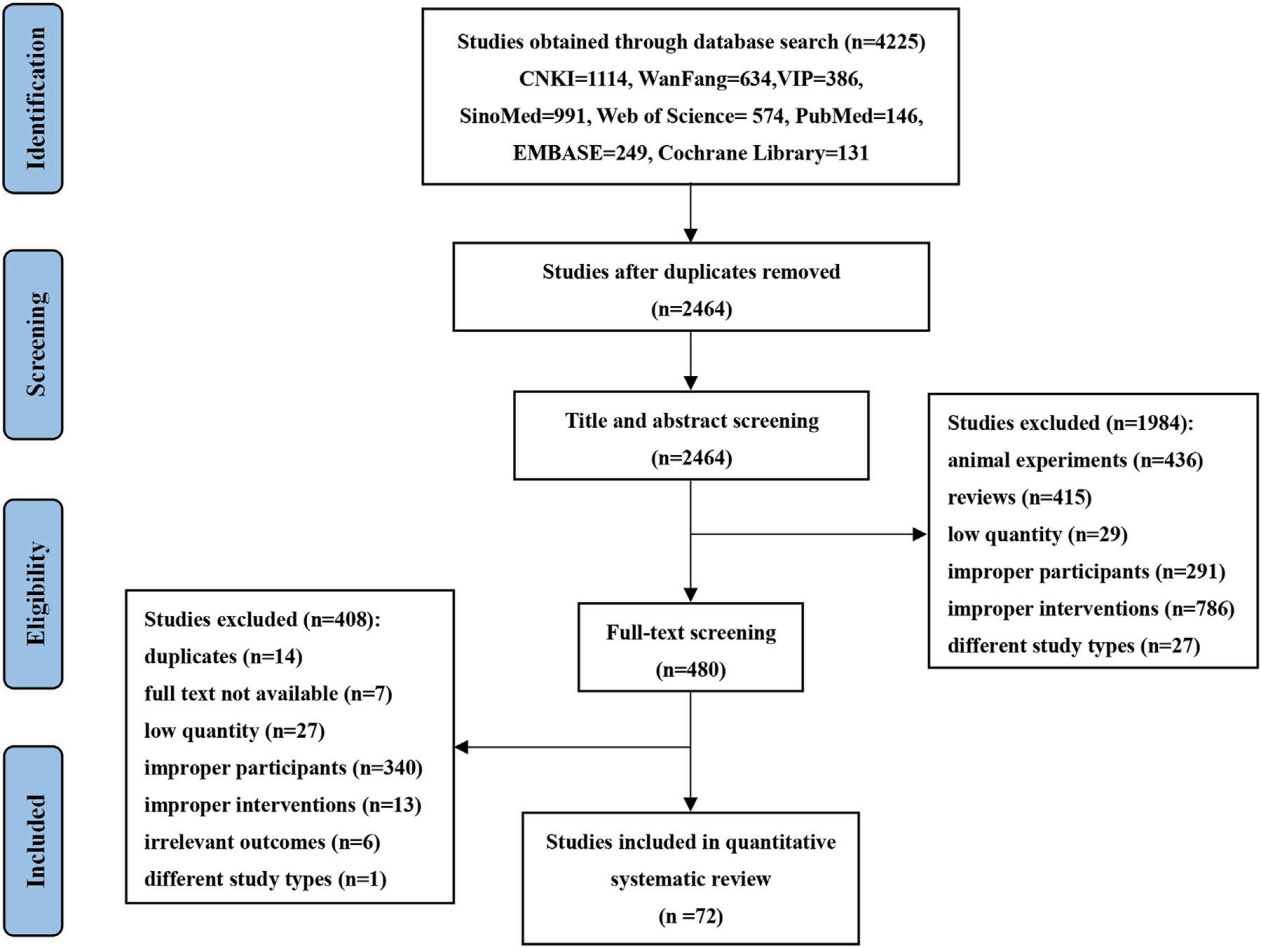
PRISMA flowchart diagram of the search process for studies.

Eight databases were searched: China National Knowledge Infrastructure (CNKI), China Science and Technology Journal Database (VIP), Wanfang Database, Chinese Biomedical Literature Database (CBM), Web of Science, PubMed, EMBASE, and Cochrane Library from the database inception of each database up to 4 December 2024. Search strategies integrated subject terms and free terms. The English search strategy included the following key components: “Zhenyuan,” “Xuesaitong,” “Xinyue,” “Diaoxinxuekang,” “Xuezhikang,” “Yinxingye,” “Ginkgo ketone ester,” “Xindakang,” “Xinnaoshutong,” “Dazhuhongjingtian,” “Maixuekang” “Coronary Disease,” “Acute Coronary Syndrome,” “Angina Unstable,” “Myocardial Ischemia,” “Angina Pectoris” and “Randomized Controlled.” The detailed screening process is illustrated in Figure 1, specific search strategies are provided in Supplementary Material S2.

### 2.4 Eligibility criteria

The eligibility criteria, using PICOS (Population, Intervention, Comparators, Outcomes, and Study design) as the main framework, are as follows.

#### 2.4.1 Study design

Only randomized controlled trials (RCTs) were included, with no restrictions on blinding method or language.

#### 2.4.2 Population

Patients must meet the UAP diagnostic criteria recognized at the time of study publication, or alternatively, their diagnostic typing of UAP was confirmed. Patients in any of the following conditions were excluded: acute myocardial infarction, severe heart failure, bleeding tendency, severe liver or kidney disease, or malignancy, with a life expectancy of less than 1 year, and no restrictions on gender, race, region, or age.

#### 2.4.3 Interventions/comparators

The control groups were receiving exclusively conventional treatments (CT) of UAP, which mainly consisted of nitrates, β-blockers, calcium antagonists, morphine sulfate, nicorandil, trimetazidine, aspirin, clopidogrel, heparin, and statins. Whereas the treatment group was treated with the 11 most commonly used SSTCM-OPs combined with CT, or the comparison between any two SSTCM-OPs on the basis of conventional treatments. Studies that did not meet the above inclusion criteria were excluded. SSTCM-OPs covered all oral dosage forms, including capsule, tablet, dispersion, pill, cream, elixir, drop, granule, oral liquid, and all injectable interventions were excluded. There is no restriction on the dosages and manufacturers. Different dosage forms of the same SSTCM-OP were grouped for NMA. For instance, Zhenyuan oral preparation includes Zhenyuan tablet, Zhenyuan capsule, and Zhenyuan oral liquid; we use Zhenyuan (ZY) as a unified combination for NMA to find the optimal therapeutic solution.

#### 2.4.4 Outcome measures

Angina efficacy and Electrocardiogram (ECG) efficacy were classified into three outcomes: “significantly effective,” “effective,” and “ineffective,” based on the criteria established by China’s official medical organization (Department of Internal Medicine Society of Chinese Medicine Society, 2008; Liu et al., 1998). For angina efficacy, “significantly effective” was defined as an over 80% reduction in angina frequency after treatment; “effective” was defined as an over 50% reduction in angina frequency but not exceeding 80% after treatment; “ineffective” was defined as a reduction in angina frequency of less than 50%, or no change in frequency after treatment. For ECG efficacy, “significantly effective” was defined as a recovery of the ST segment on the resting ECG by more than 0.1 mV, or a return to normal ST segment levels; “effective” was defined as a reduction in resting ECG ST segment depression of less than 0.05 mV, an improvement of more than 0.05 mV, or a T wave inversion becoming shallower by more than 50% or changing from flat to upright; “ineffective” was defined as an improvement of the ST segment by less than 0.05 mV, or no change in the T wave (Zheng, 2002). The two aforementioned indicators were employed as the primary endpoints for evaluation. Meanwhile, duration of angina, frequency of angina, and nitroglycerin dosages are commonly used indicators to evaluate the condition of angina pectoris. As a marker of the inflammatory state of vulnerability of atherosclerotic plaque and coronary artery disease activity, high-sensitivity C-reactive Protein (hs-CRP) is of great significance in predicting future cardiovascular events (Arroyo-Espliguero et al., 2004; Schillinger et al., 2003). Plasma viscosity (PV) is also a risk factor for predicting future cardiovascular events, and can be used to evaluate blood flow and assess the body’s circulatory status (Tzoulaki et al., 2007). Meanwhile, maintaining optimal lipid levels can slow down the progression of atherosclerosis, which is of great significance for preventing cardiovascular events (Ference et al., 2018). Therefore, the above indicators are also evaluated as secondary outcomes.

In summary, the Primary outcome: 1) Angina efficacy (Significantly effective + Effective), 2) ECG efficacy (Significantly effective + Effective). Secondary outcome: 1) Nitroglycerin dosages, 2) Frequency of angina, 3) Duration of angina (min/times), 4) Total cholesterol (TC), 5) Triglyceride (TG), 6) Low-density lipoprotein cholesterol (LDL-C), 7) High-density Lipoprotein Cholesterol (HDL-C), 8) hs-CRP, 9) PV, 10) Major adverse cardiovascular events (MACEs).

In addition, the exclusion criteria were applied: 1) Duplicate publications; 2) Use of other TCM treatment measures (e.g., other commercial Chinese polyherbal preparations, acupuncture, or other traditional therapies) in both groups; 3) Non-clinical studies (animal experiments), reviews, low-quality studies, inappropriate participant selection, non-compliant interventions, irrelevant outcomes, unavailable full text, or non-RCT study designs; 4) Incorrect or incomplete data in the literature.

### 2.5 Study selection and data extraction

Two researchers (XcL and XZ) independently screened the literature and extracted the data. After removing duplicate studies, the titles and abstracts of the remaining articles were reviewed for initial screening. Full texts were then read and re-screened according to the predefined inclusion and exclusion criteria to determine the final eligible studies. Data extraction, including baseline characteristics, interventions, and outcomes, was performed using Microsoft Excel 2019. Any discrepancies were resolved through discussion to reach consensus or by consulting a third-party reviewer (TW). In cases where standard deviations were not explicitly reported, alternative statistical parameters, including standard errors, confidence intervals, or relevant test statistics (t/p values), were utilized to calculate standard deviation. Following the Cochrane Handbook recommendations, multiple attempts (at least three email contacts) were made to obtain missing data directly from the corresponding authors. For graphical data without numerical values, extraction was performed using GetData Graph Digitizer 2.20, with results cross-verified by two independent researchers to ensure accuracy.

### 2.6 Quality assessment and certainty of evidence

Two reviewers (XhL and XX) evaluated the methodological quality of included RCTs using the risk-of-bias tool from the Cochrane Handbook for Systematic Reviews of Interventions (Higgins et al., 2011), as implemented in RevMan 5.4.1. The assessment indicators consisted of 7 domains: random sequence generation (selection bias); allocation concealment (selection bias); blinding of participants and personnel (performance bias); blinding of outcome assessment (detection bias); incomplete outcome data (attrition bias); selective reporting (reporting bias); other bias. The risk of bias was categorized as “high risk,” “low risk,” and “some concerns” for each item. Disagreements, if any, were resolved by a third-party reviewer (RS).

In this study, the certainty of evidence was systematically evaluated using the Grading of Recommendations, Assessment, Development, and Evaluation (GRADE) framework (Kavanagh, 2009). Following GRADE guidelines, potential downgrades in evidence certainty were assessed across five key domains: risk of bias, inconsistency, indirectness, imprecision, and publication bias. These evaluations were conducted using GRADEpro software 3.6.1 to standardize the process by two reviewers (XhL and RS). Final evidence ratings were rated as four levels: high, moderate, low, and very low.

### 2.7 Statistical analysis

Stata 18.0 software was used for statistical analyses and network plotting. Initially, we conducted a pairwise meta-analysis to assess the direct comparisons between various SSTCM-OPs and the control group. All results were expressed as 95% confidence intervals (CI). Heterogeneity was assessed using the *I* ^2^ statistic, with a threshold of 50% to determine the appropriate analytical model. Significant heterogeneity, evidenced by an *I* ^2^ value exceeding 50%, prompted the use of a random-effects model to account for between-study variability. When the *I* ^2^ statistic was 50% or lower, suggesting minimal or no substantial heterogeneity, a fixed-effects model was employed to provide more precise estimates.

This NMA was performed using a frequentist approach with a random-effects model, synthesizing both direct and indirect evidence from all eligible studies to compare the effectiveness of multiple interventions. Binary outcomes used relative risk (RR), continuous outcomes with consistent units used mean difference (MD), and continuous outcomes with differing units used standardized mean difference (SMD). In the frequentist framework of NMA, the transitivity assumption was evaluated by visually inspecting the network plot and statistically assessing the consistency of treatment effects across studies. The network plot illustrates the comparisons between interventions, where lines connecting nodes represent direct comparisons between two interventions, and indirect comparisons are inferred through the network structure. In the plot, the line width indicates the number of studies directly comparing interventions, while the node size reflects the total number of participants across studies. If the evidence network contained closed loops of evidence, inconsistency tests were conducted; otherwise, a consistency model was used. As no closed evidence loops existed among the interventions included in this NMA, only a consistency model was adopted.

Subsequently, consistency between direct and indirect evidence was evaluated using a global approach (treatment-by-interaction model) to test for overall inconsistency across the network, supplemented by a local approach (node-splitting method) to identify specific discrepancies between direct and indirect comparisons for each treatment contrast. The surface under the cumulative ranking curve (SUCRA) was calculated to rank the efficacy of each SSTCM-OP and determine the most effective intervention. SUCRA values range from 0 to 1, reflecting the relative likelihood of each intervention being ranked as the most effective, with higher values indicating better outcomes (Mbuagbaw et al., 2017).

Finally, we conducted a sensitivity analysis and subgroup analysis to mitigate confounding factors and explore sources of heterogeneity. Meanwhile, meta-regression analysis was specifically implemented for the XZK + CT regimen, with rigorous examination of the temporal relationship linking treatment course (weeks) to LDL-C reduction (mmol/L). This specific analytical focus was mandated by the singular availability of sufficient data (≥10 studies) meeting our pre-established criteria for reliable covariate-effect estimation. Non-informative priors were specified for regression coefficients (*β*) to ensure data-driven estimation, and covariate significance was determined *via* 95% confidence intervals excluding the null value and p-values (<0.05). Publication bias was assessed by drawing funnel plots to evaluate small-study effects, where asymmetry in the plot may indicate potential bias.

## 3 Results

### 3.1 Study selection and characteristics

Initially, 4,225 studies were found through search queries, and 2,464 studies remained after duplicate removal *via* EndNote 20. 480 studies were retained after excluding animal experiments, reviews, low-quality studies, improper participants, improper interventions, and different study types by screening titles and abstracts. After full-text review, we excluded studies for various reasons, including duplicates, unavailable full text, low-quality studies, improper participants, improper interventions, irrelevant outcomes, and different study types. As a result, 408 studies were excluded. Ultimately, a total of 72 clinical trials met the inclusion criteria. The detailed screening process is illustrated in Figure 1. Across 72 studies, 7,360 patients were involved, with 3,707 in the treatment group and 3,653 in the control group, examining 11 SSTCM-OPs. The number of included studies for each preparation is as follows: Zhenyuan oral preparation (2 RCTs) (Liu Q. et al., 2018; Wang, 2022), Xuesaitong oral preparation (12 RCTs) (Dong et al., 2020; Duan et al., 2022; Fan and Zhang, 2023; Feng et al., 2009; Ge and Zhao, 2010; Huang and Zhang, 2023; Kuang et al., 2011; Liu et al., 2008; Wang et al., 2017; Wei, 2010; Yu, 2010; Zhao, 2002), Xinyue oral preparation (3 RCTs) (Liu, 2015; Lu, 2022; Zhang R. et al., 2020), Diaoxinxuekang oral preparation (4 RCTs) (Xu and Fang, 2019; Xu, 2020; Xu, 2016; Yu et al., 2023), Xuezhikang oral preparation (14 RCTs) (Chen, 2015; Cui et al., 2014; Cui and Li, 2016; Dai et al., 2015; Dai et al., 1999; Lin et al., 2009; Liu et al., 2013; Ma and Deng, 2019; Ma et al., 2014; Ren and Peng, 2006; Su et al., 2017; Wang et al., 2006; Xiong et al., 2008; Zhu and Li, 2008), Yinxingye oral preparation (10 RCTs) (Chen, 2012; Du et al., 2018; Gao and Gao, 2013; He, 2014; Jia, 2014; Sun et al., 2024; Wang and Sun, 2012; Xia and Zhang, 2003; Yu and Yang, 2023; Zhang et al., 2011), Yinxingtongzhi oral preparation (10 RCTs) (Dong and Yu, 2015; Huang, 2016; Li et al., 2019; Liu, 2020; Liu Y. et al., 2018; Luo and Yu, 2013; Peng et al., 2020; Sheng et al., 2022; Song, 2021; Wu, 2014), Xindakang oral preparation (3 RCTs) (Huang, 2022; Huang, 2018; Li et al., 2003), Xinnaoshutong oral preparation (3 RCTs) (Ma et al., 2009; Wang, 2015; Zhang et al., 2012), Dazhuhongjingtian oral preparation (2 RCTs) (Liu and Zhu, 2019; Shen et al., 2014) and Maixuekang oral preparation (9 RCTs) (Chen and Chen, 2021; Chen et al., 2021; Gao, 2010; Hao, 2022; Li, 2014; Luo, 2013; Wang et al., 2012; Xing and Lv, 2015; Yue and Zhang, 2013). The basic characteristics of the included studies in this NMA are shown in Table 2.

**TABLE 2:** Characteristics of the included studies in this network meta-analysis.

Included studies	Sample (T/C)	Sex (M/F)		Age (years)		Manufacturer	Specification/dosage form	Intervention		Course (weeks)	Outcomes
		T	C	T	C			T	C		
Liu et al. (2018a)	40/40	23/17	25/15	67.38 ± 11.25	66.60 ± 14.56	Yisheng	0.25 g/capsule	ZY 2 capsules tid + CT	CT	4	①②⑩
Wang (2022)	41/41	24/17	23/18	58.90 ± 5.60	58.79 ± 6.45	Yisheng	0.25 g/capsule	ZY 2 capsules tid + CT	CT	4	①②④⑤⑩
Wang et al. (2017)	40/40	17/23	21/19	70.68 ± 6.87	71.65 ± 4.32	Shenghuo	0.33 g/soft capsule	XST 2 capsules bid + CT	CT	4	⑥⑦⑧⑨
Fan and Zhang (2023)	88/88	60/28	56/32	57.39 ± 9.12	58.19 ± 8.48	Kpc	0.10 g/soft capsule	XST 2 capsules tid + CT	CT	2	①⑩
Dong et al. (2020)	40/38	32/8	24/14	62.23 ± 8.40	61.89 ± 7.52	Shenghuo	0.33 g/soft capsule	XST 2 capsules bid + CT	CT	4	⑥⑦⑧
Huang and Zhang (2023)	34/34	16/18	17/17	52.5 ± 5.4	55.3 ± 7.9	Kpc	0.10 g/soft capsule	XST 2 capsules tid + CT	CT	4	①③④⑤
Yu (2010)	50/50	29/21	28/22	64.18 ± 12.13	62.8 ± 10.8	Shenghuo	0.33 g/soft capsule	XST 2 capsules bid + CT	CT	4	①②⑫
Ge and Zhao (2010)	48/48	22/26	25/23	56	54	Shenghuo	0.33 g/soft capsule	XST 2 capsules bid + CT	CT	4	①②
Wei (2010)	90/90	113/67		60.4 ± 3.5		Shenghuo	0.33 g/soft capsule	XST 2 capsules bid + CT	CT	4	①②④⑤
Feng et al. (2009)	32/30	16/16	17/13	68.79	69.12	Shenghuo	0.33 g/soft capsule	XST 2 capsules bid + CT	CT	4	①②⑪
Zhao (2002)	23/23	28/18		60.6 ± 11.3		NA	capsule	XST 2 capsules bid + CT	CT	NA	③⑫
Duan et al. (2022)	43/41	32/11	28/13	65.38 ± 8.68	63.63 ± 7.05	Shenghuo	0.33 g/soft capsule	XST 2 capsules bid + CT	CT	4	④⑤⑥⑦⑧⑨⑫
Liu et al. (2008)	30/30	NA		64.6 ± 5.4	63.6 ± 4.5	Weihe	0.06 g/capsule	XST 2 capsules bid + CT	CT	4	①⑥⑦⑧⑨
Kuang et al. (2011)	90/90	47/43	46/44	56.3 ± 6.9	57.1 ± 7.2	Shenghuo	0.33 g/soft capsule	XST 2 capsules bid + CT	CT	4	①④⑤
Zhang et al. (2020b)	45/45	32/13	31/14	57.8 ± 7.5	58.4 ± 6.9	Yisheng	0.30 g/capsule	XY 0.6 g tid + CT	CT	4	①③④⑤
Lu (2022)	39/39	25/14	27/12	55.69 ± 5.71	55.26 ± 5.63	Yisheng	0.30 g/capsule	XY 0.6 g tid + CT	CT	4	④⑤
Liu (2015)	48/48	58/38		63.6		Yisheng	0.30 g/capsule	XY 0.6 g tid + CT	CT	4	①
Xu (2020)	29/29	18/11	17/12	64.82 ± 5.66	65.77 ± 5.25	Diao	0.10 g/capsule	DAXXK 2 capsules tid + CT	CT	2	①⑪
Xu and Fang (2019)	60/60	40/20	41/19	66.48 ± 10.3	67.93 ± 12.07	Diao	0.10 g/capsule	DAXXK 2 capsules tid + CT	CT	1	①②④⑤
Xu (2016)	56/56	30/26	32/24	58.9 ± 8.2	59.6 ± 8.4	Diao	0.10 g/capsule	DAXXK 2 capsules tid + CT	CT	2	①④⑤⑪
Yu et al. (2023)	53/53	27/26	29/24	66.87 ± 11.81	65.89 ± 12.34	Diao	0.35 g/soft capsule	DAXXK 2 capsules tid + CT	CT	2	①③④⑤⑩
Dai et al. (2015)	41/41	27/14	25/16	64.2 ± 11.2	64.4 ± 11.4	WBL Biotech	0.30 g/capsule	XZK 2 capsules bid + CT	CT	12	①⑫
Liu et al. (2013)	42/40	25/17	24/16	63.4 ± 10.5	62.9 ± 10.3	WBL Biotech	0.30 g/capsule	XZK 2 capsules bid + CT	CT	48	⑦⑧⑩⑫
Wang et al. (2006)	30/30	34/26		60.5 ± 10.6		WBL Biotech	0.30 g/capsule	XZK 0.6 g bid + CT	CT	2	⑧⑨
Dai et al. (1999)	33/25	19/14	17/8	57 ± 9	56 ± 8	WBL Biotech	0.30 g/capsule	XZK 0.6 g bid + CT	CT	8	①②⑥⑦⑧⑨
Ren and Peng (2006)	38/30	28/10	26/4	62.0 ± 7.6	61.0 ± 7.2	WBL Biotech	capsule	XZK 2 g tid + CT	CT	4	①②
Chen (2015)	31/31	16/15	17/14	68.3 ± 6.9	68.7 ± 7.2	WBL Biotech	0.30 g/capsule	XZK 0.6 g qd + CT	CT	12	①⑥⑦⑧⑨⑫
Ma and Deng (2019)	76/74	78/72		55.0 ± 5.8		WBL Biotech	0.30 g/capsule	XZK 0.6 g bid + CT	CT	24	④⑤⑥⑦⑧⑨
Su et al. (2017)	80/80	42/38	45/35	57.8 ± 10.9	58.5 ± 10.4	WBL Biotech	0.40 g/tablet	XZK 0.8 g bid + CT	CT	12	①⑥⑦⑧⑨
Lin et al. (2009)	24/24	14/10	13/11	55.4		WBL Biotech	0.30 g/capsule	XZK 0.6 g bid + CT	CT	24	①⑥⑧⑫
Cui et al. (2014)	32/32	NA		48–82		WBL Biotech	capsule	XZK 0.6 g bid + CT	CT	4	⑥⑦⑧⑨⑩
Cui and Li (2016)	58/58	30/28	29/29	63.15 ± 9.18	62.85 ± 9.26	WBL Biotech	capsule	XZK 2 capsules bid + CT	CT	48	⑥⑦⑧⑩
Xiong et al. (2008)	62/40	43/19	27/13	56.1 ± 8.2	55.2 ± 8.4	WBL Biotech	capsule	XZK 0.6 g bid + CT	CT	8	⑥⑦⑧⑨⑩
Ma et al. (2014)	80/80	NA		56.53 ± 13.71		WBL Biotech	0.30 g/capsule	XZK 0.6 g bid + CT	CT	2	⑥⑦⑧⑨
Zhu and Li (2008)	42/42	48/36		54 (42–68)		WBL Biotech	capsule	XZK 0.6 g bid + CT	CT	24	①⑥⑦⑧⑨
Gao and Gao (2013)	47/47	24/23	26/21	64.7 ± 7.2	65.5 ± 7.6	Wepon	*Ginkgo biloba* extract 16m g/drop pill	YXY 5 drop pills tid + CT	CT	12	①③④
Jia (2014)	59/59	37/22	39/20	66.5 ± 5.6	65.9 ± 6.1	Wepon	0.063 g/drop pill	YXY 5 drop pills tid + CT	CT	4	①②
Wang and Sun (2012)	60/60	73/47		61.6 ± 7.8		Wepon	*Ginkgo biloba* extract 16m g/drop pill	YXY 5 drop pills tid + CT	CT	4	①②
Chen (2012)	85/85	60/25	63/23	56.23	53.44	Wepon	*Ginkgo biloba* extract 16m g/drop pill	YXY 5 drop pills tid + CT	CT	4	①
Zhang et al. (2011)	45/45	57/33		65.3 ± 9.6		Wepon	*Ginkgo biloba* extract 16m g/drop pill	YXY 5 drop pills tid + CT	CT	4	①②
He (2014)	55/53	78/30		65.3 ± 5.62		Wepon	*Ginkgo biloba* extract 16m g/drop pill	YXY 5 drop pills tid + CT	CT	1	①②
Sun et al. (2024)	180/180	96/84	85/95	61.32 ± 11.09	61.28 ± 10.28	Yangtze River	19.2 mg of total flavonol glycosides and 4.8 mg of terpene lactones/tablet	YXY 1 tablet tid + CT	CT	4	④⑤
Yu and Yang (2023)	35/35	17/18	16/19	64.85 ± 7.38	62.74 ± 7.81	Senke	9.6 mg of total flavonol glycosides and 2.4 mg of terpene lactones/tablet	YXY 0.08 g tid + CT	CT	4	⑩⑪
Du et al. (2018)	53/53	33/20	35/18	63.14 ± 8.38	64.73 ± 9.18	Yangtze River	19.2 mg of total flavonol glycosides and 4.8 mg of terpene lactones/tablet	YXY 1 tablet tid + CT	CT	4	④⑤⑩
Xia and Zhang (2003)	51/49	31/20	28/21	57 ± 6	56 ± 6	NA	tablet	YXY 0.08 g tid + CT	CT	8	①②③④⑤
Huang (2016)	100/100	61/39	57/43	54.55 ± 6.23	54.12 ± 6.83	Qianhui	0.025 g/drop pill	YXTZ 0.05 g tid + CT	CT	8	③④⑤⑩
Liu (2020)	55/55	33/22	31/24	58.29 ± 5.02	58.12 ± 5.66	Handian	*Ginkgo biloba* ester 0.008 g/drop pill	YXTZ 5 drop pills tid + CT	CT	4	⑪
Sheng et al. (2022)	35/35	22/13	21/14	60.38 ± 5.37	58.92 ± 4.15	Qianhui	*Ginkgo biloba* ester 0.005 g/drop pill	YXTZ 8 drop pills tid + CT	CT	4	①④⑤
Li et al. (2019)	49/49	30/19	28/21	58.91 ± 4.71	59.71 ± 6.52	Qianhui	*Ginkgo biloba* ester 0.005 g/drop pill	YXTZ 8 drop pills tid + CT	CT	4	①②③④⑤⑧
Wu (2014)	34/34	15/19	16/18	58.4 ± 7.3	58.2 ± 7.1	Handian	*Ginkgo biloba* ester 0.008 g/drop pill	YXTZ 0.05 g tid + CT	CT	12	①
Dong and Yu (2015)	60/60	34/26	37/23	63.7 ± 4.4	62.5 ± 4.1	Qianhui	*Ginkgo biloba* ester 0.005 g/drop pill	YXTZ 8 drop pills tid + CT	CT	2	②④⑤
Luo and Yu (2013)	37/37	25/12	24/13	64.8 ± 7.2	65.2 ± 6.8	Qianhui	*Ginkgo biloba* ester 0.005 g/drop pill	YXTZ 8 drop pills tid + CT	CT	2	①⑥⑦⑧
Liu et al. (2018b)	63/63	70/56		62.36 ± 1.72		NA	drop pill	YXTZ 8 drop pills tid+CT	CT	1	⑥⑦⑧⑨⑩
Peng et al. (2020)	70/70	38/32	37/33	61.29 ± 4.59	61.43 ± 4.75	Shenlong	0.15 g/dispersible tablet	YXTZ 0.15 g tid + CT	CT	8	④⑤
Song (2021)	42/42	23/19	22/20	63.12 ± 5.22	62.45 ± 5.17	Shenlong	0.15 g/dispersible tablet	YXTZ 0.15 g tid + CT	CT	8	④⑤
Li et al. (2003)	64/64	44/20	46/18	62 ± 18	65 ± 19	Meidakang	0.005 g/tablet	XDK 0.4 g bid + CT	CT	3	①②⑥⑦⑨
Huang (2022)	40/40	24/16	25/15	52.26 ± 2.25	52.31 ± 2.32	Meidakang	capsule	XDK 2 capsules bid + CT	CT	8	③④
Huang (2018)	53/53	23/30	30/23	63.0 ± 3.9	66.26 ± 3.1	Yinkeruisi	0.035 g/tablet	XDK 0.63 g bid+CT	CT	8	①③④
Zhang et al. (2012)	61/60	41/20	39/21	63.4 ± 6.9	64.7 ± 7.2	Taonan	Furosemide 0.015 g/capsule	XNST 4 capsules tid + CT	CT	2	①⑩
Ma et al. (2009)	32/32	25/7	26/6	62.5 ± 5.0	63.0 ± 5.5	Taonan	Furosemide 0.015 g/capsule	XNST 2 capsules tid + CT	CT	24	③④⑤
Wang (2015)	45/48	60/33		65.78 ± 5.9		Taonan	Furosemide 0.015 g/capsule	XNST 3 capsules tid + CT	CT	1	①
Liu and Zhu (2019)	67/67	39/28	37/30	67.9 ± 4.8	67.3 ± 4.3	Kanion	0.38 g/capsule	DZHJT 4 capsules tid + CT	CT	8	①⑩
Shen et al. (2014)	46/46	26/20	27/19	58.2 ± 8.8	57.2 ± 8.1	Kanion	0.38 g/capsule	DZHJT 4 capsules tid + CT	CT	8	②③④⑪
Yue and Zhang (2013)	30/30	16/14	18/12	61.93 ± 12.24	63.27 ± 13.16	Xinbang	0.25 g/capsule	MXK 1.0 g tid + CT	CT	12	①⑥⑦⑧
Chen and Chen (2021)	43/43	26/17	25/18	61.68 ± 3.09	61.52 ± 3.07	Duoputai	0.25 g/capsule	MXK 1.0 g tid+CT	CT	4	⑩
Luo (2013)	56/56	32/24	32/24	61	63	Xinbang	0.25 g/capsule	MXK 0.75 g tid + CT	CT	4	①
Li (2014)	40/40	22/18	21/19	45–73	47–75	Duoputai	0.25 g/capsule	MXK 1.0 g tid + CT	CT	4	①⑪
Wang et al. (2012)	30/30	15/15	14/16	68.5 (46–78)		Duoputai	0.25 g/capsule	MXK 0.75 g tid + CT	CT	4	①②
Gao (2010)	50/50	29/21	29/21	NA		Duoputai	0.25 g/capsule	MXK 1.0 g tid + CT	CT	4	①⑪
Xing and Lv (2015)	53/52	35/18	32/20	62.5 ± 10.7	61.7 ± 10.4	Duoputai	0.25 g/capsule	MXK 0.75 g tid + CT	CT	8	④⑤⑪
Chen et al. (2021)	44/42	25/19	24/18	56.03 ± 5.12	55.31 ± 5.05	Xinbang	0.25 g/capsule	MXK 1.0 g tid + CT	CT	8	②⑪
Hao (2022)	50/49	28/22	26/23	63.65 ± 5.18	62.65 ± 5.46	NA	0.25 g/capsule	MXK 1.0 g tid + CT	CT	12	②

Abbreviations: T, treatment group; C, control group; M, male; F, female; ZY, Zhenyuan oral preparation; XST, Xuesaitong oral preparation; XY, Xinyue oral preparation; DAXXK, diaoxinxuekang oral preparation; XZK, Xuezhikang oral preparation; YXY, yinxingye oral preparation; YXTZ, Yinxingtongzhi oral preparation; XDK, xindakang oral preparation; XNST, Xinnaoshutong oral preparation; DZHJT, dazhuhongjingtian oral preparation; MXK, Maixuekang oral preparation; CT, conventional treatment; NA, Not Available. ①Angina efficacy; ②Electrocardiogram (ECG) efficacy; ③Nitroglycerin dosages; ④Frequency of angina; ⑤Duration of angina (min/times); ⑥Total cholesterol (TC); ⑦Triglyceride (TG); ⑧Low-density Lipoprotein Cholesterol (LDL-C); ⑨High-density Lipoprotein Cholesterol (HDL-C); ⑩high-sensitivity C-reactive Protein (hs-CRP); ⑪Plasma viscosity (PV); ⑫Major adverse cardiovascular events (MACEs).

### 3.2 Risk of bias assessment of included studies

**FIGURE 2:**
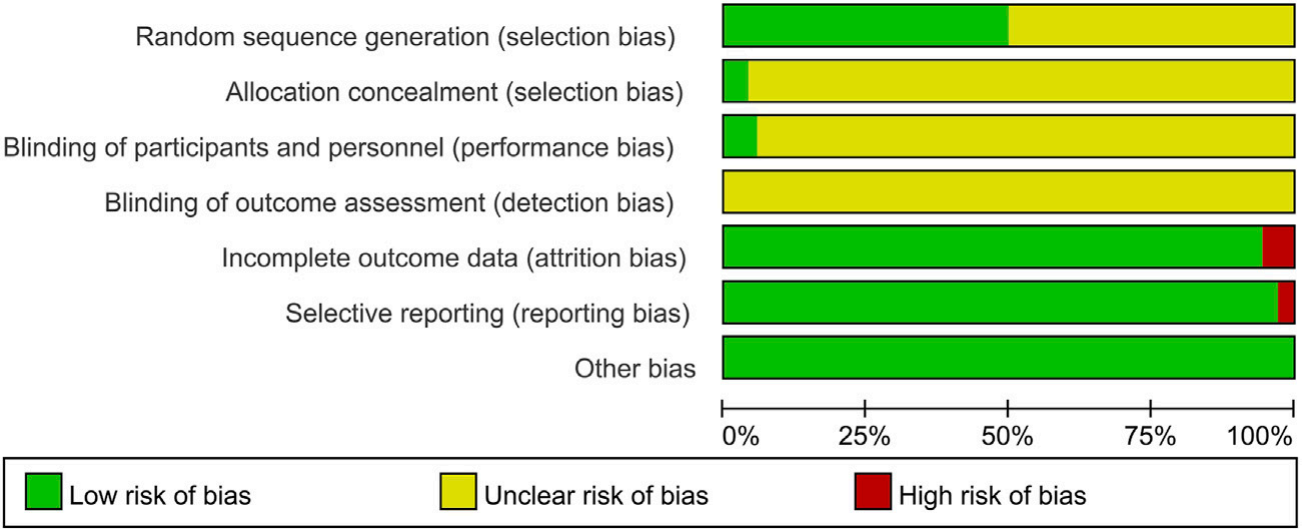
Risk of bias graph.

The 72 studies were assessed for risk of bias, and the results are detailed in Figure 2 and Supplementary Material S3. 1) random sequence generation (selection bias): 36 studies reported specific methods of generating random sequences, of which 30 studies used random number tables for random assignment (Chen, 2015; Chen and Chen, 2021; Chen et al., 2021; Dai et al., 2015; Du et al., 2018; Fan and Zhang, 2023; Gao and Gao, 2013; Huang and Zhang, 2023; Huang, 2022; Liu et al., 2013; Liu et al., 2008; Liu, 2020; Liu Y. et al., 2018; Liu and Zhu, 2019; Lu, 2022; Ma et al., 2009; Ma and Deng, 2019; Peng et al., 2020; Sheng et al., 2022; Song, 2021; Su et al., 2017; Wang and Sun, 2012; Wang, 2022; Xiong et al., 2008; Xu and Fang, 2019; Xu, 2020; Xu, 2016; Yu and Yang, 2023; Yu et al., 2023; Zhang R. et al., 2020), two studies used computers for random assignment (Dong et al., 2020; Wang et al., 2017), one study used lottery for random assignment (Huang, 2018), one study used the PROC PLAN procedure of the SAS (Statistical Analysis System) for random assignment (Duan et al., 2022), one study used stratified randomization for random assignment (Wu, 2014), and one study used coin flipping for random assignment (Hao, 2022), therefore, the risk of selection bias in the above studies was rated as “low risk”; the remaining 36 studies mentioned randomization without specifying the method, so they were rated as “unclear risk”. 2) allocation concealment (selection bias): three studies reported the use of SNOSE (sequentially numbered, opaque sealed envelopes) for allocation concealment, which was rated as “low risk” (Dong et al., 2020; Duan et al., 2022; Liu et al., 2008); The remaining studies did not mention allocation concealment programs, and were rated as “unclear risk”. 3) blinding of participants and personnel (performance bias): two studies were single-blind (Kuang et al., 2011; Wei, 2010) and two studies were double-blind (Dong et al., 2020; Duan et al., 2022), which were considered “low risk”. The remaining studies did not mention information on blinding, were rated as “unclear risk”. 4) blinding of outcome assessment (detection bias): None of the studies reported blinding of outcome assessors, so all were rated as “unclear risk”. 5) Incomplete outcome data (attrition bias): four studies (Dong et al., 2020; Duan et al., 2022; Ma and Deng, 2019; Wang et al., 2017) were rated as “high risk” for incomplete outcome data, while the remaining studies with no incomplete outcome data were rated as “low risk”. 6) selective reporting (reporting bias): two studies (Lin et al., 2009; Liu Q. et al., 2018) had selective reporting resulting in incomplete outcome data and were rated as “high risk”; The remaining studies reported the expected outcome measures according to the experimental protocol and were rated as “low risk”. 7) Other bias: No other significant biases were identified, so all studies were rated “low risk” for other bias.

### 3.3 Results of the network meta-analysis

#### 3.3.1 Primary outcome measures

##### 3.3.1.1 Angina efficacy

**FIGURE 3:**
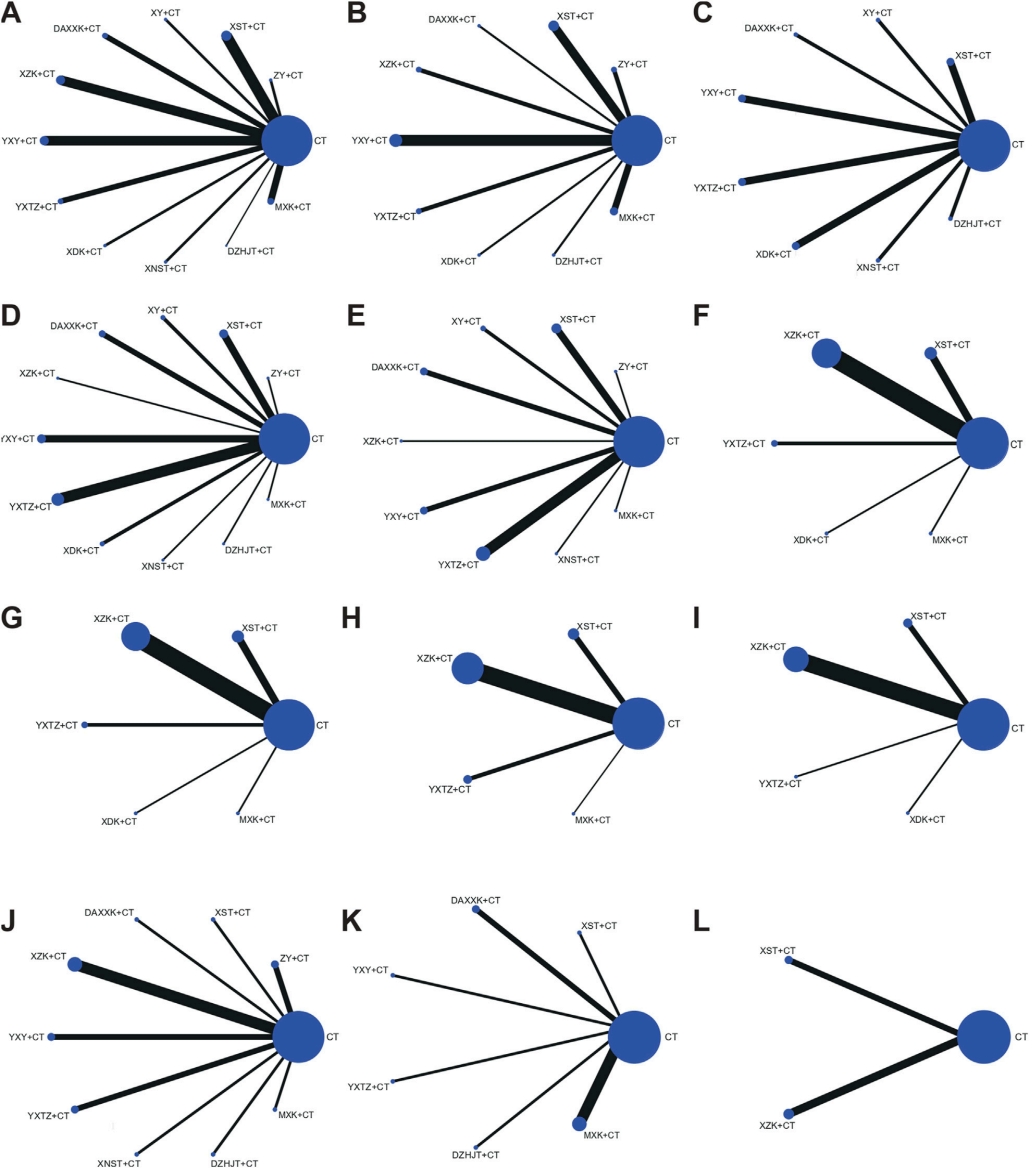
Networks plot for different outcomes. The line width indicates the number of studies directly comparing interventions. The node size reflects the total number of participants across studies. **(A)** Angina efficacy; **(B)** ECG efficacy; **(C)** Nitroglycerin dosages; **(D)** Frequency of angina; **(E)** Duration of angina; **(F)** Total cholesterol (TC); **(G)** Triglyceride (TG); **(H)** Low-density Lipoprotein Cholesterol (LDL-C); **(I)** High-density Lipoprotein Cholesterol (HDL-C); **(J)** high-sensitivity C-reactive protein (hs-CRP); **(K)** Plasma viscosity (PV); **(L)** Major adverse cardiovascular events (MACEs).

**FIGURE 4:**
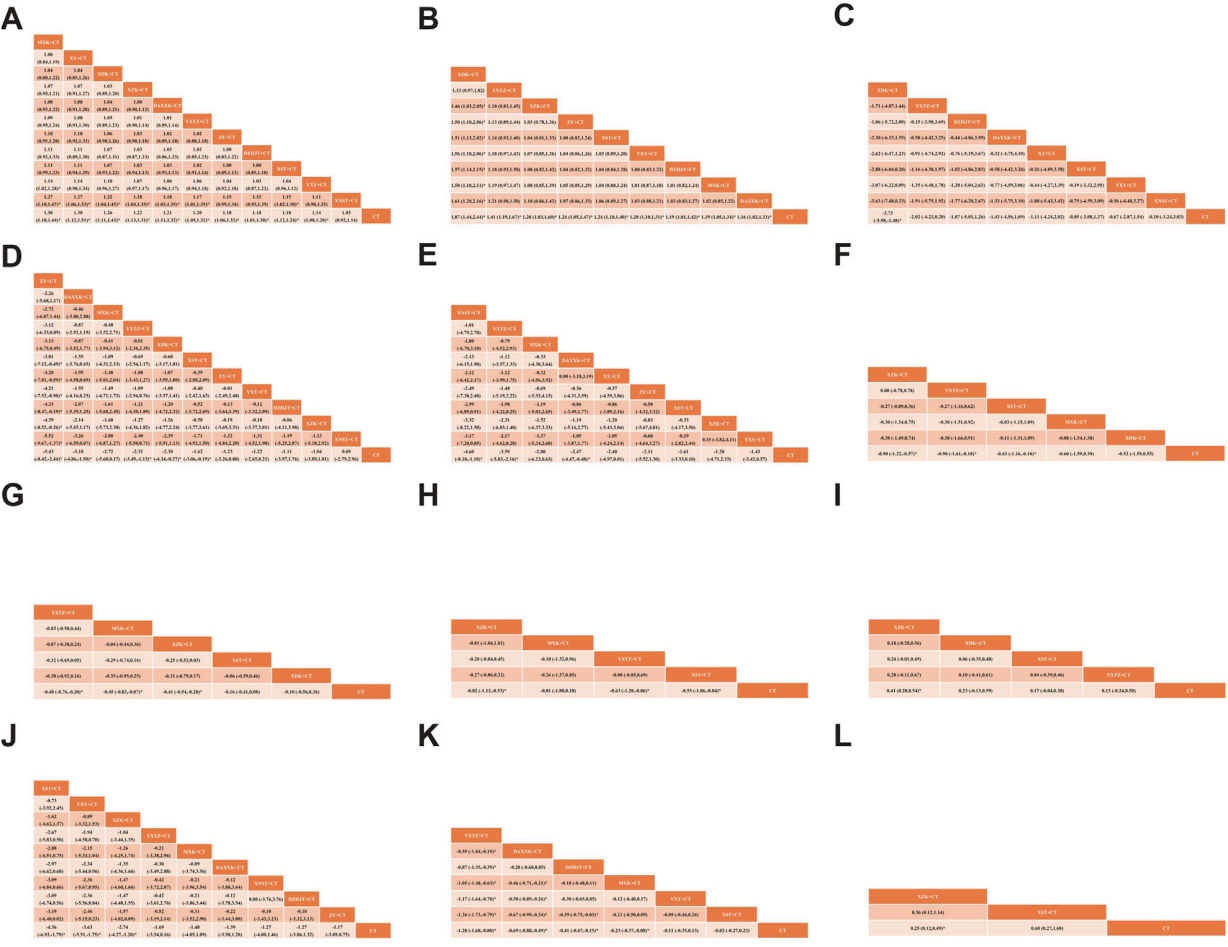
League table of pairwise treatment comparisons with 95% confidence intervals (CIs). **(A)** Angina efficacy; **(B)** ECG efficacy; **(C)** Nitroglycerin dosages; **(D)** Frequency of angina; **(E)** Duration of angina; **(F)** Total cholesterol (TC); **(G)** Triglyceride (TG); **(H)** Low-density Lipoprotein Cholesterol (LDL-C); **(I)** High-density Lipoprotein Cholesterol (HDL-C); **(J)** high-sensitivity C-reactive protein (hs-CRP); **(K)** Plasma viscosity (PV); **(L)** Major adverse cardiovascular events (MACEs).

**FIGURE 5:**
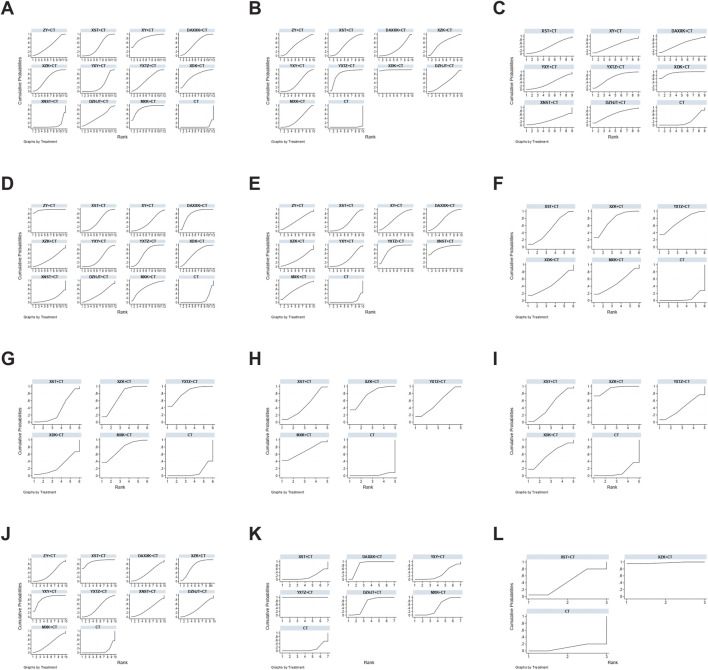
SUCRA rankings for treatments in unstable angina pectoris based on different outcomes. **(A)** Angina efficacy; **(B)** ECG efficacy; **(C)** Nitroglycerin dosages; **(D)** Frequency of angina; **(E)** Duration of angina; **(F)** Total cholesterol (TC); **(G)** Triglyceride (TG); **(H)** Low-density Lipoprotein Cholesterol (LDL-C); **(I)** High-density Lipoprotein Cholesterol (HDL-C); **(J)** high-sensitivity C-reactive protein (hs-CRP); **(K)** Plasma viscosity (PV); **(L)** Major adverse cardiovascular events (MACEs).

A total of 44 studies (Chen, 2015; Chen, 2012; Dai et al., 2015; Dai et al., 1999; Fan and Zhang, 2023; Feng et al., 2009; Gao, 2010; Gao and Gao, 2013; Ge and Zhao, 2010; He, 2014; Huang and Zhang, 2023; Huang, 2018; Jia, 2014; Kuang et al., 2011; Li, 2014; Li et al., 2003; Li et al., 2019; Lin et al., 2009; Liu, 2015; Liu et al., 2008; Liu Q. et al., 2018; Liu and Zhu, 2019; Luo and Yu, 2013; Luo, 2013; Ren and Peng, 2006; Sheng et al., 2022; Su et al., 2017; Wang and Sun, 2012; Wang, 2015; Wang, 2022; Wang et al., 2012; Wei, 2010; Wu, 2014; Xia and Zhang, 2003; Xu and Fang, 2019; Xu, 2020; Xu, 2016; Yu, 2010; Yu et al., 2023; Yue and Zhang, 2013; Zhang et al., 2011; Zhang R. et al., 2020; Zhang et al., 2012; Zhu and Li, 2008) reported angina efficacy involving 11 SSTCM-OPs. The evidence plots are shown in Figure 3A. Figure 4A showed that 10 SSTCM-OPs + CT were more effective than CT alone (p < 0.05), including: MXK + CT (RR = 1.30, CI: 1.18, 1.44), XY + CT (RR = 1.30, CI: 1.12, 1.51), XDK + CT (RR = 1.26, CI: 1.11, 1.43), XZK + CT (RR = 1.22, CI: 1.13, 1.31), DAXXK + CT (RR = 1.21, CI: 1.11, 1.32), YXTZ + CT (RR = 1.20, CI: 1.09, 1.32), ZY + CT (RR = 1.18, CI: 1.06, 1.32), DZHJT + CT (RR = 1.18, CI: 1.01, 1.38), XST + CT (RR = 1.18, CI: 1.12, 1.24), YXY + CT (RR = 1.14, CI: 1.08, 1.20). According to the results of the SUCRA ranking, MXK + CT (86.6%) was the best treatment, followed by XY + CT (82.1%), XDK + CT (72.5%), XZK + CT (61.3%), DAXXK + CT (59.1%), YXTZ + CT (55.1%), ZY + CT (47.9%), DZHJT + CT (47.7%), XST + CT (46%), YXY + CT (30.2%), XNST + CT (8.2%) in Figure 5A and Supplementary Material S4. The certainty of this evidence was assessed as moderate to very low overall, with details provided in Supplementary Material S3.

##### 3.3.1.2 ECG efficacy

A total of 21 studies (Chen et al., 2021; Dai et al., 1999; Dong and Yu, 2015; Feng et al., 2009; Ge and Zhao, 2010; Hao, 2022; He, 2014; Jia, 2014; Li et al., 2003; Li et al., 2019; Liu Q. et al., 2018; Ren and Peng, 2006; Shen et al., 2014; Wang and Sun, 2012; Wang, 2022; Wang et al., 2012; Wei, 2010; Xia and Zhang, 2003; Xu and Fang, 2019; Yu, 2010; Zhang et al., 2011) reported the ECG efficacy involving nine SSTCM-OPs. The evidence plots are shown in Figure 3B. According to Figure 4B, the NMA results showed that the ECG efficacy of the included 9 SSTCM-OPs + CT was superior to that of single used CT alone (p < 0.05), including: XDK + CT (RR = 1.87, CI: 1.44, 2.44), YXTZ + CT (RR = 1.41, CI: 1.19, 1.67), XZK+CT (RR = 1.28, CI: 1.03, 1.60), ZY + CT (RR = 1.24, CI: 1.05, 1.47), XST + CT (RR = 1.24 CI: 1.10, 1.40), YXY + CT (RR = 1.20, CI: 1.10, 1.31), DZHJT + CT (RR = 1.19, CI: 1.01, 1.42), MXK + CT (RR = 1.19, CI: 1.05, 1.34), DAXXK + CT (RR = 1.16, CI: 1.02, 1.33). In addition, according to the results of the NMA, XDK + CT was more advantageous in improving ECG efficacy as it was statistically significant when compared to all other interventions (p < 0.05) except YXTZ + CT in Figure 4B. Similarly, the results of the SUCRA ranking suggest that XDK + CT (99.3%) was the best treatment, followed by YXTZ + CT (81.3%), XZK + CT (60.7%), ZY + CT (52.6%), XST + CT (52.6%), YXY + CT (41.3%), DZHJT + CT (40.7%), MXK + CT (38%), DAXXK + CT (33%) in Figure 5B and Supplementary Material S4. The certainty of this evidence was assessed as moderate to very low overall, with details provided in Supplementary Material S3.

#### 3.3.2 Secondary outcome measures

##### 3.3.2.1 Nitroglycerin dosages

A total of 12 studies (Gao and Gao, 2013; Huang and Zhang, 2023; Huang, 2016; Huang, 2022; Huang, 2018; Li et al., 2019; Ma et al., 2009; Shen et al., 2014; Xia and Zhang, 2003; Yu et al., 2023; Zhang R. et al., 2020; Zhao, 2002) reported the nitroglycerin dosages involving 8 SSTCM-OPs. The evidence plots are shown in Figure 3C. Figure 4C showed that only 1 SSTCM-OP combined with CT reduced nitroglycerin dosages compared to single used CT (p < 0.05), which was XDK + CT (SMD = −3.73, CI: −5.98, −1.48). According to the results of the SUCRA ranking, XDK + CT (92.3%) was the best treatment, followed by YXTZ + CT (66.6%), DZHJT + CT (61%), DAXXK + CT (54.5%), XY + CT (47.9%), XST + CT (42.2%), YXY + CT (37.6%), XNST + CT (28.6%) in Figure 5C and Supplementary Material S4. The certainty of this evidence was assessed as very low overall, with details provided in Supplementary Material S3.

##### 3.3.2.2 Frequency of angina

A total of 26 studies (Chen et al., 2021; Dong and Yu, 2015; Du et al., 2018; Duan et al., 2022; Gao and Gao, 2013; Huang and Zhang, 2023; Huang, 2016; Huang, 2022; Huang, 2018; Kuang et al., 2011; Lu, 2022; Ma et al., 2009; Ma and Deng, 2019; Peng et al., 2020; Shen et al., 2014; Sheng et al., 2022; Song, 2021; Sun et al., 2024; Wang, 2022; Wei, 2010; Xia and Zhang, 2003; Xing and Lv, 2015; Xu, 2016; Xu and Fang, 2019; Yu et al., 2023; Zhang R. et al., 2020) reported the frequency of angina involving 11 SSTCM-OPs. The evidence plots are shown in Figure 3D. Figure 4D showed that 5 SSTCM-OPs + CT reduced the number of angina episodes compared to CT (p < 0.05), including: ZY + CT (SMD = −5.43, CI: −8.42, −2.44), DAXXK + CT (SMD = −3.18, CI: −4.86, −1.50), YXTZ + CT (SMD = −2.31, CI: −3.49, −1.13), XDK + CT (SMD = −2.30, CI: −4.34, −0.27), XST + CT (SMD = −1.62, CI: −3.06, −0.19). However, XNST + CT (SMD = 0.09, CI: −2.79, 2.96) did not reduce the frequency of angina, and more large-sample RCTs are needed for data correction. According to the results of the SUCRA ranking, ZY + CT (96.9%) was the best treatment, followed by DAXXK + CT (79.7%), MXK + CT (67.9%), YXTZ + CT (64.4%), XDK + CT (62.7%), XST + CT (47.5%), XY + CT (39.1%), YXY + CT (38.6%), DZHJT + CT (38.1%), XZK + CT (36.5%), XNST + CT (17.5%) in Figure 5D and Supplementary Material S4. The certainty of this evidence was assessed as low to very low overall, with details provided in Supplementary Material S3.

##### 3.3.2.3 Duration of angina

A total of 22 studies (Dong and Yu, 2015; Du et al., 2018; Duan et al., 2022; Huang and Zhang, 2023; Huang, 2016; Kuang et al., 2011; Li et al., 2019; Lu, 2022; Ma et al., 2009; Ma and Deng, 2019; Peng et al., 2020; Sheng et al., 2022; Song, 2021; Sun et al., 2024; Wang, 2022; Wei, 2010; Xia and Zhang, 2003; Xing and Lv, 2015; Xu and Fang, 2019; Xu, 2016; Yu et al., 2023; Zhang R. et al., 2020) involving 9 SSTCM-OPs with an improvement in duration of angina were reported. The evidence plots are shown in Figure 3E. According to Figure 4E, the results of the NMA showed that 3 SSTCM-OPs + CT reduced the duration of angina compared to CT (p < 0.05), including: XNST + CT (MD = −4.60, CI: −8.10, −1.10), YXTZ + CT (MD = −3.59, CI: −5.03, −2.16), DAXXK + + CT (MD = −2.47, CI: −4.47, −0.48). The remaining 6 SSTCM-OPs showed no statistically significant improvement in the duration of angina. The results of the SUCRA ranking suggested that XNST + CT (86.2%) was the best treatment, followed by YXTZ + CT (79.2%), MXK + CT (60.1%), DAXXK + CT (56.4%), XY + CT (56.3%), ZY + CT (48.5%), XST + CT (38%), XZK + CT (35%), YXY + CT (34.4%) in Figure 5E and Supplementary Material S4. The certainty of this evidence was assessed as low to very low overall, with details provided in Supplementary Material S3.

##### 3.3.2.4 TC

A total of 18 studies (Chen, 2015; Cui et al., 2014; Cui and Li, 2016; Dai et al., 1999; Dong et al., 2020; Duan et al., 2022; Li et al., 2003; Lin et al., 2009; Liu et al., 2008; Liu Y. et al., 2018; Luo and Yu, 2013; Ma and Deng, 2019; Ma et al., 2014; Su et al., 2017; Wang et al., 2017; Xiong et al., 2008; Yue and Zhang, 2013; Zhu and Li, 2008) reported changes in TC levels involving 5 SSTCM-OPs. The evidence plots are shown in Figure 3F. For TC levels, Figure 4F showed that 3 SSTCM-OPs + CT had the ability in cutting down TC levels compared to CT (p < 0.05), including: XZK + CT (MD = −0.90, CI: −1.22, −0.57), YXTZ + CT (MD = −−0.90, CI: −1.61, −0.18), XST + CT (MD = −0.63, CI: −1.16, −0.10), and the results suggested that XZK + CT vs. YXTZ + CT (MD = 0.00, CI: −0.78, 0.78), both of which were comparable in their ability to cut down TC levels. According to the results of the SUCRA ranking, XZK + CT (91.6%) was the best treatment to cut down TC levels, followed by YXTZ + CT (71.8%), XST + CT (52.1%), MXK + CT (49.8%), and XDK + CT (45.1%) in Figure 5F and Supplementary Material S4. The certainty of this evidence was assessed as low to very low overall, with details provided in Supplementary Material S3.

##### 3.3.2.5 TG

A total of 18 studies (Chen, 2015; Cui et al., 2014; Cui and Li, 2016; Dai et al., 1999; Dong et al., 2020; Duan et al., 2022; Liu et al., 2013; Liu et al., 2008; Liu Y. et al., 2018; Luo and Yu, 2013; Ma and Deng, 2019; Ma et al., 2014; Su et al., 2017; Wang et al., 2017; Xiong et al., 2008; Yue and Zhang, 2013; Zhang R. et al., 2020; Zhu and Li, 2008) reported changes in TG levels involving 5 SSTCM-OPs. The evidence plots are shown in Figure 3G. For TG levels, Figure 4G showed that 3 SSTCM-OPs + CT had the ability in cutting down TG levels compared to CT (p < 0.05), including: YXTZ + CT (MD = −0.48, CI: −0.76, −0.20), MXK + CT (MD = −0.45, CI: −0.83, −0.07), XZK + CT (MD = −0.41, CI: −0.54, −0.28). According to the results of the SUCRA ranking, YXTZ + CT (82.2%) was the best treatment to cut down TG levels, followed by MXK + CT (75.1%), XZK + CT (72.5%), XST + CT (34%), and XDK + CT (27.3%) in Figure 5G and Supplementary Material S4. The certainty of this evidence was assessed as low to very low overall, with details provided in Supplementary Material S3.

##### 3.3.2.6 LDL-C

A total of 20 studies (Chen, 2015; Cui et al., 2014; Cui and Li, 2016; Dai et al., 1999; Dong et al., 2020; Duan et al., 2022; Li et al., 2019; Lin et al., 2009; Liu et al., 2013; Liu et al., 2008; Liu Y. et al., 2018; Luo and Yu, 2013; Ma and Deng, 2019; Ma et al., 2014; Su et al., 2017; Wang et al., 2017; Wang et al., 2006; Xiong et al., 2008; Yue and Zhang, 2013; Zhu and Li, 2008) reported changes in LDL-C levels involving 4 SSTCM-OPs. The evidence plots are shown in Figure 3H. For LDL-C levels, Figure 4H showed that 3 SSTCM-OPs + CT had a therapeutic effect in cutting down LDL-C levels compared to CT (p < 0.05), including: XZK + CT (MD = −0.82, CI: 1.12, −0.53), YXTZ + CT (MD = −0.63, CI: −1.20, −0.06), XST + CT (MD = −0.55, CI: −1.06, −0.04). According to the results of the SUCRA ranking, XZK + CT (76.8%) was the best treatment in reducing LDL-C levels, followed by MXK + CT (67.9%), YXTZ + CT (55.8%), and XST + CT (47.3%) in Figure 5H and Supplementary Material S4. The certainty of this evidence was assessed as very low overall, with details provided in Supplementary Material S3.

##### 3.3.2.7 HDL-C

A total of 14 studies (Chen, 2015; Chen et al., 2021; Cui et al., 2014; Dai et al., 1999; Duan et al., 2022; Liu et al., 2008; Liu Y. et al., 2018; Ma and Deng, 2019; Ma et al., 2014; Su et al., 2017; Wang et al., 2017; Wang et al., 2006; Xiong et al., 2008; Zhu and Li, 2008) reported changes in HDL-C levels involving 4 SSTCM-OPs. The evidence plots are shown in Figure 3I. For HDL-C levels, Figure 4I showed that only XZK + CT (MD = 0.41, CI: 0.28, 0.54) improved HDL-C levels compared to CT (p < 0.05). The remaining three SSTCM-OPs had the possibility of improving HDL-C, but the difference was not significant. According to the results of the SUCRA ranking, XZK + CT (92.5%) was the best treatment in improving HDL-C levels, followed by XDK + CT (59.1%), XST + CT (47.8%), YXTZ + CT (40.2%) in Figure 5I and Supplementary Material S4. The certainty of this evidence was assessed as very low overall, with details provided in Supplementary Material S3.

##### 3.3.2.8 hs-CRP

A total of 15 studies (Chen and Chen, 2021; Cui et al., 2014; Cui and Li, 2016; Du et al., 2018; Fan and Zhang, 2023; Huang, 2016; Liu et al., 2013; Liu Q. et al., 2018; Liu Y. et al., 2018; Liu and Zhu, 2019; Wang, 2022; Xiong et al., 2008; Yu and Yang, 2023; Yu et al., 2023; Zhang et al., 2012) reported hs-CRP improvement involving 9 SSTCM-OPs. The plots of the evidence network are shown in Figure 3J. According to Figure 4J, the NMA results showed that 3 SSTCM-OPs + CT significantly reduced hs-CRP levels compared to CT (P < 0.05), including: XST + CT (MD = −4.36, CI: −6.93, −1.79), YXY + CT (MD = −3.63, CI: −5.51, −1.75), XZK + CT (MD = −2.74, CI: −4.27, −1.20). The remaining six SSTCM-OPs offered the possibility of reducing hs-CRP, but the difference was not significant. According to the results of the SUCRA ranking, XST + CT (91.6%) was the best treatment for reducing hs-CRP to improve coronary inflammation, followed by YXY + CT (84.7%), XZK + CT (69.8%), YXTZ + CT (47%), MXK + CT (43.7%), DAXXK + CT (41.5%), XNST + CT (38.9%), DZHJT + CT (38.3%), ZY + CT (35.9%) in Figure 5J and Supplementary Material S4. The certainty of this evidence was assessed as moderate to very low overall, with details provided in Supplementary Material S3.

##### 3.3.2.9 PV

A total of 10 studies (Chen et al., 2021; Feng et al., 2009; Gao, 2010; Li, 2014; Liu, 2020; Shen et al., 2014; Xing and Lv, 2015; Xu, 2020; Xu, 2016; Yu and Yang, 2023) involving six SSTCM-OPs with a change in PV were reported. The plots of the evidence network are shown in Figure 3K. Figure 4K showed that 4 SSTCM-OPs + CT made a change in PV compared to single used CT (p < 0.05), including: YXTZ + CT (MD = −1.28, CI: −1.68, −0.88), DAXXK + CT (MD = −0.69, CI: −0.88, −0.49), DZHJT + CT (MD = −0.41, CI: −0.67, −0.15), MXK + CT (MD = −0.23, CI: −0.37, −0.08). At the same time, YXTZ + CT was statistically different compared to all other 5 SSTCM-OPs in reducing PV levels (p < 0.05). The results of the SUCRA ranking demonstrated that YXTZ + CT (99.9%) was the best treatment, followed by DAXXK + CT (82.6%), DZHJT + CT (64.4%), MXK + CT (46.8%), YXY + CT (29.6%), and XST + CT (16.2%) in Figure 5K and Supplementary Material S4. The certainty of this evidence was assessed as moderate to very low overall, with details provided in Supplementary Material S3.

##### 3.3.2.10 MACEs

A total of seven studies reported the incidence of MACEs involving two SSTCM-OPs, XST + CT (Duan et al., 2022; Yu, 2010; Zhao, 2002) and XZK + CT (Chen, 2015; Dai et al., 2015; Lin et al., 2009; Liu et al., 2013). The evidence plots are shown in Figure 3L. Figure 4L showed that XZK + CT (RR = 0.25, CI: 0.12, 0.49) was statistically significant in reducing the incidence of MACEs compared to CT (p < 0.05). According to the results of the SUCRA ranking, XZK + CT (97.6%) was a recommended intervention in clinical practice to reduce the incidence of MACEs, followed by XST + CT (42.4%) in Figure 5L and Supplementary Material S4. The certainty of this evidence was assessed as moderate to very low overall, with details provided in Supplementary Material S3.

#### 3.3.3 Cluster analysis

**FIGURE 6:**
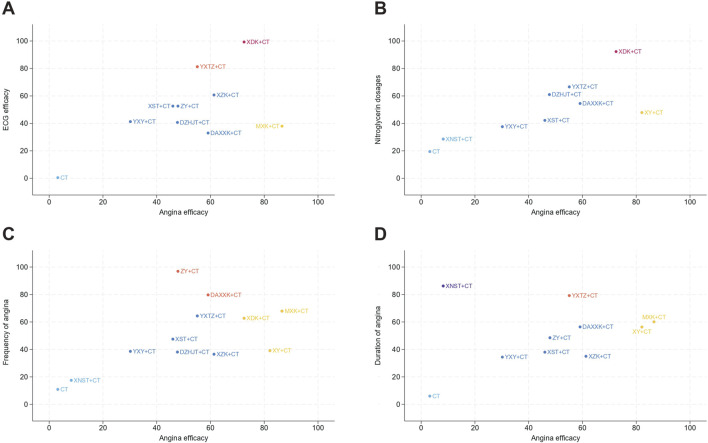
Cluster analysis for treatments in unstable angina pectoris based on different outcomes. **(A)** Angina efficacy and ECG efficacy; **(B)** Angina efficacy and Nitroglycerin dosages; **(C)** Angina efficacy and Frequency of angina; **(D)** Angina efficacy and Duration of angina.

Cluster analysis was established by dividing the angina efficacy with the ECG efficacy, the frequency of angina, the duration of angina, and the nitroglycerin dosages, respectively. The different levels of intervention were delineated by different colors. According to Figures 6A,B, the best effects in terms of improvement of the angina efficacy and the ECG efficacy were obtained with XDK + CT, and the same results were observed in improving angina efficacy and the nitroglycerin dosages as variables. Figures 6C,D revealed that no interventions were distributed in the upper right corner for the combinations of angina efficacy vs. duration of angina, and angina efficacy vs. frequency of angina. However, we recommend MXK+CT as a clinically valuable intervention, since the SUCRA values for MXK + CT exceeded 60% for all three outcome indicators.

#### 3.3.4 Adverse reactions

A total of 33 studies reported the occurrence of adverse reactions, of which 13 studies (Du et al., 2018; Gao, 2010; Huang, 2018; Kuang et al., 2011; Li et al., 2003; Li et al., 2019; Luo and Yu, 2013; Luo, 2013; Ma and Deng, 2019; Wang, 2015; Wang et al., 2012; Yue and Zhang, 2013; Zhang et al., 2012) did not observe any significant adverse reactions and 20 studies (Chen, 2015; Duan et al., 2022; Fan and Zhang, 2023; Hao, 2022; Huang, 2022; Liu et al., 2013; Liu, 2020; Liu Y. et al., 2018; Ma et al., 2014; Peng et al., 2020; Sheng et al., 2022; Song, 2021; Su et al., 2017; Wang, 2022; Xu and Fang, 2019; Yu, 2010; Yu and Yang, 2023; Zhang R. et al., 2020; Zhao, 2002; Zhu and Li, 2008) reported adverse reactions during treatment. These adverse reactions included dizziness, headache, flushing, gastrointestinal distress, stomach upset, nausea, vomiting, abdominal pain, abdominal distension, diarrhea, rash, subcutaneous hemorrhage, gingival bleeding, bleeding, fatigue, tachycardia, hypotension, and abnormalities in liver function. Due to substantial heterogeneity in adverse reaction reporting criteria across studies, quantitative meta-analysis of safety outcomes was not feasible; thus, only descriptive summaries were provided. The incidence of adverse reactions in the treatment group was 97/1754 (5.53%) compared to 76/1749 (4.35%) in the control group in Supplementary Material S5.

### 3.4 Sensitivity analysis

Although the majority of included studies utilized capsule formulations, heterogeneity in dosage forms (e.g., tablets, drop pills) was observed, which may introduce confounding variables, thereby limiting the generalizability of the study findings. Therefore, a sensitivity analysis was conducted on the primary outcomes to strengthen the credibility of these results, with details in Supplementary Material S3. This study confirmed the robustness of capsule formulations in improving both angina efficacy and ECG efficacy, with consistent results across different analytical approaches.

### 3.5 Subgroup analysis

Subgroup analyses were performed according to treatment courses (≤4 weeks *versus* >4 weeks) to evaluate both angina efficacy and ECG efficacy. Heterogeneity was detected across these subgroups, with results indicating that intervention duration might serve as a source of heterogeneity, as detailed in Supplementary Material S3. This suggests that the length of treatment may affect outcome consistency in this NMA, highlighting the importance of considering treatment course stratification in clinical applications.

### 3.6 Meta-regression analysis

The meta-regression analysis identified no significant association between XZK + CT treatment course (4–48 weeks) and LDL-C reduction (*β* = 0.098, CI: −0.701, 0.879) in Supplementary Material S3. We calculated the central value (18 weeks), which served as a mathematical midpoint to anchor regression estimation. The reference effect at the 18-week reference point showed no significant variation across treatment courses. These findings suggest that it does not significantly contribute to between-study heterogeneity, which is predominantly influenced by non-time-related factors.

### 3.7 Publication bias

**FIGURE 7:**
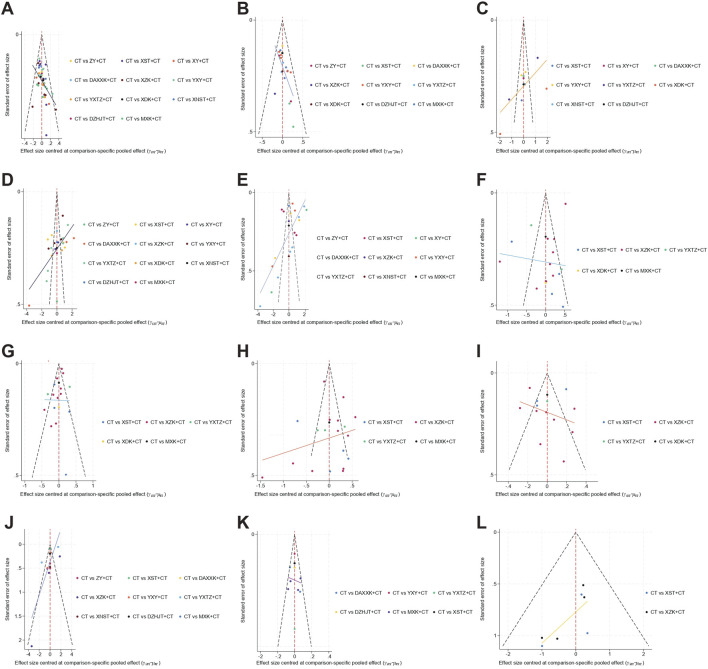
Funnel plots for treatments in unstable angina pectoris based on different outcomes. **(A)** Angina efficacy; **(B)** ECG efficacy; **(C)** Nitroglycerin dosages; **(D)** Frequency of angina; **(E)** Duration of angina; **(F)** Total cholesterol (TC); **(G)** Triglyceride (TG); **(H)** Low-density Lipoprotein Cholesterol (LDL-C); **(I)** High-density Lipoprotein Cholesterol (HDL-C); **(J)** high-sensitivity C-reactive protein (hs-CRP); **(K)** Plasma viscosity (PV); **(L)** Major adverse cardiovascular events (MACEs).

Figures 7A–L shows the results of the publication bias assessment, where points of different colors represent different direct comparisons between interventions. Publication bias was assessed across 12 outcomes using comparison-adjusted funnel plots. The results showed that the comparison-adjusted funnel plots for angina efficacy, ECG efficacy, hs-CRP, PV, TG, HDL-C, and MACEs showed acceptable symmetry, suggesting that a small-study effect was less likely. The comparison-adjusted funnel plots for frequency of angina, duration of angina, nitroglycerin dosages, TC, and LDL-C showed poor symmetry, suggesting that publication bias may exist. The observed publication bias may be attributed to quality differences, small sample size, dose, and dosage form, therefore, more homogeneous and large-sample studies are needed.

## 4 Discussion

### 4.1 Clinical significance

This study included 72 studies involving 7,360 participants, providing a comprehensive evaluation of the efficacy and safety of 11 SSTCM-OPs in patients with UAP. We evaluated a total of 12 outcomes across three categories: 1) angina-related efficacy, including angina efficacy, ECG efficacy, nitroglycerin dosages, frequency of angina, and duration of angina; 2) blood lipid levels, covering TC, TG, LDL-C, and HDL-C; 3) other outcomes, including hs-CRP, PV, and MACEs.

#### 4.1.1 Angina-related efficacy

According to the research findings, MXK + CT was the most effective in improving angina efficacy and also ranked among the top three in terms of duration of angina and frequency of angina. Hirudin, the main metabolite of MXK, is a natural thrombin-specific inhibitor. It has been shown to promote angiogenesis, reduce thrombosis, and ameliorate hypoxia-induced damage to cardiac microvascular endothelial cells (Fan et al., 1992; Guo et al., 2011; Lin et al., 2018; Liu et al., 2024b). Studies (Wang J. et al., 2023; Zhang H. et al., 2020) have shown that hirudin exerts its cardioprotective effects and slows atherosclerosis progression through lipid modulation and induction of apoptosis in abnormally proliferating vascular smooth muscle cells. Although hirudinidae-based preparations exhibit minimal toxicity at standard therapeutic dosages, adverse reactions, including allergic reactions and hemorrhagic tendencies, were observed in long-term users (Yang et al., 2024).

XDK + CT and YXTZ + CT were ranked as the top two interventions in terms of improving ECG efficacy and reducing nitroglycerin dosages, representing the most reliable methods for objectively evaluating the improvement of angina. Furthermore, YXTZ + CT ranked second in terms of improving the duration of angina, while XDK + CT performed well in improving the angina efficacy. Previous studies have shown that XDK can reduce myocardial ischemia/reperfusion injury by improving the energy metabolism of ATP in ischemic cardiomyocytes, and has a certain effect on antiplatelet aggregation (Cheng et al., 2003; Zeng et al., 2013). Current studies have demonstrated a favorable safety profile of its source botanical drug (*Hippophae rhamnoides L.*); however, limited evidence exists on its synergistic effects or interactions with conventional cardiovascular drugs (Chen et al., 2023). Modern pharmacological research reveals that YXTZ (alias: *Ginkgo biloba* extract 50, GBE50), besides inhibiting myocardial ischemia/reperfusion injury and anti-arrhythmic effects (Liu et al., 2021; Wang X. et al., 2023), also improves hemodynamics, thereby reducing myocardial pathological damage (Jiang et al., 2017; Li et al., 2013).

ZY + CT demonstrated the most significant effect in reducing the frequency of angina. However, given the limited number of studies included in the analysis, these findings should be interpreted with caution. ZY refers to a class of saponins extracted from the fruits of *Panax ginseng C.A.Mey.*, a traditional medicinal botanical drug, with major plant-derived metabolites including ginsenosides such as Rg1 and Re (Xie et al., 2009). Based on the principles of TCM, Panax ginseng is believed to invigorate *Qi*, promote blood circulation, calm the mind, and stabilize the heart. As a commonly prescribed herbal remedy, it has been widely used in the treatment of chest stuffiness and pains, palpitations, chest tightness, fatigue, and neurasthenia, thereby underpinning its therapeutic efficacy in cardiovascular diseases (Jia et al., 2023; Xiang et al., 2008).

XNST + CT was ranked as the most effective in improving the duration of angina. Xinnaoshutong capsule is derived from *Tribulus terrestris L.* through the extraction of gross saponins, commonly known as Gross Saponins of Tribulus Terrestris (GSTT) (Zhang et al., 2010). Modern studies (Guo et al., 2006; Shi et al., 2009; Zhang et al., 2010) have highlighted the cardioprotective effects of GSTT, including its ability to mitigate atherosclerosis, reduce cardiomyocyte apoptosis, and counteract ventricular remodeling. These effects likely contribute to the therapeutic potential of Xinnaoshutong capsule in improving heart function and alleviating ischemic-related symptoms. Current research on its cardiovascular mechanisms and drug-drug interactions remains limited. Furthermore, the herbal toxicity profiles of XNST are yet unclear (Ștefănescu et al., 2020). Thus, studies on correlations, pharmacokinetics of key metabolites, and pharmacodynamic interactions are urgently needed to inform clinical safety and efficacy.

Integrated with the cluster analysis results illustrated in Figures 6A–D, MXK + CT performed better in improving the angina efficacy and the duration of angina, the angina efficacy and the frequency of angina, making it one of the most clinically recommended interventions. XDK + CT, YXTZ + CT, ZY + CT, and XNST + CT exhibited favorable performance in the corresponding aspects, providing clinical physicians with viable options for decision-making in relevant clinical scenarios.

#### 4.1.2 Blood lipid levels

XZK + CT ranked first in improving TC, LDL-C, and HDL-C levels, and although it ranked third in TG reduction, it showed no clinically significant differences compared to YXTZ + CT (MD = 0.07, CI: −0.24, 0.38) and MXK + CT (MD = 0.04, CI: −0.36, 0.44), thus supporting its role in comprehensive lipid management. However, the results of this NMA suggest that it ranks relatively low in terms of efficacy in improving angina-related outcomes. XZK is primarily derived from special fermented red yeast, which is abundant in bioactive metabolites like hydroxymethylglutaryl-coenzyme A (HMG-CoA) reductase inhibitor (Wang et al., 2015). Its lipid-regulating effect can reach the level of medium-strength statin drugs, while maintaining a relatively high safety profile (Li et al., 2021). According to research statistics, the adverse reactions of red yeast rice preparations mainly involve general disorders and administration site reactions, gastrointestinal disorders, musculoskeletal and connective tissue disorders, and adverse events related to medical examinations or laboratory indicators (Raschi et al., 2018).

The results of this study demonstrated that YXTZ + CT was equally efficacious in improving blood lipid levels as it was in improving angina-related outcomes. Specifically, it ranked first in reducing TG levels and second in lowering TC levels. Studies (Chen et al., 2010; Gu et al., 2009) show that YXTZ’s reduction in TC and TG levels is associated with modulation of the expression levels of lipid metabolism-related genes.

MXK + CT also exhibited superiority in improving blood lipid levels, and it ranked second in improving TG and LDL-C levels. Significantly, MXK + CT showed clinically insignificant differences compared to XZK + CT (MD = 0.01, CI: −1.01, 1.04) in terms of LDL-C levels. Modern research has demonstrated that MXK protects the vascular endothelium and inhibits the occurrence and development of atherosclerosis through multiple mechanisms. These include regulating lipid metabolism, reducing the level of serum ox-LDL, correcting the disorder of NO metabolism, and downregulating the expression of specific receptors such as LOX-1 and ICA-1 (Gao et al., 2013; Hu et al., 2014). As is evident from this NMA, both YXTZ + CT and MXK + CT demonstrate remarkable performance in improving angina-related curative effects.

Current guidelines highlight LDL-C as the primary therapeutic target for atherosclerotic cardiovascular disease (ASCVD) risk reduction, while TG serve as a management indicator in high-risk ASCVD patients who have achieved LDL-C goals (Mach et al., 2020; Wang Z. et al., 2023). Based on the findings of this NMA, lipid intervention targets, and Canadian Cardiovascular Society Angina Scale (CCS) grades (Chinese Society of Cardiology and Editorial Committee of Chinese Journal of Cardiology, 2000; Kaul et al., 2009), several potential treatment approaches can be considered: 1) For UAP patients (CCS ≤ II) with predominant LDL-C elevation or mixed hyperlipidemia, XZK + CT may be a potential option. 2) For UAP patients (CCS ≥ III) with predominant LDL-C elevation or mixed hyperlipidemia, MXK + CT may be worth exploring. 3) For UAP patients (CCS ≤ II) with hypertriglyceridemia, XZK + CT appears to be a viable choice. 4) For UAP patients (CCS ≥ III) with hypertriglyceridemia, YXTZ + CT or MXK + CT could be considered. However, these approaches should be further investigated in future clinical trials to determine their actual efficacy and safety.

#### 4.1.3 Other outcomes

In terms of improving PV, YXTZ + CT was ranked first according to the SUCRA value. Studies (Bao et al., 2013; Wang et al., 2013) show that GBE50 is able to improve the blood rheological indexes of rats with blood stasis syndrome, and compared with the normal and model groups, GBE50 significantly reduced whole blood viscosity, plasma viscosity, and erythrocyte aggregation index (P < 0.05), and the overall intervention effect is better than that of Ginaton, a representative drug of YXY.

In terms of improving hs-CRP, XST + CT was ranked first, while XZK + CT also demonstrated significant efficacy, securing third place in the cumulative ranking analysis. Meanwhile, XZK + CT was more effective in improving LDL-C levels and preventing MACEs compared with XST + CT. The reduction of LDL-C by XZK plays a role in preventing recurrent cardiovascular events, as supported by evidence linking aggressive LDL-C lowering to decreased MACE incidence (Vallejo-Vaz et al., 2018). Notably, studies have shown that despite standard treatment for acute coronary syndrome or other cardiovascular diseases, some patients remain in a persistent low-grade inflammatory state, indicating a potential risk for recurrent cardiovascular events, referred to as residual inflammatory risk (Klingenberg et al., 2021; Pradhan et al., 2018; Ridker, 2016). Although inflammatory markers (e.g., hs-CRP, IL-6, Interleukin-1β) remain elevated after treatment, indicating residual inflammation (Ridker et al., 2023); (Rothman et al., 2020). In addition to directly reducing hs-CRP levels, the statin component of XZK inhibits pro-inflammatory cytokines (e.g., IL-6, TNF-α) (Oesterle et al., 2017). Thus, the superior reduction in MACEs observed with XZK + CT may stem from its dual capacity to mitigate both residual cholesterol risk and residual inflammatory risk, whereas XST + CT, despite ranking first in hs-CRP improvement, demonstrates limited efficacy in addressing lipid-driven pathways—a distinction that underscores the potential advantage of comprehensive risk modulation.

However, the small sample size and limited MACE-focused studies may compromise result stability and generalizability. Future research should include larger, more diverse samples and well-designed RCTs, thereby providing more comprehensive evidence for the prevention and management of MACEs.

### 4.2 Efficacy of SSTCM-OPs: impact of drug source and preparation process

ZY, XST, and XY are pharmaceutical preparations derived from *P. ginseng C.A.Mey.*, *Panax notoginseng (Burkill) F.H.Chen*, and *Panax quinquefolius L.*, respectively, all belonging to the Araliaceae family in Table 1. A key distinguishing factor between ginseng species is the presence of specific ginsenosides (Kim, 2012). Ginsenoside Rf is found in both *P. ginseng C.A.Mey.* and *P. notoginseng (Burkill) F.H.Chen*, while pseudoginsenoside F11 is exclusive to *P. quinquefolius L.* (Choi, 2008). Notoginsenoside R1 is characteristic of both *P. ginseng C.A.Mey.* and *P. notoginseng (Burkill) F.H.Chen*. Additionally, the Rg1/Rb1 ratio is commonly used for differentiation, with values below 0.4 indicating *P. quinquefolius L.* and higher ratios typical of *P. ginseng C.A.Mey.* and *P. notoginseng (Burkill) F.H.Chen* (Yuan et al., 2010). While the efficacy of these three SSTCM-OPs treatments for patients with UAP is generally consistent, notable differences also exist. The observed results may be attributed to the shared profile of secondary metabolites, particularly ginsenosides, among these species, despite variations in their specific subtypes and concentrations (Kim, 2012; Yue et al., 2021). However, the precise mechanisms by which these secondary metabolites mediate cardiovascular effects remain elusive.

Importantly, studies (Liu et al., 2019; Xie and Wang, 2023) reveal that the bioactive saponins of XST, Panax notoginseng saponins (PNSs) show dual regulatory properties in balancing antiplatelet aggregation and pro-coagulant activities: First, PNSs interact synergistically with aspirin and clopidogrel by downregulating the platelet AA/COX/TXB2 pathway and suppressing clopidogrel resistance-related gene expression, thereby significantly enhancing their antiplatelet efficacy; Second, PNSs improve the outcomes of antithrombotic therapy by reducing the side effects associated with gastric mucosal damage. However, it is worth focusing on the possible side effects of consuming ginseng (including *P. ginseng C.A.Mey.* and *P. notoginseng (Burkill) F.H.Chen*) products in subjects taking cardiac, antidepressant, and anti-bleeding medications (Mancuso and Santangelo, 2017). Therefore, rigorous monitoring of bleeding risk and anxiety status is essential during the clinical administration of ZY and XY.

In addition, the performance of two *Ginkgo biloba* extracts (GBEs), YXY and YXTZ, in the treatment of unstable angina patients in this study revealed the influence of different botanical drug preparation processes on drug efficacy. The two main types of *Ginkgo biloba* oral preparations in China are: Yinxingye and Yinxingtongzhi oral preparations (Chinese Association of Integrative Medicine et al., 2020). Our research findings revealed that, with the exception of the effect on improving hs-CRP (MD = 1.94, CI: −0.70, 4.58), YXTZ demonstrated superiority over YXY in angina efficacy, ECG efficacy, nitroglycerin dosages, frequency of angina, duration of angina, and PV. The details are in Figure 4. The above results can be attributed to the fact that, in comparison with other *Ginkgo biloba* preparations, YXTZ has enhanced the content of pharmacologically active metabolites and reduced that of allergenic substances (Qian et al., 2021). Specifically, the quality control index of *Ginkgo biloba* oral preparations is higher than 24% of total flavonol glycosides and ginkgolide reaches more than 6%, while the requirement of Yinxingtongzhi oral preparation is stricter; its total flavonol glycosides content reaches more than 44%, and the main toxic metabolites of these two GBEs—ginkgolic acid is controlled below 5 μg/g (Cao et al., 2018; Liu et al., 2021). Their main adverse reactions caused by ginkgolic acid are digestive system symptoms (nausea, vomiting, diarrhea), headaches and dizziness, pallor, anxiety, weakness, or skin allergy (Biernacka et al., 2023; Shao et al., 2025). The combination of GBEs with aspirin, warfarin, and omeprazole may potentially induce drug-drug interactions, although the underlying mechanisms remain unclear (Mei et al., 2017). Furthermore, in patients taking antiplatelet or anticoagulant medications, the concurrent use of GBEs may elevate the risk of bleeding events (Biernacka et al., 2023; Mei et al., 2017).

### 4.3 Achievements and the path forward

Angina pectoris poses a substantial public health challenge, not only due to its association with elevated mortality risk but also through its detrimental effects on quality of life, including psychological distress and activity limitations. Within this context, SSTCM-OPs represent a complementary medical approach that enriches the therapeutic toolkit for angina management. By providing additional treatment options, SSTCM-OPs can be integrated with conventional medical approaches, potentially enhancing the overall management of patients (Hao et al., 2017). This integration may lead to better patient outcomes, such as improved quality of life, reduced reliance on certain medications, and enhanced cardiovascular function, thereby further validating the value and potential of complementary and alternative medicine in modern healthcare (Lin et al., 2024).

As modern medicine evolves and TCM research deepens, an increasing number of traditional Chinese medicine extracts have successfully obtained market approval after rigorous experimental and clinical studies, demonstrating the positive achievements of the modernization of TCM (Wu et al., 2023; Xiong et al., 2022). However, current clinical research of TCM in the field of cardiovascular diseases still has limitations (Dai et al., 2024). In the included primary studies, the lack of standardized terminology led to non-uniform reporting of adverse reactions. For example, some studies (Liu et al., 2013; Peng et al., 2020) grouped “headache” and “dizziness” into a combined term, while others reported them as distinct symptoms. Moreover, only a small number of studies have provided standardized reports on allergic reactions, dose-dependent responses, liver function, renal function, and other relevant factors. This is a matter that requires our vigilance. Standardized safety reporting is critical in treatment, directly influencing clinicians’ medication choices, thereby necessitating the establishment of a standardized safety reporting system in TCM and the integration of comprehensive safety metrics in future research.

At present, no objective and systematic framework exists to elucidate TCM mechanisms in the prevention and treatment of CHD (Wang and He, 2010). Integrated metabolomics offers a more comprehensive approach to understanding the effects of TCM and could facilitate the establishment of such a system (Wu et al., 2019). Therefore, additional high-quality clinical studies are essential to enhance the evaluation framework and refine its components. Meanwhile, current traditional cell culture and animal models have limitations in reflecting human physiology, leading to significant discrepancies in results. Cardiac organ chips are increasingly gaining attention, providing substantial benefits in disease modeling, drug screening, personalized medicine, and education and training (Li et al., 2024). We suggest integrating this technology into modern TCM research frameworks to evaluate multi-component synergistic effects and potential toxicity, thereby addressing critical gaps in TCM safety and efficacy assessment.

Publication bias is a critical concern in meta-analyses, as it may distort the interpretation of intervention effects by favoring the publication of statistically significant or positive results, particularly small-study effect. The observed bias may have implications for clinical decision-making. For instance, LDL-C is a key biomarker for cardiovascular risk assessment, and biased estimates of treatment effects on these outcomes may mislead clinical guideline recommendations. In contrast, symmetric funnel plots suggested low risk of small-study effects. Nevertheless, publication bias cannot be entirely ruled out, and the current evidence derived exclusively from Chinese RCTs may limit the generalizability of findings to other populations, particularly given region-specific variations in clinical practice. Given these limitations, clinicians and policymakers should interpret results for frequency of angina, nitroglycerin dosages, TC, and LDL-C with particular caution. In this study, the treatment course was a significant source of heterogeneity. Moreover, concerns regarding methodological limitations, particularly the inadequate documentation of allocation concealment and blinding implementation, persist as prevalent issues in traditional Chinese medicine RCTs. These methodological flaws also contribute substantially to the observed heterogeneity. Based on methodological considerations, we propose the following recommendations: 1) Future meta-analyses would benefit from including unpublished data or gray literature to mitigate bias. 2) Large-scale, rigorously designed trials are needed to verify these findings and enhance the quality of evidence, especially for outcomes with heterogeneous methodologies. 3) Future studies should incorporate international clinical trials to validate external validity and minimize confounding from cultural or medical practice disparities.

Additionally, by conducting in-depth research on the clinical efficacy of specific TCM preparations in terms of key indicators such as hs-CRP, PV, and MACEs, we comprehensively and systematically analyzed the action mechanisms and values of TCM preparations in the treatment of cardiovascular diseases. The research results strongly confirm the significant advantages of TCM in improving cardiovascular risks, optimizing inflammation monitoring, and enhancing treatment effects. Meanwhile, the findings of this study also provide a basis for the selection of TCM preparations with multiple therapeutic advantages in clinical practice. These findings are expected to promote the standardization and precision of TCM treatment for CHD, opening up new avenues for the clinical treatment of cardiovascular diseases.

### 4.4 Limitations

There are some limitations of this study. 1) All the included RCTs were in China. 2) The quality of the included RCTs needs to be improved, half of the 72 included RCTs did not mention the method of random sequence generation, only 3 RCTs mentioned allocation concealment, 4 RCTs mentioned blinding of participants and staff, and none of the studies described blinding of outcome assessment, all the above may affect the reliability of the overall findings. 3) Clinical heterogeneity existed due to differences in dosage and duration of treatment for different interventions. 4) MACEs are important outcome indicators for UAP, and there were fewer studies on this. 5) Most studies primarily assessed adverse reactions based on symptoms, with inconsistent reporting standards; Additionally, there was a lack of reporting on safety indicators. 6) The quality of evidence is also negatively affected by the fact that this NMA did not constitute a closed loop.

## 5 Conclusion

In conclusion, SSTCM-OPs combined with CT showed advantages over CT alone in treating UAP. Among them, MXK + CT demonstrates excellent clinical efficacy in the management of UAP. Additionally, XZK, YXTZ, and MXK combined with CT may improve lipid levels and contribute to secondary prevention in unstable angina pectoris patients. Meanwhile, XZK + CT is more effective in improving LDL-C levels and preventing MACEs compared with XST + CT, establishing it as a suitable option for preventing cardiovascular risk events. These findings underscore the need for personalized treatment strategies based on individual patient characteristics, which may substantially improve clinical efficacy and mitigate the disease burden in UAP patients.

## Data Availability

The original contributions presented in the study are included in the article/Supplementary Material, further inquiries can be directed to the corresponding authors.

## Glossary

ACS: Acute coronary syndrome
ASCVD: Atherosclerotic cardiovascular disease
C: Control group
CAM: Complementary and alternative medicine
CCS: Canadian Cardiovascular Society Angina Scale
CHD: Coronary heart disease
CI: 95% confidence intervals
CNKI: China National Knowledge Infrastructure
CT: Conventional treatments
DAXXK: Diaoxinxuekang oral preparation
DZHJT: Dazhuhongjingtian oral preparation
ECG: Electrocardiogram
F: Female
GBE50: *Ginkgo biloba* extract 50
GBEs: *Ginkgo biloba* extracts
GSTT: Gross Saponins of *Tribulus terrestris L.*
HDL-C: High-density Lipoprotein Cholesterol
HMG-CoA: Hydroxymethylglutaryl-coenzyme A
hs-CRP: High-sensitivity C-reactive Protein
LDL-C: Low-density lipoprotein cholesterol
M: Male
MACEs: Major adverse cardiovascular events
MD: Mean difference
MXK: Maixuekang oral preparation
NA: Not available
NMA: Network meta-analysis
SSTCM-OPs: Oral preparations of single-source traditional Chinese medicines
PNSs: Panax notoginseng saponins
PRISMA: Preferred Reporting Items for Systematic Reviews and Meta-Analyses
PV: Plasma viscosity
RCTs: Randomized controlled trials
RR: Relative Risk
CBM: China Biomedical Literature Database
SMD: Standardized mean difference
SNOSE: sequentially numbered, opaque sealed envelopes
SUCRA: Surface under the cumulative ranking curve
T: Treatment group
TC: Total cholesterol
TCM: Traditional Chinese medicine
TG: Triglyceride
UAP: Unstable angina pectoris
VIP: China Science and Technology Journal Database
XDK: Xindakang oral preparation
XNST: Xinnaoshutong oral preparation
XST: Xuesaitong oral preparation
XY: Xinyue oral preparation
XZK: Xuezhikang oral preparation
YXTZ: Yinxingtongzhi oral preparation
YXY: Yinxingye oral preparation
ZY: Zhenyuan oral preparation
*β*: Regression coefficient
