# Supplementary material for: Efficacy and safety of 11 oral preparations of single-source traditional Chinese medicines in the treatment of unstable angina pectoris: a systematic review and network meta-analysis
Source: Front Pharmacol. 2025 Jun 24;16:1582661. doi: 10.3389/fphar.2025.1582661 (PMC12235920; doi:10.3389/fphar.2025.1582661)
Supplement: Supplementary file 1 [file Supplementaryfile1.docx]

**Supplement material 1. Chemical metabolites of oral preparations of single-source traditional Chinese medicines**

**1. data from liquid chromatography in literature**

**1.1 Determination of the content of** **Zhenyuan oral preparation (Wu et al., 2007)**

1.1.1 Source of drugs

Zhenyuan Capsules 3 batches (Jilin Ji'an Yisheng Pharmaceutical Co., Ltd, batch nos. 031118, 040329, 050109).

1.1.2 Preparation of test solution

Take about 0.5g of the contents of the product, precision weighing, precision addition of water-saturated n-butanol 20mL, ultrasonic treatment for 20min, filtration, discard the initial filtrate, take the filtrate 10mL, washed with ammonia test solution 10mL. The n-butanol solution was concentrated to dryness under reduced pressure, and the residue was dissolved with methanol, transferred to a 10 mL measuring flask, and the solution was calibrated with methanol, shaken well, and then filtered through microporous membrane (Φ0.45 μm), and the filtrate was taken as the filtrate, which was obtained.

1.1.3 Chromatographic conditions and chromatogram

The chromatographic analysis was performed on a LichrospherC18 column (4.6 mm×250 mm, 3.5μm) with acetonitrile-0.05% phosphoric acid (21:79) as mobile phase at a flow rate of 1 mL-min-1 at a detection wavelength of 203 nm, and a sample volume of 5μL at a flow rate of 1 mL-min-1 and a sample volume of 35 ℃. The retention time of the peaks of ginsenoside Re was 39.8 min, and the separation of ginsenoside Re was more than 1.5, and the theoretical plate number was more than 14000. The chromatogram is shown in Figure 1.


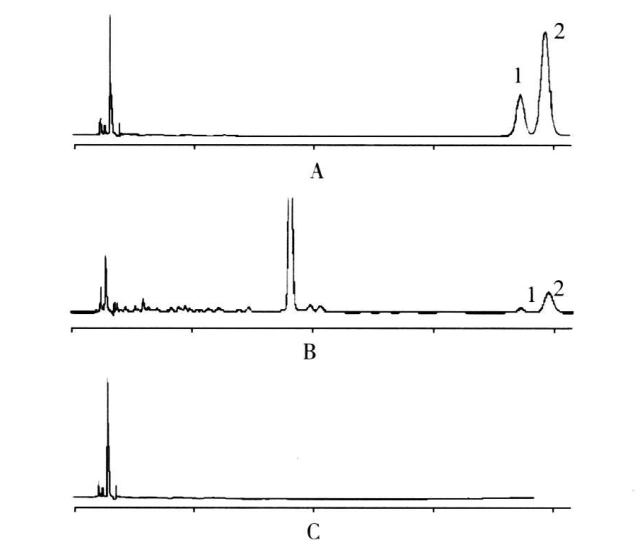


Fig.1 HPLC chromatograms of ginsenoside Rg1 and ginsenoside Re.

(A)reference substance. (B)sample of Zhenyuan capsules. (C)excipients. 1.ginsenoside Rg1; 2. ginsenoside Re.

1.1.4 Sample determination

Table1 Quantitative analysis results of Zhenyuan capsules

| Batch number | Ginsenoside Re(mg/capsule ) | Relative standard deviation; RSD(%) |
| --- | --- | --- |
| 031118 | 1.8487 | 1.82 |
| 040329 | 1.5747 | 2.03 |
| 050109 | 2.7800 | 1.80 |

**1.2 Determination of the content of Xuesaitong oral preparation (Han et al., 2015; Li et al., 2014)**

1.2.1 Source of drugs

3 batches of Xuesaitong capsules (Yunnan Peacekeeping Pharmaceutical Co., Ltd, 100mg, batch nos. 111023, 120917, 130329).

Xuesaitong Soft Capsules (Kunming Pharmaceutical Group Co., Ltd, Specification: 100 mg, Batch No. 130901-08, 131111-02, 131111-03).

1.2.2 Preparation of test solution

Take the content under the loading difference of Xuesaitong capsules, take about 60 mg, weigh it precisely, put it in a 10 mL measuring flask, add methanol, ultrasonic for 20 min, take it out and let it cool down, then add methanol to the scale, filtrate, and then the filtrate is used as the solution for the test product.

Take 2 Xuesaitong soft capsules, take out the contents and mix them evenly, about 180 mg, put it in 25 mL measuring flask, add methanol, ultrasonic treatment for 5 min, take it out and put it to room temperature, add methanol to dilute it and set it to the scale, shake it well, and then filter it through 0.45 μm micropore membrane, that is, it will be the solution for the test material, and then keep it in a low temperature for spare parts.

Chromatographic conditions and chromatogram

1.2.3 Chromatographic conditions and chromatogram

Column: Agilent Extend-C 18 column (4.6 mm×250 mm, 5 μm), WATCH pre-column. Mobile phase: acetonitrile as mobile phase A, water as mobile phase B, gradient elution according to Table 2a column temperature 30 ℃, detection wavelength: 203 nm, injection volume: 20 μL, flow rate: 1.0 mL/min, the separation between the peaks of ginsenoside Rg 1 and ginsenoside Re was not less than 1.5. The theoretical plate number should be not less than 6000 according to the ginsenoside Rg 1 calculation. The chromatograms of the three solutions are shown in Figure 2a-2c, respectively.

Tab.2a Gradient elution program of mobile phase

| Time (min) | A (100%) | B (100%) |
| --- | --- | --- |
| 0~30 | 19 | 81 |
| 30~35 | 19→45 | 81→55 |

The separation was carried out on a ZORBAX SB-Aq column (250 mm×4.6 mm, 5 μm) with the mobile phase of acetonitrile-water and the gradient elution according to the method shown in Table 2b; Mobile phase: acetonitrile as mobile phase A, water as mobile phase B. The injection volume was 5 μL; the flow rate was 1.0 mL-min -1; the detection wavelength was 203 nm; and the temperature of the column was 25 ℃.

Tab.2b Table of gradient elution

| Time (min) | A(100%) | B(100%) |
| --- | --- | --- |
| 0 | 19 | 81 |
| 35 | 19 | 81 |
| 40 | 29 | 71 |
| 60 | 29 | 71 |
| 70 | 36 | 64 |
| 75 | 19 | 81 |

Fig.2a Chromatogram of reference sample. Fig.2b Chromatogram of experiment sample. Fig.2c Chromatogram of negative sample.


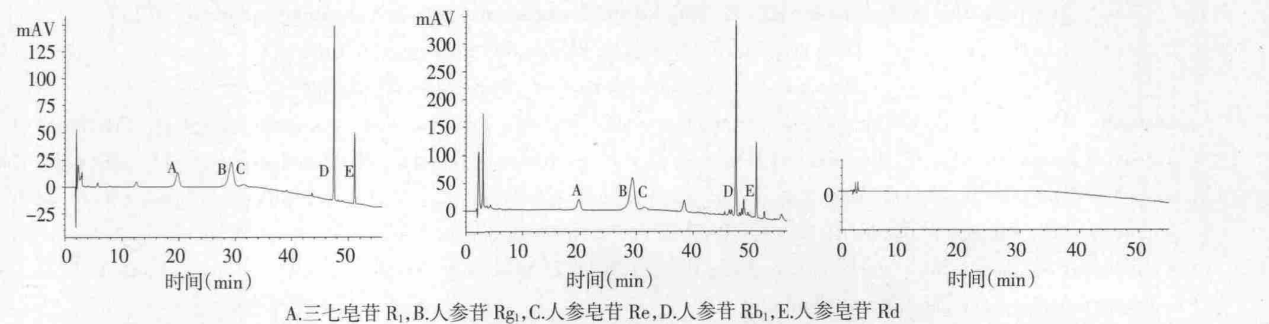


(A)Notoginsenoside R1; (B)Ginsenoside Rg1; (C)Ginsenoside Re; (D)Ginsenoside Rb1. (E)Ginsenoside Rd.

1.2.4 Sample determination

Tab.2c Determination results of Xuesaitong capsules sample content

(X±S) mg/capsule

| Batch number | Notoginsenoside R1 | Ginsenoside Rg1 | Ginsenoside Re | Ginsenoside Rb1 | Ginsenoside Rd | Total saponin |
| --- | --- | --- | --- | --- | --- | --- |
| 111023 | 9.00±0.15 | 42.84±0.18 | 3.67±0.11 | 34.21±0.60 | 8.32±0.10 | 98.05±1.12 |
| 120917 | 8.97±0.19 | 41.60±0.89 | 3.43±0.07 | 34.43±0.75 | 7.82±0.12 | 96.26±2.00 |
| 130329 | 10.06±0.14 | 45.90±0.85 | 3.68±0.05 | 35.72±0.39 | 8.29±0.09 | 103.64±2.16 |

Tab.2d Content determination of Xuesaitong soft capsules sample Content/%

| Batch number | Notoginsenoside R1(%) | Ginsenoside Rg1(%) | Ginsenoside Re(%) | Ginsenoside Rb1(%) | Total saponin(%) |
| --- | --- | --- | --- | --- | --- |
| 130901-08 | 13.875 | 30.875 | 4.750 | 27.500 | 77.000 |
| 131111-02 | 11.750 | 31.125 | 5.125 | 28.625 | 76.625 |
| 131111-03 | 12.125 | 32.250 | 5.125 | 29.625 | 79.125 |

**1.3 Determination of the content of Xinyue oral preparation (Zhao et al., 2024)**

1.3.1 Source of drugs

The samples of Xinyue capsules were purchased from a pharmacy (batch numbers 2107591, 180601, 180604, Jilin Jian Yisheng Pharmaceutical Co.)

1.3.2 Preparation of test solution

Take the contents of 10 capsules of this product (average mass of 0.3 mg per capsule), research fine, take about 0.25 g, precision weighing, put in a stoppered conical flask, precision adding 80% methanol 25 mL, tightly stoppered, weighed the quality of the product, put in a water bath and heat the reflux for 75 min, take out, let cool, and then weighed the quality of the product, 80% methanol to make up for the loss of mass, shaking, filtration, precision measurement of 1 mL of filtrate, put in a 100 mL flask, and then put in a 100 mL flask. 100 mL measuring flask, add 20% acetonitrile aqueous solution dilution and volume to the scale, shake well, filtered, that is obtained.

1.3.3 Chromatographic conditions

The separation was performed on a Waters BEH C18 column (100 mm×2.1 mm, 1.7 μm) with a gradient elution of acetonitrile (A)-water (B) as the mobile phase (0~2.00 min, 20%~50%A; 2.00~2.01 min, 50%~80%A; 2.01~3.00 min, 80%A) at a temperature of 40 ℃ and a flow rate of 0.5 mL/min-1. The flow rate was 0.5 mL/min-1; injection volume was 5 μL.

1.3.4 Sample determination

The established method was used to determine the contents of six saponin-like metabolites in three batches of Xinyue capsule samples, and the results are shown in Table 3.

Table 3 Determination of the content of 6 saponin metabolites in Heart's Delight Capsules (mg/capsule)

| Batch number | Ginsenoside Rg1 | Ginsenoside Re | Ginsenoside Rb1 | Ginsenoside F11 | Ginsenoside Rd | 20(S)-Ginsenoside F1 | Total saponin |
| --- | --- | --- | --- | --- | --- | --- | --- |
| 2107591 | 0.78 | 4.41 | 0.56 | 4.13 | 4.74 | 0.13 | 14.75 |
| 180601 | 1.02 | 4.53 | 0.68 | 4.38 | 5.02 | 0.15 | 15.78 |
| 180604 | 0.89 | 4.84 | 0.62 | 4.56 | 5.14 | 0.15 | 16.20 |

**1.4 Determination of the content of Diaoxinxuekang oral preparation (Zhang et al., 2015)**

1.4.1 Source of drugs

The samples of Diaoxinxuekang capsules were commercially available, batch numbers: 0702035, 0703007, 0704017, 0704060, 0704069 from Chengdu Dior Pharmaceutical Group Co.

1.4.2 Preparation of test solution

Weigh 0.2g of the content of Dior Cardiovascular Capsules, weigh it precisely, put it in a stoppered triangular flask, add 10 times the amount of methanol and ultrasonic treatment (power: 120 W, ultrasonic frequency: 40 KHz) for 30 min, remove it, filter it, evaporate the solvent under reduced pressure, add methanol to the residue, dissolve it and condense it into a 50 mL flask, shake it well and then pass it through 0.45 μm microporous filtration membrane, then get the solution of the test material.

1.4.3 Chromatographic conditions and chromatogram

Column: Shim-pack VP-ODS column (250 mm × 4.6 mm, 5 μm); mobile phase: acetonitrile (A) - water (B) (0-15 min, B: 75% → 71%; 15-25 min, B: 71%; 25-45 min, B: 71% → 65%; 45-65 min, B: 65% → 40%; 66-66 min, B: 65% → 40%). B: 71% → 65%; 45-65 min, B: 65% → 40%; 66 min, B: 30%; 66-75 min, B: 30% → 0%); flow rate: 0.8 mL-min-1; gas (air) flow rate: 2.7 L-min-1; nebulization temperature: 105 ℃; Column temperature: 35 ℃; injection volume: 10 μL.

Under the chromatographic conditions, the separation between the chromatographic peaks of protodioscin, methyl protodioscin and diosgenin and the adjacent peaks was more than 1.5, and the theoretical plate number was not less than 20,000 according to the calculation of protodioscin. The chromatograms of the negative sample, control and samples are shown in Figure 4.

Fig 4. The chromatograms of the negative sample, control and samples


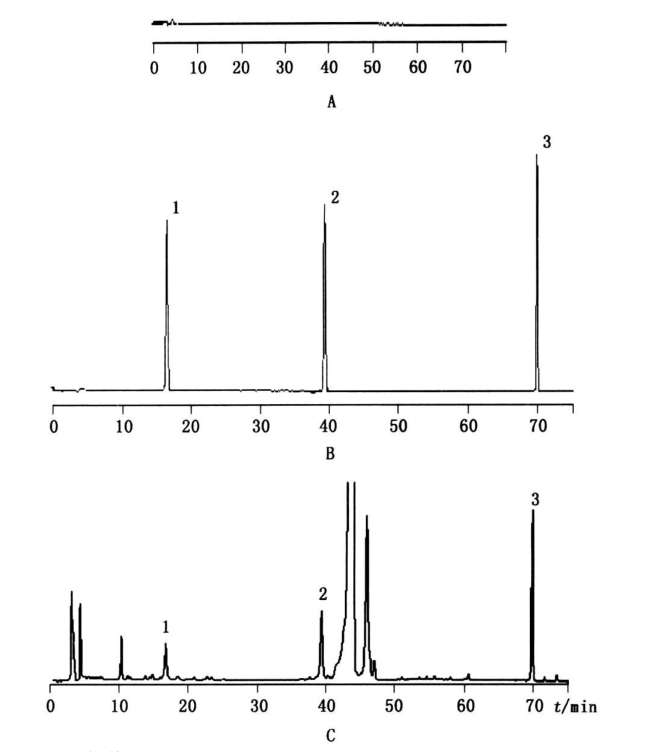


(A)Negative sample without Dioscorea Panthaica et Burkill and Dioscorea niponica makino. (B)Chemical reference substances. (C)sample. 1.Protodioscin. 2.Methylprotodios-cin. 3.Dioscin.

1.4.4 Sample determination

Content of different compounds for 5 batches Diaoxinxuekang capsules

(mg per capsule, n=3)

| Batch number | Potodioscin | Mthylprotodioscin | Doscin |
| --- | --- | --- | --- |
| 0702035 | 5.98(1.0) | 6.47(1.4) | 36.53(0.7) |
| 0703007 | 6.77(1.3) | 7.87(1.5) | 34.29(1.1) |
| 0704017 | 5.69(1.7) | 8.40(2.0) | 37.78(1.5) |
| 0704060 | 7.02(1.3) | 7.57(0.8) | 39.49(2.1) |
| 0704069 | 6.90(2.0) | 8.63(1.1) | 39.56(1.9) |

Note：RSD(%) in the parenthesis

**1.5 Determination of the content of Xuezhikang oral preparation (Li and Sun, 2019)**

1.5.1 Source of drugs

8 batches of Xuezhikang capsules (batch numbers: 170324, 170420, 170608, 170903, 171209, 171222, 180422, 180603 from Peking University Weixin Biotechnology Co., Ltd.)

1.5.2 Preparation of test solution

Take 10 Xuezhikang capsules , remove the capsule shell, pour out the contents, mix and crush, pass through a 60-mesh sieve; weigh the capsule powder about 0.5 g, put it in a 50 mL stopper conical flask, add 15 mL of methanol, weigh the mass, ultrasonicate the capsule (power: 250 W, frequency: 40 kHz) for 30 min, let it cool down, weigh the mass again, and make up for the mass loss with methanol, then centrifuge the capsule for 10 min in 13 000 r/min and take 10 μL of the supernatant into the sample for analysis. After centrifugation at 13 000 r/min for 10 min, 10 μL of the supernatant is taken and analyzed.

1.5.3 Chromatographic conditions and chromatogram

Column: Shimadzu Hypersil Gold C 18 (250 mm × 4.6 mm, 5 µm); mobile phase: 0.1% formic acid aqueous solution (A) - acetonitrile (B), gradient elution (0-3 min, 5% B → 10% B; 3-8 min, 10% B → 20% B; 8-20 min, 20% B → 34% B; 20-28 min, 34% B → 34% B). (0-3 min, 5% B→10% B; 3-8 min, 10% B→20% B; 8-20 min, 20% B→34% B; 20-28 min, 34% B→43% B; 28-40 min, 43% B→65% B; 40-52 min, 65% B→90% B; 52-55 min, 90% B→100% B); Flow rate: 1.0 mL/min; Detection wavelength: 237 nm (lovastatin, lovastatin hydroxy acid, and mevastatin), 256 nm (soy glucoside, soy glycoside, flavonoid, and dye). soybean glycosides, soybean flavonoids, genistein and genistein); column temperature: 30 ℃; injection volume: 10 μL. The high performance liquid (HPLC) chromatogram is shown in Figure 5.

Fig. 5 HPLC chromatograms


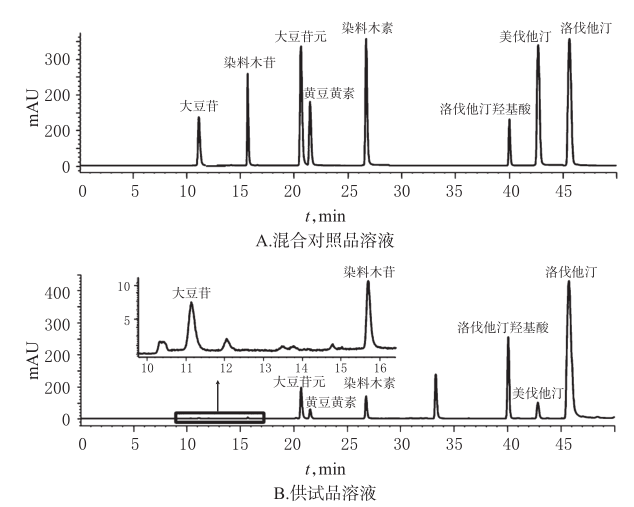


A. Mixed control solution. B.Test solution. 大豆苷:daidzin, 染料木苷:genistin, 大豆苷元:daidzein, 黄豆黄素:glycitein, 染料木素:genistein, 洛伐他汀羟基酸:lovastatin hydroxy acid, 美伐他汀:mevastatin, 洛伐他汀:lovastatin.

1.5.4 Sample determination

The samples of 8 batches of Xuezhikang Capsules were taken in appropriate quantities and determined in parallel for 3 times, the peak area of each metabolite was recorded, and the content of each metabolite in the samples was calculated by the external standard method. As a result, the content of lovastatin was the highest in the 8 batches of Lipocere capsule samples, but the content of statin metabolites varied greatly, especially lovastatin and lovastatin hydroxy acid; the content of soybean glycosides was higher in the isoflavone metabolites. The results of sample content determination are shown in Table 5.

Table 5 Results of content determination of samples

(mg/g, n=3)

| Batch number | Daidzin | Genistin | Daidzein | Glycitein | Genistein | Lovastatin hydroxy acid | Mevastatin | Lovastatin |
| --- | --- | --- | --- | --- | --- | --- | --- | --- |
| 170324 | 0.0155 | 0.0242 | 0.3383 | 0.0589 | 0.2261 | 1.9834 | 0.0448 | 14.3646 |
| 170420 | 0.0142 | 0.0223 | 0.2998 | 0.0570 | 0.2298 | 3.6758 | 0.0909 | 15.2013 |
| 170608 | 0.0123 | 0.0169 | 0.3342 | 0.0576 | 0.2386 | 3.9252 | 0.0861 | 13.2944 |
| 170903 | 0.0179 | 0.0279 | 0.3668 | 0.0606 | 0.2938 | 3.0583 | 0.0782 | 10.6285 |
| 171209 | 0.0147 | 0.023 1 | 0.4009 | 0.0691 | 0.2789 | 3.3536 | 0.0849 | 13.2088 |
| 171222 | 0.0163 | 0.025 7 | 0.3670 | 0.0543 | 0.2453 | 2.5216 | 0.0506 | 11.5793 |
| 180422 | 0.0162 | 0.0268 | 0.3614 | 0.0509 | 0.2451 | 2.7458 | 0.0537 | 10.9869 |
| 180603 | 0.0155 | 0.025 4 | 0.3791 | 0.0586 | 0.2357 | 2.7238 | 0.0591 | 15.8724 |

**1.6 Determination of the content of Yinxingye oral preparation (Bai et al., 2019)**

1.6.1 Source of drugs


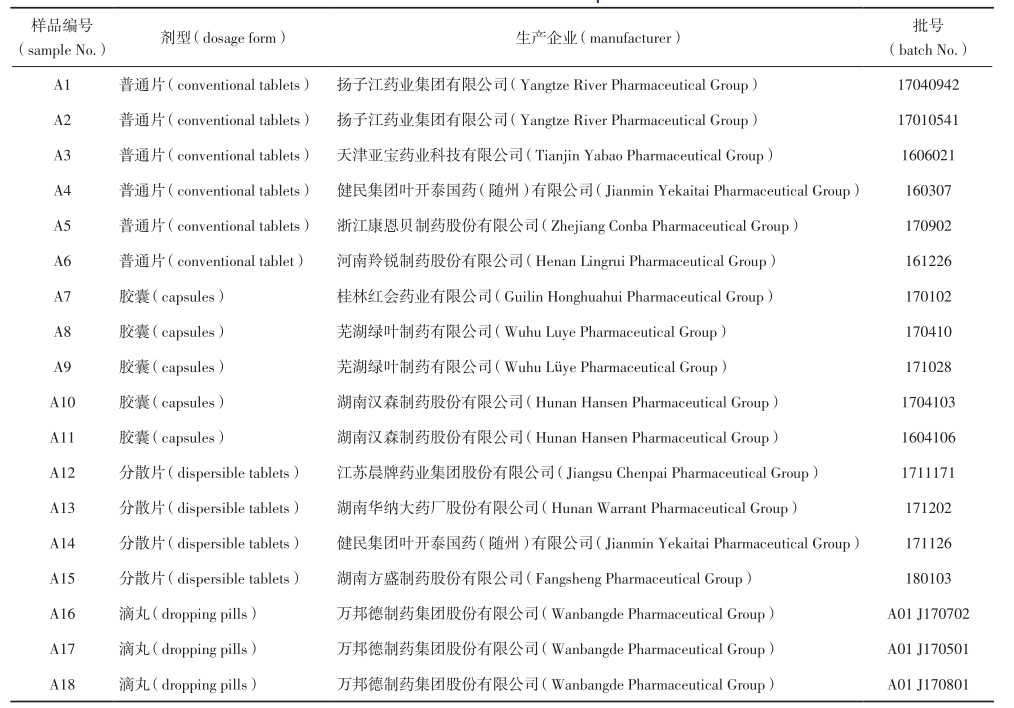


1.6.2 Preparation of test solution

Take 20 tablets (granules), remove the coating (or take the contents), weigh precisely, and grind finely; take the powder equivalent to 19.2 mg of terpene lactone, weigh precisely, put it in a stoppered bottle, add 50 mL of methanol precisely, stopper tightly, weigh, ultrasonicate (250 W, 33 kHz) for 20 min, let it cool down, weigh it again, and make up for the lost amount with methanol, and then filter it by shaking well. Precision measurement of the filtrate 20 mL, evaporated, residue with 10 mL of water, placed in a water bath to warm the dissolution, add 2% hydrochloric acid solution 2 drops, ethyl acetate shaking extraction 4 times (15, 10, 10, 10 mL), combined with the extract, with 5% sodium acetate solution 20 mL wash containers, 10 mL of ethyl acetate to wash the sodium acetate solution, with ethyl acetate extract and washings, and then washed with water. Wash the container with 20 mL of 5% sodium acetate solution, wash the sodium acetate solution with 10 mL of ethyl acetate, combine with the ethyl acetate extract and washing solution, then wash with water for 2 times, 20 mL each time, combine with the aqueous solution, wash with 10 mL of ethyl acetate, combine with the ethyl acetate solution, evaporate to dryness, and then dissolve the residue with methanol and transfer it to 20 mL volumetric flask, add methanol to the scale, and then shake well, then obtain. It was filtered with 0.45 μm microporous membrane, and was obtained.

1.6.3 Chromatographic conditions and chromatogram

The separation was performed on a Poroshell 120 SB-C 18 (150 mm×4.6 mm, 2.7 μm) with methanol (A) and water (B) as mobile phases in a binary gradient elution (0-6 min, 25%A → 28%A; 6-28 min, 28%A → 30%A) at a flow rate of 0.8 mg-mL-1, column temperature of 35 ℃ and an injection volume of 5 μL. CAD, carrier gas N 2, pressure 438.5 kPa, nebulization temperature 35 ℃, acquisition frequency 10 Hz, filter 3.6. The chromatogram is shown in Figure 6.

Fig. 6 Chromatograms of total reference extract of ginkgo leaves(A) and Ginkgo leaf tablet A1(B)


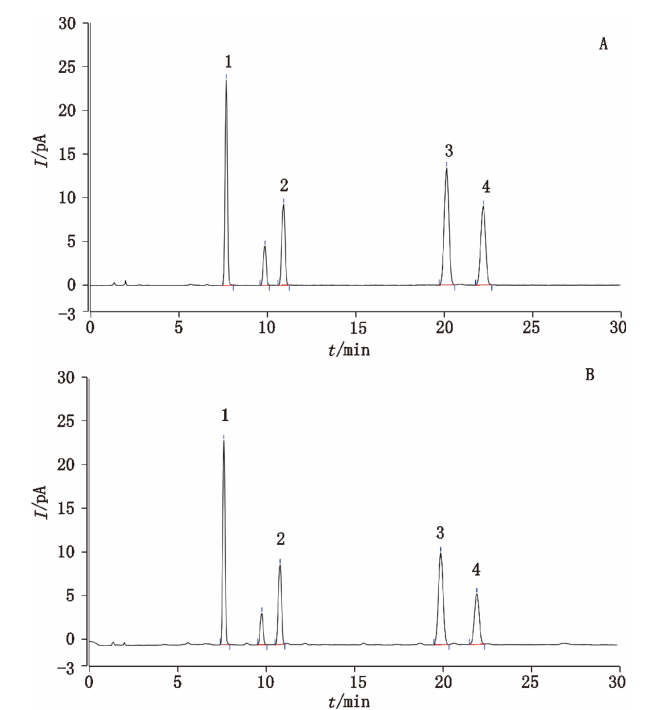


1. bilobalide, 2. ginkgolide C, 3. ginkgolide A, 4. ginkgolide B.

1.6.4 Sample determination

A total of 18 batches of Ginkgo biloba preparations in 4 dosage forms were taken, and the results of the determination of bilobalide, ginkgolide A, ginkgolide B and ginkgolide C in the samples of different dosage forms of Ginkgo biloba preparations are shown in Table 6.

Table 6 Determination of the content of terpene lactones in 18 batches of samples (mg per tablet or capsule or pill)


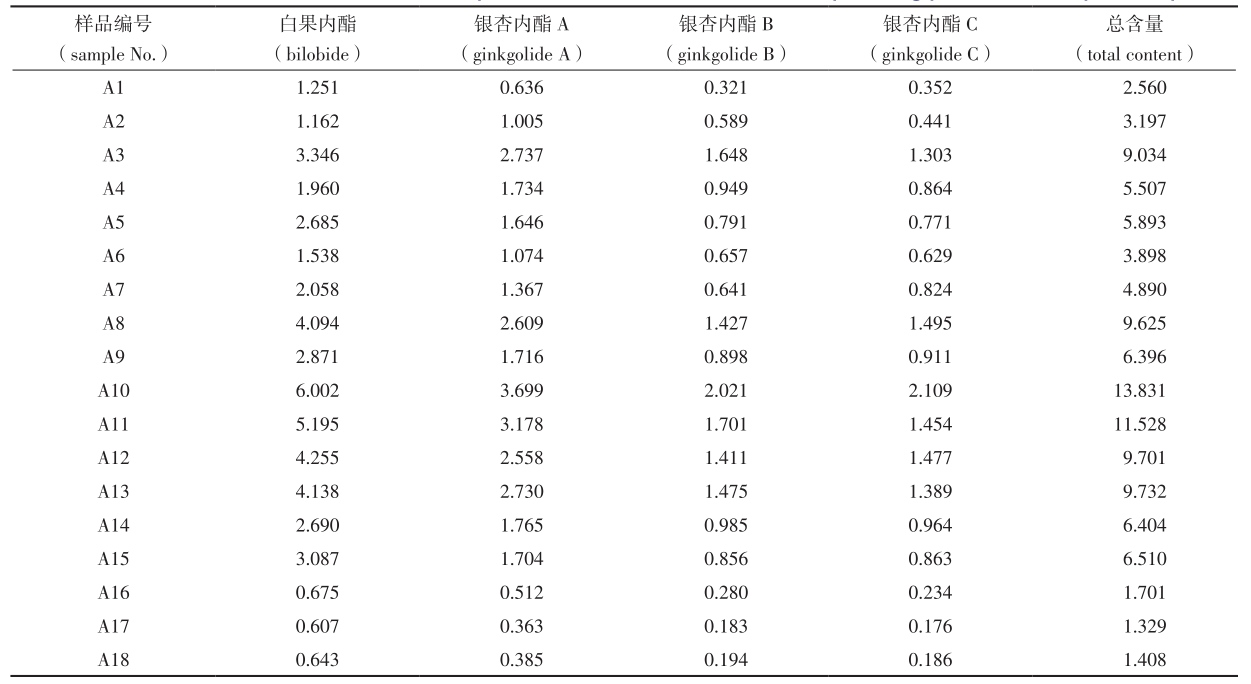


**1.7 Determination of the content of Yinxingtongzhi oral preparation (Li et al., 2007; Zhou et al., 2010)**

1.7.1 Source of drugs

Ginkgo Biloba Ketone Ester Drops is offered by Shanxi Qianhui Pharmaceutical Co.

Ginkgolides dispersible tablets are products of Jiangsu Plant A. Specification: Batch No. 20090605, each tablet contains 40 mg of ginkgolides.

1.7.2 Preparation of test solution

Take ginkgo biloba ketone ester drops appropriate amount, research fine, take the equivalent of terpene lactone 19.2mg of fine powder, precision weighing, precision add methanol 50mL, weighing, ultrasonic treatment (50W, 50kHz, the beginning of the temperature of the bath 25 ° C) for 20min, cool, and then weighing, methanol to make up for the loss of weight, shaking, filtration, precision measurement of the filtrate 20mL, recovery of methanol, residue add water 10mL, placed in a water bath to warm to dissolve, add 2% hydrochloric acid solution 2 drops, ethyl acetate extraction with oscillation 4 times (15,10,10,10mL), combined extract, with 5% sodium acetate solution 20mL wash, split sodium acetate solution, and then wash with ethyl acetate 10mL. Combined ethyl acetate extract and wash solution, washed with water 2 times, 20mL each time, combined with the water wash solution, washed with ethyl acetate 10mL, combined with the ethyl acetate solution, recovery of ethyl acetate to dryness, the residue was dissolved in acetone and transferred to a 5mL measuring flask, add acetone to the scale, shaking, and then filtered through microporous membrane (0.45μm), the filtrate was extracted, that is, the product was obtained.

Take 10 tablets, fine, precision weighing equivalent to the weight of 1 tablet, placed in a 100 mL measuring flask, add 0.1 mol/L hydrochloric acid solution to the scale, ultrasonic extraction for 30 min, take the solution of 7 mL, filtered, take the solution of 7 mL, filtration, precision aspiration of 5 mL, extracted by shaking with methyl acetate for 2 times, 10 mL each time, combined with methyl acetate solution, evaporation, residue add methanol 1 mL to make the dissolution of placed in a 2 mL flask. 2 mL volumetric flask, dilute with mobile phase to the scale, shake well, filtered, that is obtained.

1.7.3 Chromatographic conditions and chromatogram

Column: Angilent Hypersil ODS (5μm, 4mm×250mm); mobile phase: tetrahydrofuran-methanol-water (10∶20∶70); flow rate: 1.0mL/min; column temperature: 35℃; temperature of the drift tube: 103℃; nitrogen flow rate: 2.51L/min; the theoretical plate count should be less than 2500 according to the peak of albiglactone. The theoretical plate number should not be less than 2500 according to the white fruit lactone peak, and the separation between the white fruit lactone peak and the ginkgolide C peak should be more than 1.5. Record the chromatogram (see Figure 7a).

Figure 7a Sample solution HPLC chromatogram


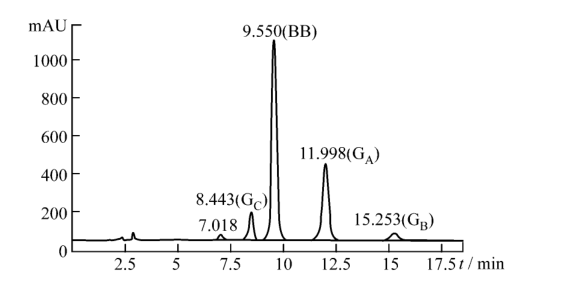


G_C_:ginkgolide C BB:bilobalide G_A_:ginkgolide A G_B_:ginkgolide B

Alltech C 18 column (4.6 mm × 150 mm, 5 μm), mobile phase: water-acetonitrile-tetrahydrofuran (80 ∶ 8 ∶ 12); flow rate: 1.0 mL/min; drift tube temperature: 105 ℃, nitrogen flow rate: 2.70 mL/min. Theoretical plate counts were calculated according to the peaks of ginkgolide A, ginkgolide B, ginkgolide C and bilobalide. The theoretical plate counts of ginkgolide A, ginkgolide B, ginkgolide C and bilobalide peaks were not less than 3 000. Record the chromatogram (see Figure 7b).

Fig. 7b HPLC Chromatogram


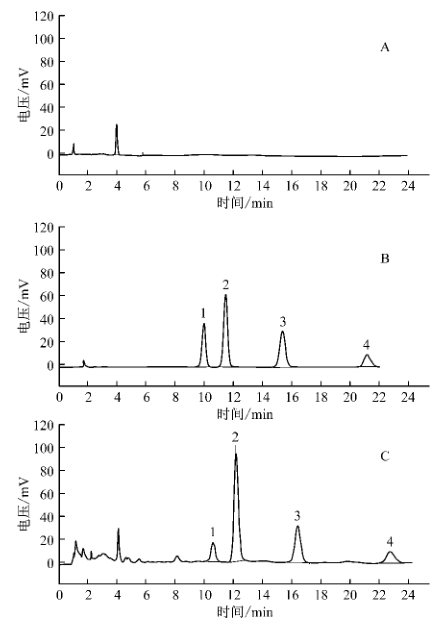


1. ginkgolide C 2. bilobalide 3. ginkgolide A 4. ginkgolide B (A. Negative samples; B. Mixed controls; C. Tests)

1.7.4 Sample determination

The contents of bilobalide, ginkgolide A, ginkgolide B and ginkgolide C in ginkgo biloba ketone ester drops were calculated, and the results are shown in Table 7.

Table 7 Sample content determination results

| Batch number | bilobalide | ginkgolide A | ginkgolide B | ginkgolide C | total lactone |
| --- | --- | --- | --- | --- | --- |
| 20030921 | 0.150 | 0.095 | 0.021 | 0.043 | 0.309 |
| 20030922 | 0.141 | 0.090 | 0.020 | 0.041 | 0.292 |
| 20030923 | 0.154 | 0.095 | 0.021 | 0.043 | 0.313 |
| 20030924 | 0.147 | 0.091 | 0.020 | 0.041 | 0.299 |
| 20030925 | 0.162 | 0.101 | 0.022 | 0.045 | 0.330 |
| 20030926 | 0.165 | 0.103 | 0.023 | 0.047 | 0.338 |

About 75 mg of a sample with known content (Plant A, Lot No. 090407, containing ginkgolides 5. 761 mg/tablet, including bilobalide 2. 578 mg/tablet, ginkgolide A 1. 546 mg/tablet, ginkgolide B 0. 987 mg/tablet, ginkgolide C 0. 65 mg/tablet) was weighed in 5 portions, and added to the mixed control solution (1 mL each containing 514 μg of bilobalide, 327 μg of ginkgolide A, 327 μg of ginkgolide A, 327 μg of ginkgolide A, and 0.65 mg of ginkgolide C), and then added into 5 portions respectively. Add 2 mL of mixed control solution (each 1 mL containing 514 μg of bilobalide, 327 μg of ginkgolide A, 164 μg of ginkgolide B and 172 μg of ginkgolide C) into a 100 mL volumetric flask, add 0.1 mol/L hydrochloric acid solution to the scale, ultrasonicate the extract for 30 min and inject the sample into 10 μL, and then determine the recoveries by using the chromatography above, and then calculate the recoveries, which were 100.2% for the average recoveries of bilobalide, ginkgolide A, B, C respectively. The average recoveries of bilberry lactone, ginkgolide A, B and C were 100.2%, 98.3%, 99.3% and 98.5%, and the RSDs were 2.0%, 2.1%, 2.6% and 2.4%, respectively.

**1.8 Determination of the content of Xindakang oral preparation (Lin et al., 2007)**

1.8.1 Source of drugs

Sample of Xindakang Tablets (Sichuan Meidakang Pharmaceutical Co., Ltd, batch nos. 060103, 060202, 060203, 060301, 060402, total flavonoids labelled at 5mg/tablet)

1.8.2 Preparation of test solution

Take 20 tablets of this product, remove the coating, precision weighing, research fine, take the appropriate amount (equivalent to 10mg of total flavonoids), precision weighing, placed in a stoppered conical flask, precision add 25mL of methanol, weighing, ultrasonic treatment for 30min, cool, and then weighing. With methanol to make up for the loss of mass, shaking, filtration, discard the initial filtrate, precision measurement of 5mL of renewed filtration, placed in a stoppered conical flask, add methanol 15mL, hydrochloric acid (1 → 2) 5mL, placed in a water bath heated to reflux for 30min, cooled quickly, transferred to a 50mL measuring flask, diluted with methanol to the scale, shaking well, with microporous filtration membrane (0.45μm) filtration, and then the filtrate, that is, the obtained.

1.8.3 Chromatographic conditions and chromatogram

Column: ODS C 18 (250mm×4.6mm, 5μm); Mobile phase: methanol-0.4% phosphoric acid (50:50); Detection wavelength: 370nm; Injection volume: 10μL; Theoretical plate number should be not less than 3000 according to the isorhamnetin peak; Take the sample and treat it according to the method of the preparation of the test solution, and the results were as follows: quercetin, isorhamnetin, kaempferitrin can be compared with the other groups of the sample. reach baseline separation with other metabolites, and the separation with adjacent peaks meets the requirements, and there is no obvious deviation in the content determination, and the chromatogram is shown in Figure 8.

Fig. 8 HPLC chromatograms


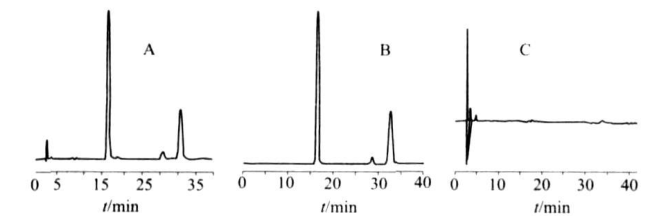


(A)sample. (B)reference substance. (C)negative sample

1.8.4 Sample determination

Samples of cardiac dosage tablets were taken and 6 portions were taken to determine the total amount of quercetin, kaempferin and isorhamnetin, the results were 4.80,4.79,4.82,4.82,4.85,4.83mg, with an RSD of 0.44%. The peak area of the test solution was measured at 0, 3, 6, 12, 24 and 48h after preparation. The results of peak area measurement after 48h are shown in Table 8. RSD were 0.67%, 0% and 0.54% respectively.

Table 8 Results of the stability test

| Time of placement/h | Quercetin/mg | Kaempferol/mg | Isorhamnetin/mg |
| --- | --- | --- | --- |
| 0 | 2.79 | 0.20 | 1.67 |
| 3 | 2.78 | 0.20 | 1.67 |
| 6 | 2.76 | 0.20 | 1.65 |
| 12 | 2.76 | 0.20 | 1.66 |
| 24 | 2.76 | 0.20 | 1.66 |
| 48 | 2.74 | 0.20 | 1.65 |

**1.9 Determination of the content of Xinnaoshutong oral preparation (Zhang et al., 2017)**

1.9.1 Source of drugs

Xinnaoshutong Capsules and Xinnaoshutong Total Saponin (specifically, total saponin of Tribulus terrestris used for the preparation of Xinnaoshutong Capsules without the addition of excipients), supplied by Jilin Aodong Pharmaceutical Co, Ltd, with the batch numbers of 20100903064, 20110107036, 20110201100, 20110701, respectively.

1.9.2 Preparation of test solution

Sample test solution preparation: take about 250 mg of sample, precision weighing, placed in a 5 mL volumetric flask, add 40% of the aqueous acetonitrile 3 mL dissolved, ultrasonic extraction for 30 min, let stand to room temperature, and then add 40% of the aqueous acetonitrile solution to the scale, shaking. 14 000r/min centrifugation for 10 min after the supernatant solution to be measured. Preparation of test solution: take about 75mg of total saponin, weigh precisely, put in 5 mL volumetric flask, add 40% acetonitrile aqueous solution of 3 mL dissolved, ultrasonic extraction for 30 min, let stand to room temperature, add 40% acetonitrile aqueous solution to the scale, shaking. 14 000 r/min centrifugation for 10 min after the supernatant solution to be measured.

1.9.3 Chromatographic conditions and chromatogram

Column: Hedra-C 18 column (4.6 mm×150 mm, 5 μm); mobile phase: acetonitrile (A)-water (B), gradient elution (0~13.5 min, 30% A linearly changed to 39% A, 13.5~15 min, 39% A linearly changed to 95% A, 15~23 min, 95% A linearly unchanged); flow rate: 1.0 mL/min. Column temperature: 40 ℃; drift tube temperature: 60 ℃; gas (N2) pressure: 30 psi; gain value: 100; injection volume: 20 μL; theoretical plate number according to Terrestrinin A is more than 15 000, the separation between Terrestrinin A and the adjacent peaks is not less than 4, and the tailing factor is not more than 1.3, the chromatogram is shown in Figure 9.

Fig. 9 Chromatograms of control (a), sample (b), total saponins (c)


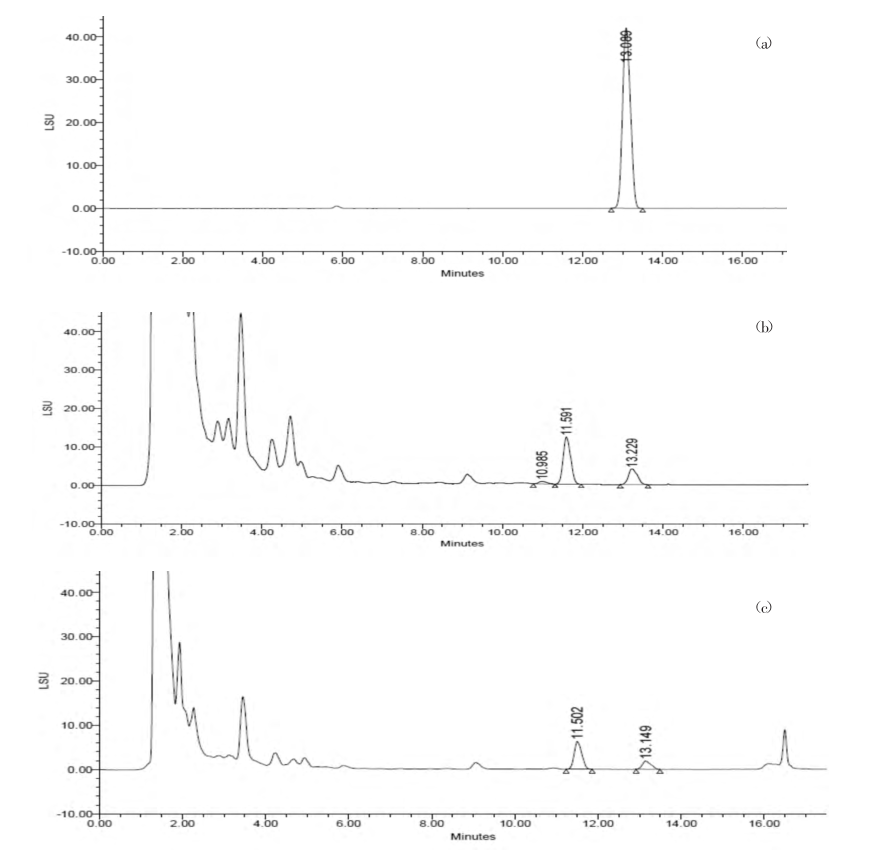


1.9.4 Sample determination

The test solution was prepared by taking Xinnaoshutong capsule and total saponin respectively, and the sample was injected and analyzed according to the above chromatographic conditions, the chromatogram was recorded, and the logarithm of the peak area was used to obtain the logarithm of the concentration of test solution according to the external standard method, and the concentration and the content of the test solution were further calculated. The results of content determination are shown in Table 9.

Table 9 Sample Measurement Results

| Sample | Content (%) |
| --- | --- |
| 20100903064 | 0.39 |
| 20110107036 | 0.45 |
| 20110201100 | 0.45 |
| 20110701 | 2.32 |

**1.10 Determination of the content of Dazhuhongjingtian oral preparation (Huang et al., 2010)**

1.10.1 Source of drugs

Dazhuhongjingtian capsules (batch nos. 090305; 091012) manufactured by Jiangsu Kang Yuan Pharmaceutical Co.10.2 Preparation of test solution.

1.10.2 Preparation of test solution

Precisely weigh the contents of Dazhuhongjingtian capsules (0.1-0.5 g), finely ground, mixed, placed in a 250 ml volumetric flask, add methanol-water (20:80) 15 ml, let stand for 2 h, ultrasonic extraction for 30 min, cooled to room temperature, diluted with methanol-water (20:80) to the scale, in the vortex shaker, mixing for 1 min, and then filtered through a microporous membrane with a diameter of 0.45 μm, that is, it was extracted with a microporous membrane. The test solution was obtained. The negative control solution lacking rhodiola rosea was prepared by the same method.

1.10.3 Chromatographic conditions and chromatogram

Column: SUPELCOSIL (25cm×4.6 mm, 5μm); Mobile phase: methanol-water (20:80); Detection wavelength: 277 nm; Flow rate: 1.0 ml/min; Column temperature: 32℃. Its HPLC graph was recorded and is shown in Figure 10.

Fig. 10 Chromatograms of ten batches of samples


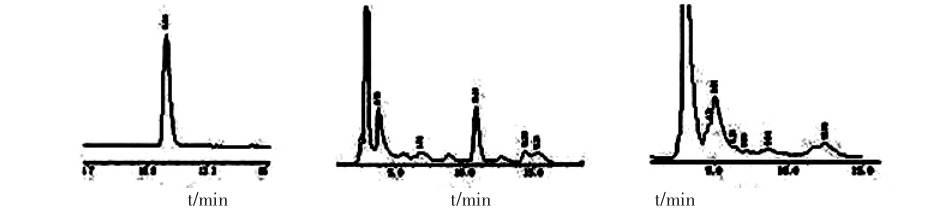


A .Control B. Supplies C. Negative control

10.4 Sample determination

Pipette 10μl of control solution and test solution into liquid chromatograph, determine the peak area according to the above chromatographic conditions, and calculate the content of Rhodiola rosea glycosides in the sample by external standard method. The contents of Rhodiola rosea glycosides in the capsules with batch numbers 090305 and 091012 are 0.92mg and 0.95mg per capsule respectively.

**2. data from the contents of soluble protein and peptide measured by the BCA method and the Bradford method in literature**

**2.1 Determination of the content of Maixuekang oral preparation (Zhong et al., 2024)**

2.1.1 Source of drugs

Maixuekang Capsules (Chongqing Doppler Tai Pharmaceutical Co., Ltd, batch nos. 20200908, 20201005, 20201006 respectively)

2.1.2 Preparation of test solution

Weigh 0.5 g of the content of the capsule, add 2 mL of saline to make the concentration of 250 g-L -1, ultrasonic extraction, centrifuge at 4 000 r-min -1 for 10 min (centrifugation radius 8.7 cm) to obtain the extract. The ultrasonic frequency was fixed at 40 kHz, and the extraction power (80, 120, 200 W) and extraction time (15, 30, 45 min) were examined in terms of the content of total proteins and peptides measured by the BCA method. The BCA kit was used to determine the peptide content of the samples (3 measurements per sample) and to calculate the peptide content of the three preparations. Proteolytic peptide content (%) = (measured proteolytic peptide content × sample dilution) / (weighed sample mass) × 100%.

2.1.3 Chromatographic conditions and chromatogram

Add 5× protein sample buffer or 2× Tris-Tricine-SDS-PAGE sample buffer into 100 g-L -1 sample solution, boil water bath for 5 min to denature the protein, centrifuge at 10 000 r-min -1 for 5 min (centrifugation radius 8.5 cm), and take the supernatant to be measured. Prepare 10% SDS-PAGE gel and 16.5% Tris-Tricine-SDS-PAGE gel. 10 μL of sample was used. Sample 10 μL, set the electrophoresis conditions according to the instructions, and electrophoresis until bromophenol blue reaches the bottom of the gel. Stain, decolorize and expose for imaging. The molecular weight distribution of Cerebral Blood Health Drops was further analyzed by gel chromatography using ÄKTA ™ pure Protein Chromatography Purification System. The chromatographic column was Superdex 30 Increase 10/300GL (10 mm×300 mm, 9 μm), and the eluent was 0.02 mol-L-1PBS+0.25 mol-L-1 NaCl (pH=7.2). The isocratic elution was carried out for 190 min with the detection wavelength of 280 nm at a flow rate of 0.3 mL-min-1, and the injection volume was 500 μL. The flow rate was 0.3 mL-min-1, and the injection volume was 500 μL.

2.1.4 Sample determination

The measured peptide concentration was converted to the peptide content at the lowest single dose (500 mg/dose of pulse hematocrit capsule), which was (48.99±2.52 mg/dose) by the BCA method and (27.38±2.68 mg/dose) by the Bradford method. Combined with the test principle, it is assumed that the capsules mainly contain proteins and peptides above 3 kDa.

Based on the superiority of the separation ranges of the two electrophoreses, the marker of 10% SDS-PAGE was used as the reference for bands above 25 kDa, and the marker of 16.5% Tris-Tricine-SDS-PAGE was used as the reference for bands below 25 kDa. Marker of 10% SDS-PAGE gel was used as a reference for bands above 25 kDa, and Marker of 16.5% Tris-Tricine-SDS-PAGE gel was used as a reference for bands below 25 kDa. The capsule showed 11 bands between 4.0 and 90.0 kDa and 1 distinct band at ~14 kDa. The clearer bands in the >25 kDa range may be due to the degradation of macromolecular proteins during the processing of fresh leeches into leech herbs (scalded and dried). In addition, the capsule had one specific band at ~48.0 kDa, respectively. See Figure 11.

Fig. 11 Electrophoretic bands distribution of protein and peptides of 3 preparations from Hirudo


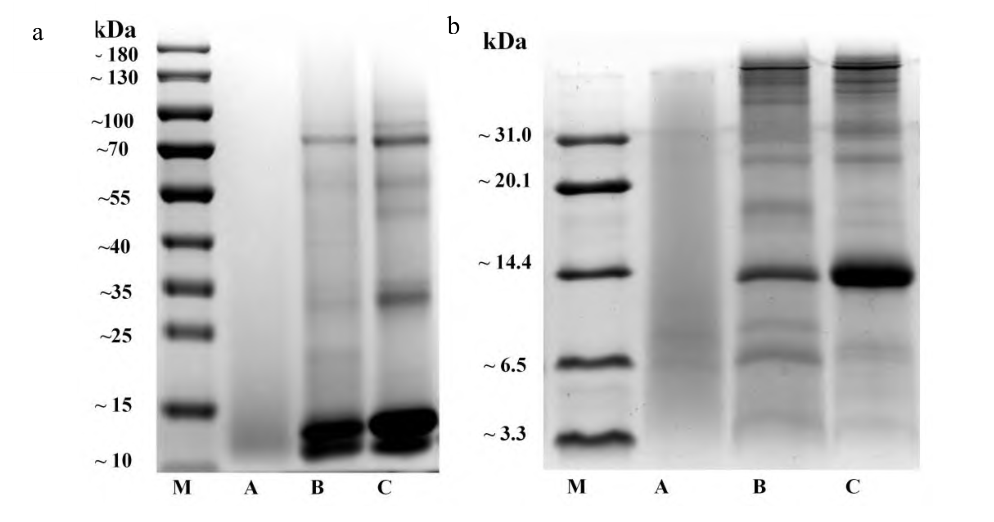


a. 10% SDS-PAGE; b. 16.5% Tris-Tricine-SDS-PAGE; M. Protein polypeptide Maker; A. Naoxuekang drops. B. Contents of Huoxuetongmai capsules C. Contents of Maixuekang capsules.

**References**

Wu, Y., Wei, X., and Ding, Q. (2007). Determination of Ginsenoside Re in Zhenyuan Capsules by HPLC. *Pharm. Clin. Res.* 15, 332-333. doi:10.13664/j.cnki.pcr.2007.04.008

Han, Y., Gu, J., He, X., Zhang, F., and Ji, T. (2015). Content determination of notoginsenoside R1, ginsenoside Rg1, Re, Rb1 in Xuesaitong soft capsules by HPLC. *Chin. J. Drug. Appl. Monit.* 12, 268-271.

Li, H., Du, Q., Wen, R., Xu, P., Du, S., and Lu, Y. (2014). Determination of ginsenosides Rg1 , Re, Rb1 , Rd and notoginsenoside R1 in Xuesaitong capsules by HPLC. *Tianjin. J. Tradit. Chin. Med.* 31, 370-372. doi:10.11656/j.issn.1672-1519.2014.06.15

Zhao, Z., Wang, X., Geng, Y., Zhu, J., Yu, X., and Liu, Y. (2024). Simultaneous Determination of Six Saponins in Xinyue Capsules by UPLC-MS/MS. *Mod. Chin. Med.* 26, 528-534. doi:10.13313/j.issn.1673-4890.20230802002

Zhang, X., Yang, Y., Liu, J., Wang, Y., and Sun, W. (2015). HPLC-ELSD Fingerprint of Steroid Saponins from Di'aoxinxuekang Capsules. *Chin. J. pharm.* 46, 65-67. doi:10.16522/j.cnki.cjph.2015.01.009

Li, Y., and Sun, X. (2019). Simultaneous Content Determination of 8 metabolites in Xuezhikang Capsules by HPLC-DAD. *Chin. Pharm.* 30, 2066-2070. doi:10.6039/j.issn.1001-0408.2019.15.11

Bai, G., Yuan, F., Ye, H., Jiang, W., Qiu, Y., and Chen, X. (2019). Determination of ginkgolides in four kinds of Ginkgo leaf preparations by HPLC-CAD. *Chin. J. Pharm. Anal.* 39, 1102-1107. doi:10.16155/j.0254-1793.2019.06.19

Li, Q., Ma, F., and Cheng, T. (2007). Determination of terpenoid lactones in Ginkgo biloba ester dripping pills by HPLC-ELSD. *Chin. Tradit. Pat. Med.* 29, 836-839. doi:10.3969/j.issn.1001-1528.2007.06.018

Zhou, Z., Su, J., Jin, C., Xu, C., and Qin, Y. (2010). HPLC-ELSD method for determining the dissolution of terpenoids in dispersed tablets of Ginkgo biloba leaf extracts from different manufacturers. *Chin. Tradit. Pat. Med.* 32, 2003-2006. doi:10.3969/j.issn.1001-1528.2010.11.047

Lin, Y., Luo, J., and Wu, L. (2007). Determination of three flavonoids in Xindakang tablets by HPLC. *Chin. J. Hosp. Pharm.* 27, 1320-1322. doi:10.3321/j.issn:1001-5213.2007.09.070

Zhang, N., Luan, X., Xie, Y., Bai, Z., silver, d., Hu, L., et al. (2017). Determination of Terrestrinin A in Xinnaoshutong Capsules by HPLC-ELSD. *J. Tianjin. Univers. Tradit. Chin. Med.* 36, 132-135. doi:10.11656/j.issn.1673-9043.2017.02.14

Huang, M., Jiang, G., and Huang, W. (2010). Determination of Salidroside in Dazhu Hongjingtian Capsules by RP-HPLC. *J. Med. Inf.* 23, 2963. doi:10.3969/j.issn.1006-1959.2010.08.381

Zhong, W., Ma, Y., Ye, J., Fan, X., Shen, H., Yuan, R., et al. (2024). Comparison of Protein and Polypeptide metabolites and Antithrombotic Activity in Vitro of Three Preparations Containing Hirudo. *Chin. J. Exp. Tradit. Med. Formul..* 1-11. doi:10.13422/j.cnki.syfjx.20251067
