# Supplementary material for: Efficacy and safety of 11 oral preparations of single-source traditional Chinese medicines in the treatment of unstable angina pectoris: a systematic review and network meta-analysis
Source: Front Pharmacol. 2025 Jun 24;16:1582661. doi: 10.3389/fphar.2025.1582661 (PMC12235920; doi:10.3389/fphar.2025.1582661)
Supplement: Supplementary file 2 [file Supplementaryfile5.docx]

**Supplement Material 5. Detailed data of Safety evaluation**

Table S22 Occurrence of adverse reactions.

| Interventions | Sample(T/C) | Dizziness | Headache | Flushing | Gastrointestinal distress | Stomach upset | Nausea | Vomiting | Abdominal pain | Abdominal distension |
| --- | --- | --- | --- | --- | --- | --- | --- | --- | --- | --- |
| ZY+CT/CT | 41/41 | 1/1 | N | N | N | N | 1/2 | 1/1 | N | N |
| XST+CT/CT | 294/292 | 2/1 | 2/5 | N | N | 6/1 | N | N | N | N |
| XY+CT/CT | 45/45 | N | N | N | N | N | N | N | N | 3/0 |
| DAXXK+CT/CT | 60/60 | N | N | N | N | N | 1/3 | N | N | N |
| XZK+CT/CT | 351/347 | 0/1 | | N | 1/0 | 1/7 | 9/0 | | 0/1 | 2/1 |
| YXY+CT/CT | 88/88 | 1/2 | N | N | 1/3 | N | N | N | N | N |
| YXTZ+CT/CT | 351/351 | 6/5 | | 4/3 | N | 6/3 | 2/2 | N | N | N |
| XDK+CT/CT | 157/157 | N | 1/1 | N | N | N | N | 3/2 | N | N |
| XNST+CT/CT | 106/108 | N | N | N | N | N | N | N | N | N |
| DZHJT+CT/CT | N | N | N | N | N | N | N | N | N | N |
| MXK+CT/CT | 216/215 | N | 1/0 | N | N | N | N | N | N | N |

Continued Table

| Interventions | Sample(T/C) | Diarrhea | Rash | Subcutaneous hemorrhage | Gingival bleeding | Bleeding | Fatigue | Tachycardia | Hypotension | Abnormalities in liver function |
| --- | --- | --- | --- | --- | --- | --- | --- | --- | --- | --- |
| ZY+CT/CT | 41/41 | N | N | N | N | N | N | N | N | N |
| XST+CT/CT | 294/292 | N | 1/0 | N | 1/0 | N | N | N | N | N |
| XY+CT/CT | 45/45 | N | N | N | N | N | N | N | N | N |
| DAXXK+CT/CT | 60/60 | N | N | N | N | N | N | N | 5/4 | N |
| XZK+CT/CT | 351/347 | N | N | 3/2 | 2/3 | N | N | N | N | 13/8 |
| YXY+CT/CT | 88/88 | N | N | N | N | N | 0/2 | N | N | N |
| YXTZ+CT/CT | 351/351 | N | N | N | N | 3/2 | 3/1 | 6/5 | 0/1 | 1/1 |
| XDK+CT/CT | 157/157 | 1/0 | N | N | N | N | N | N | N | N |
| XNST+CT/CT | 106/108 | N | N | N | N | N | N | N | N | N |
| DZHJT+CT/CT | N | N | N | N | N | N | N | N | N | N |
| MXK+CT/CT | 216/215 | 1/1 | 2/1 | N | N | N | N | N | N | N |

ZY, Zhenyuan oral preparation; XST, Xuesaitong oral preparation; XY, Xinyue oral preparation; DAXXK, Diaoxinxuekang oral preparation; XZK, Xuezhikang oral preparation; YXY, Yinxingye oral preparation; YXTZ, Yinxingtongzhi oral preparation; XDK, Xindakang oral preparation; XNST, Xinnaoshutong oral preparation; DZHJT, Dazhuhongjingtian oral preparation; MXK, Maixuekang oral preparation; CT, conventional treatment; N, not appear.
