# Supplementary material for: Efficacy and safety of 11 oral preparations of single-source traditional Chinese medicines in the treatment of unstable angina pectoris: a systematic review and network meta-analysis
Source: Front Pharmacol. 2025 Jun 24;16:1582661. doi: 10.3389/fphar.2025.1582661 (PMC12235920; doi:10.3389/fphar.2025.1582661)
Supplement: Supplementary file 3 [file Supplementaryfile2.pdf]

Supplement material 2. Search strategy

Table S1. The search strategy for China National Knowledge Infrastructure.

Search strategy	Results
((TKA='振源' or TKA='人参果总皂苷' or TKA='人参果皂苷' or TKA='血塞通' or TKA='三七总皂苷' or TKA='心悦' or TKA='西洋参茎叶总皂苷' or TKA='地奥心血康' or TKA='血脂康' or TKA='红曲' or TKA='红曲米' or TKA='银杏叶' or TKA='银杏叶提取物' or TKA='银杏' or TKA='银杏酮酯' or TKA='金纳多' or TKA='杏灵' or TKA='达纳康' or TKA='心达康' or TKA='醋柳黄酮' or TKA='心脑舒通' or TKA='大株红景天' or TKA='红景天' or TKA='景天' or TKA='脉血康' or TKA='水蛭素') and (TKA='胶囊' or TKA='片' or TKA='散' or TKA='丸' or TKA='膏' or TKA='丹' or TKA='滴丸' or TKA='颗粒' or TKA='口服液')) and (TKA='不稳定型心绞痛' or TKA='不稳定性心绞痛' or TKA='不稳定心绞痛' or TKA='急性冠脉综合征' or TKA='冠状动脉粥样硬化性心脏病' or TKA='冠心病' or TKA='心肌缺血' or TKA='缺血性心脏病' or TKA='心绞痛') and (FT='随机')	1,114

Table S2. The search strategy for China Science and Technology Journal Database.

Search strategy	Results
(题名或关键词=((“振源” or “人参果总皂苷” or “人参果皂苷” or “血塞通” or “三七总皂苷” or “心悦” or “西洋参茎叶总皂苷” or “地奥心血康” or “血脂康” or “红曲” or “红曲米” or “银杏叶” or “银杏叶提取物” or “银杏” or “银杏酮酯” or “金纳多” or “杏灵” or “达纳康” or “心达康” or “醋柳黄酮” or “心脑舒通” or “大株红景天”or “红景天”or “景天” or “脉血康” or “水蛭素”) and (“胶囊” or “片” or “散” or “丸” or “膏” or “丹” or “滴丸” or “颗粒” or “口服液”)) and (“不稳定型心绞痛” or “不稳定性心绞痛” or “不稳定心绞痛” or “急性冠脉综合征” or “冠状动脉粥样硬化性心脏病” or “冠心病”or “心肌缺血” or “缺血性心脏病” or “心绞痛”)) and (全部=(“随机”))	634

Table S3. The search strategy for Wanfang Database.

Search strategy	Results
(M=(振源 OR 人参果总皂苷 OR 人参果皂苷 OR 血塞通 OR 三七总皂苷 OR 心悦 OR 西洋参茎叶总皂苷 OR 地奥心血康 OR 血脂康 OR 红曲 OR 红曲米 OR 银杏叶 OR 银杏叶提取物 OR 银杏 OR 银杏酮酯 OR 金纳多 OR 杏灵 OR 达纳康 OR 心达康 OR 醋柳黄酮 OR 心脑舒通 OR 大株红景天 OR 红景天 OR 景天 OR 脉血康 OR 水蛭素) AND M=(胶囊 OR 片 OR 散 OR 丸 OR 膏 OR 丹 OR 滴丸 OR 颗粒 OR 口服液)) AND (M=(不稳定型心绞痛 OR 不稳定性心绞痛 OR 不稳定心绞痛 OR 急性冠脉综合征 OR 冠状动脉粥样硬化性心脏病 OR 冠心病 OR 心肌缺血 OR 缺血性心脏病 OR 心绞痛)) AND U=随机	386

Table S4. The search strategy for Chinese Biomedical Literature Database.

Number	Search strategy	Results
#5	#1 AND #2 AND #3 AND #4	991
#4	"随机"[全部字段:智能]	2,325,125
#3	"不稳定型心绞痛"[常用字段] OR "不稳定性心绞痛"[常用字段] OR "不稳定心绞痛"[常用字段] OR "急性冠脉综合征"[常用字段] OR "冠状动脉粥样硬化性心脏病"[常用字段] OR "冠心病"[常用字段] OR "心肌缺血"[常用字段] OR "缺血性心脏病"[常用字段] OR "心绞痛"[常用字段]	884,916
#2	"胶囊"[常用字段] OR "片"[常用字段] OR "散"[常用字段] OR "丸"[常用字段] OR "膏"[常用字段] OR "丹"[常用字段] OR "滴丸"[常用字段] OR "颗粒"[常用字段] OR "口服液"[常用字段]	1,972,711
#1	"振源"[常用字段] OR "人参果总皂苷"[常用字段] OR "人参果皂苷"[常用字段] OR "血塞通"[常	46,769

	用字段] OR "三七总皂苷"[常用字段] OR "心悦"[常用字段] OR "西洋参茎叶总皂苷"[常用字段] OR "地奥心血康"[常用字段] OR "血脂康"[常用字段] OR "红曲"[常用字段] OR "银杏叶"[常用字段] OR "银杏叶提取物"[常用字段] OR "银杏"[常用字段] OR "银杏酮酯"[常用字段] OR "金纳多"[常用字段] OR "杏灵"[常用字段] OR "达纳康"[常用字段] OR "心达康"[常用字段] OR "醋柳黄酮"[常用字段] OR "心脑舒通"[常用字段] OR "大株红景天"[常用字段] OR "红景天"[常用字段] OR "景天"[常用字段] OR "脉血康"[常用字段] OR "水蛭素"[常用字段]	

Table S5. The search strategy for Web of Science.

Number	Search strategy	Results
#20	#12 AND #18 AND #19	574
#19	TS=(Randomized controlled trial) OR TS=(Randomized) OR TS=(Random*) OR TS=(Clinical trial) OR TS=(Trial*) OR TS=(Placebo) OR TS=(Placebo*) OR TS=(Groups)	3,597,409
#18	#13 OR #14 OR #15 OR #16 OR #17	512,219
#17	TS=(Angina Pectoris) OR TS=(Angor Pectoris) OR TS=(Stenocardia) OR TS=(Stenocardias)	22,622
#16	TS=(Myocardial Ischemia) OR TS=(Heart Disease, Ischemic) OR TS=(Disease, Ischemic Heart) OR TS=(Diseases, Ischemic Heart) OR TS=(Heart Diseases, Ischemic) OR TS=(Ischemic Heart Diseases) OR TS=(Ischemia, Myocardial) OR TS=(Ischemias, Myocardial) OR TS=(Myocardial Ischemias) OR TS=(Ischemic Heart Disease)	135,606
#15	TS=(Coronary Disease) OR TS=(Coronary Diseases) OR TS=(Disease, Coronary) OR TS=(Diseases, Coronary) OR TS=(Coronary Heart Disease) OR TS=(Coronary Heart Diseases) OR TS=(Disease, Coronary Heart) OR TS=(Diseases, Coronary Heart) OR TS=(Heart Disease, Coronary) OR TS=(Heart Diseases, Coronary)	373,167
#14	TS=(Acute Coronary Syndrome) OR TS=(Acute Coronary Syndromes) OR TS=(Coronary Syndrome, Acute) OR TS=(Coronary Syndromes, Acute) OR TS=(Syndrome, Acute Coronary) OR TS=(Syndromes, Acute Coronary)	65,426
#13	TS=(Angina, Unstable) OR TS=(Anginas, Unstable) OR TS=(Unstable Anginas) OR TS=(Angina at Rest) OR TS=(Angina, Preinfarction) OR TS=(Anginas, Preinfarction) OR TS=(Preinfarction Angina) OR TS=(Preinfarction Anginas) OR TS=(Unstable Angina) OR TS=(Angina Pectoris, Unstable) OR TS=(Angina Pectori, Unstable) OR TS=(Unstable Angina Pectori) OR TS=(Unstable Angina Pectoris) OR TS=(Myocardial Preinfarction Syndrome) OR TS=(Myocardial Preinfarction Syndromes) OR TS=(Preinfarction Syndrome, Myocardial) OR TS=(Preinfarction Syndromes, Myocardial) OR TS=(Syndrome, Myocardial Preinfarction) OR TS=(Syndromes, Myocardial Preinfarction) OR TS=(Syndromes, Myocardial Preinfarction)	22,699
#12	#1 OR #2 OR #3 OR #4 OR #5 OR #6 OR #7 OR #8 OR #9 OR #10	22,017

	OR #11	
#11	TS=(Maixuekang) OR TS=(Mai xue kang)	4,426
#10	TS=(Dazhuhongjingtian) OR TS=(Dazhu hongjingtian) OR TS=(Da zhu hong jing tian) OR TS=(Hongjingtian) OR TS=(Hong jing tian) OR TS=(Jingtian) OR TS=(Jing tian)	67
#9	TS=(Xinnaoshutong) OR TS=(Xin nao shu tong)	3
#8	TS=(Xindakang) OR TS=(Sindacon) OR TS=(Hippophae rhamnoides) OR TS=(Hippophaserhamnoides) OR TS=(Seabuckthorn flavone) OR TS=(Sea buckthorn flavonoid extracted) OR TS=(Total flavonoids of hippophae) OR TS=(Total flavones of hippophae rhamnoides) OR TS=(Total flavonoids of hippophae rhamnoides)	1,989
#7	TS=(Ginkgo ketone ester)	1,235
#6	TS=(Yinxing) OR TS=(Yinxingye) OR TS=(Xingling) OR TS=(Ginaton) OR TS=(Jinnaduo) OR TS=(Danakang) OR TS=(Ginkgo biloba) OR TS=(bilobas, Ginkgo) OR TS=(Ginkgo bilobas) OR TS=(Ginkgo) OR TS=(Ginkgos) OR TS=(Ginko) OR TS=(Ginkos) OR TS=(Maidenhair Tree) OR TS=(Maidenhair Trees) OR TS=(Tree, Maidenhair) OR TS=(Trees, Maidenhair) OR TS=(Gingko biloba) OR TS=(bilobas, Gingko) OR TS=(Gingko bilobas) OR TS=(Gingko) OR TS=(Gingkos) OR TS=(Ginkgophyta) OR TS=(Ginkgophytas)	9,717
#5	TS=(Xuezhikang) OR TS=(Xue zhi kang) OR TS=(Red yeast rice) OR TS=(Monascus) OR TS=(Monascus purpureus) OR TS=(Monascus albidus) OR TS=(Monascus anka) OR TS=(Monascus araneosus) OR TS=(Monascus rubiginosus)	2,988
#4	TS=(Diaoxinxuekang) OR TS=(Di ao xin xue kang) OR TS=(Diao xin xue kang) OR TS=(Di'ao xin xue kang)	7
#3	TS=(Xinyue) OR TS=(Xin yue) OR TS=(Panax quinquefolium saponins)	244
#2	TS=(Xuesaitong) OR TS=(Xue sai tong) OR TS=(Panax notoginseng saponins) OR TS=(Total saponins of panax notoginseng) OR TS=(Panax notoginsenoside) OR TS=(Panax notoginsenosidum) OR TS=(Sanqi total saponins) OR TS=(Total saponins from sanqi)	1,873
#1	TS=(Zhenyuan) OR TS=(Zhen yuan) OR TS=(Total saponins in the fruit of panax ginseng) OR TS=(Ginseng fruit saponins) OR TS=(Panax ginseng berry extract)	290

Table S6. The search strategy for PubMed.

**Number**	**Search strategy**	Results
#34	#16 AND #32 AND #33	146
#33	(Randomized controlled trial[Publication Type] OR Randomized[Title/Abstract] OR Random*[Title/Abstract] OR Clinical trial[Title/Abstract] OR Trial*[Title/Abstract] OR Placebo[Title/Abstract] OR Placebo*[Title/Abstract] OR	4,719,863

	Groups[Title/Abstract])	
#32	#19 OR #22 OR #25 OR #28 OR #31	553,538
#31	#29 OR #30	53,213
#30	(Angina Pectoris[Title/Abstract]) OR (Angor Pectoris[Title/Abstract]) OR (Stenocardia[Title/Abstract]) OR (Stenocardias[Title/Abstract])	22,774
#29	"Angina Pectoris"[Mesh]	45,123
#28	#26 OR #27	507,710
#27	(Myocardial Ischemia[Title/Abstract]) OR (Heart Disease, Ischemic[Title/Abstract]) OR (Disease, Ischemic Heart[Title/Abstract]) OR (Diseases, Ischemic Heart[Title/Abstract]) OR (Heart Diseases, Ischemic[Title/Abstract]) OR (Ischemic Heart Diseases[Title/Abstract]) OR (Ischemia, Myocardial[Title/Abstract]) OR (Ischemias, Myocardial[Title/Abstract]) OR (Myocardial Ischemias[Title/Abstract]) OR (Ischemic Heart Disease[Title/Abstract])	63,410
#26	"Myocardial Ischemia"[Mesh]	485,622
#25	#23 OR #24	277,735
#24	(Coronary Disease[Title/Abstract]) OR (Coronary Diseases[Title/Abstract]) OR (Disease, Coronary[Title/Abstract]) OR (Diseases, Coronary[Title/Abstract]) OR (Coronary Heart Disease[Title/Abstract]) OR (Coronary Heart Diseases[Title/Abstract]) OR (Disease, Coronary Heart[Title/Abstract]) OR (Diseases, Coronary Heart[Title/Abstract]) OR (Heart Disease, Coronary[Title/Abstract]) OR (Heart Diseases, Coronary[Title/Abstract])	80,210
#23	"Coronary Disease"[Mesh]	242,645
#22	#20 OR #21	46,935
#21	(Acute Coronary Syndrome[Title/Abstract]) OR (Acute Coronary Syndromes[Title/Abstract]) OR (Coronary Syndrome, Acute[Title/Abstract]) OR (Coronary Syndromes, Acute[Title/Abstract]) OR (Syndrome, Acute Coronary[Title/Abstract]) OR (Syndromes, Acute Coronary[Title/Abstract])	43,710
#20	"Acute Coronary Syndrome"[Mesh]	21,491
#19	#17 OR #18	21,212
#18	(Angina, Unstable[Title/Abstract]) OR (Anginas, Unstable[Title/Abstract]) OR (Unstable Anginas[Title/Abstract]) OR (Angina at Rest[Title/Abstract]) OR (Angina, Preinfarction[Title/Abstract]) OR (Anginas, Preinfarction[Title/Abstract]) OR (Preinfarction Angina[Title/Abstract]) OR (Preinfarction Anginas[Title/Abstract]) OR (Unstable Angina[Title/Abstract]) OR (Angina Pectoris, Unstable[Title/Abstract]) OR (Angina Pectori, Unstable[Title/Abstract]) OR (Unstable Angina	16,089

	Pectori[Title/Abstract]) OR (Unstable Angina Pectoris[Title/Abstract]) OR (Myocardial Preinfarction Syndrome[Title/Abstract]) OR (Myocardial Preinfarction Syndromes[Title/Abstract]) OR (Preinfarction Syndrome, Myocardial[Title/Abstract]) OR (Preinfarction Syndromes, Myocardial[Title/Abstract]) OR (Syndrome, Myocardial Preinfarction[Title/Abstract]) OR (Syndromes, Myocardial Preinfarction[Title/Abstract])	
#17	"Angina, Unstable"[Mesh]	11,424
#16	#1 OR #2 OR #3 OR #4 OR #7 OR #10 OR #11 OR #12 OR #13 OR #14 OR #15	9,294
#15	(Maixuekang[Title/Abstract]) OR (Mai xue kang[Title/Abstract])Sort by: Most Recent	13
#14	(Dazhuhongjingtian[Title/Abstract]) OR (Dazhu hongjingtian[Title/Abstract]) OR (Da zhu hong jing tian[Title/Abstract]) OR (Hongjingtian[Title/Abstract]) OR (Hong jing tian[Title/Abstract]) OR (Jingtian[Title/Abstract]) OR (Jing tian[Title/Abstract])	42
#13	(Xinnaoshutong[Title/Abstract]) OR (Xin nao shu tong[Title/Abstract])	2
#12	(Xindakang[Title/Abstract]) OR (Sindacon[Title/Abstract]) OR (Hippophae rhamnoides[Title/Abstract]) OR (Hippophaserhamnoides[Title/Abstract]) OR (Seabuckthorn flavone[Title/Abstract]) OR (Sea buckthorn flavonoid extracted[Title/Abstract]) OR (Total flavonoids of hippophae[Title/Abstract]) OR (Total flavones of hippophae rhamnoides[Title/Abstract]) OR (Total flavonoids of hippophae rhamnoides[Title/Abstract])	790
#11	(Ginkgo ketone ester[Title/Abstract])	2
#10	#8 OR #9	6,132
#9	(Yinxing[Title/Abstract]) OR (Yinxingye[Title/Abstract]) OR (Xingling[Title/Abstract]) OR (Ginaton[Title/Abstract]) OR (Jinnaduo[Title/Abstract]) OR (Danakang[Title/Abstract]) OR (Ginkgo biloba[Title/Abstract]) OR (bilobas, Ginkgo[Title/Abstract]) OR (Ginkgo bilobas[Title/Abstract]) OR (Ginkgo[Title/Abstract]) OR (Ginkgos[Title/Abstract]) OR (Ginko[Title/Abstract]) OR (Ginkos[Title/Abstract]) OR (Maidenhair Tree[Title/Abstract]) OR (Maidenhair Trees[Title/Abstract]) OR (Tree, Maidenhair[Title/Abstract]) OR (Trees, Maidenhair[Title/Abstract]) OR (Gingko biloba[Title/Abstract]) OR (bilobas, Gingko[Title/Abstract]) OR (Gingko bilobas[Title/Abstract]) OR (Gingko[Title/Abstract]) OR (Gingkos[Title/Abstract]) OR (Ginkgophyta[Title/Abstract]) OR (Ginkgophytas[Title/Abstract])	5,717
#8	"Ginkgo biloba"[Mesh]	3,669

#7	#5 OR #6	1,650
#6	(Xuezhikang[Title/Abstract]) OR (Xue zhi kang[Title/Abstract]) OR (Red yeast rice[Title/Abstract]) OR (Monascus[Title/Abstract]) OR (Monascus purpureus[Title/Abstract]) OR (Monascus albidus[Title/Abstract]) OR (Monascus anka[Title/Abstract]) OR (Monascus araneosus[Title/Abstract]) OR (Monascus rubiginosus[Title/Abstract])	1,624
#5	"Monascus"[Mesh]	648
#4	Search: (Diaoxinxuekang[Title/Abstract]) OR (Di ao xin xue kang[Title/Abstract]) OR (Diao xin xue kang[Title/Abstract]) OR (Di'ao xin xue kang[Title/Abstract]) Sort by: Most Recent	4
#3	(Xinyue[Title/Abstract]) OR (Xin yue[Title/Abstract]) OR (Panax quinquefolium saponins[Title/Abstract])	28
#2	(Xuesaitong[Title/Abstract]) OR (Xue sai tong[Title/Abstract]) OR (Panax notoginseng saponins[Title/Abstract]) OR (Total saponins of panax notoginseng[Title/Abstract]) OR (Panax notoginsenoside[Title/Abstract]) OR (Panax notoginsenosidum[Title/Abstract]) OR (Sanqi total saponins[Title/Abstract]) OR (Total saponins from sanqi[Title/Abstract])	629
#1	(Zhenyuan[Title/Abstract]) OR (Zhen yuan[Title/Abstract]) OR (Total saponins in the fruit of panax ginseng[Title/Abstract]) OR (Ginseng fruit saponins[Title/Abstract]) OR (Panax ginseng berry extract[Title/Abstract])	35

Table S7. The search strategy for Embase.

**Number**	**Search strategy**	**Results**
#37	#19 AND #35 AND #36	249
#36	'randomized controlled trial':ti,ab,kw OR 'randomized':ti,ab,kw OR 'random*':ti,ab,kw OR 'clinical trial':ti,ab,kw OR 'trial*':ti,ab,kw OR 'placebo':ti,ab,kw OR 'placebo*':ti,ab,kw OR 'groups':ti,ab,kw	6,423,507
#35	#22 OR #25 OR #28 OR #31 OR #34	735,278
#34	#32 OR #33	126,324
#33	'angina pectoris':ti,ab,kw OR 'angor pectoris':ti,ab,kw OR 'stenocardia':ti,ab,kw OR 'stenocardias':ti,ab,kw	29,880
#32	'angina pectoris'/exp OR 'angina pectoris'	126,147
#31	#29 OR #30	166,738
#30	'myocardial ischemia':ti,ab,kw OR 'heart disease, ischemic':ti,ab,kw OR 'disease, ischemic heart':ti,ab,kw OR 'diseases, ischemic heart':ti,ab,kw OR 'heart diseases, ischemic':ti,ab,kw OR 'ischemic heart diseases':ti,ab,kw OR 'ischemia, myocardial':ti,ab,kw OR 'ischemias, myocardial':ti,ab,kw OR 'myocardial ischemias':ti,ab,kw OR 'ischemic heart disease':ti,ab,kw	93,739
#29	'heart muscle ischemia'/exp OR 'heart muscle ischemia'	105,727

#28	#26 OR #27	552,339
#27	'coronary disease':ti,ab,kw OR 'coronary diseases':ti,ab,kw OR 'disease, coronary':ti,ab,kw OR 'diseases, coronary':ti,ab,kw OR 'coronary heart disease':ti,ab,kw OR 'coronary heart diseases':ti,ab,kw OR 'disease, coronary heart':ti,ab,kw OR 'diseases, coronary heart':ti,ab,kw OR 'heart disease, coronary':ti,ab,kw OR 'heart diseases, coronary':ti,ab,kw	113,390
#26	'coronary artery disease'/exp OR 'coronary artery disease'	482,750
#25	#23 OR #24	99,390
#24	'acute coronary syndrome':ti,ab,kw OR 'acute coronary syndromes':ti,ab,kw OR 'coronary syndrome, acute':ti,ab,kw OR 'coronary syndromes, acute':ti,ab,kw OR 'syndrome, acute coronary':ti,ab,kw OR 'syndromes, acute coronary':ti,ab,kw	76,984
#23	'acute coronary syndrome'/exp OR 'acute coronary syndrome'	93,033
#22	#20 OR #21	35,416
#21	'angina, unstable':ti,ab,kw OR 'anginas, unstable':ti,ab,kw OR 'unstable anginas':ti,ab,kw OR 'angina at rest':ti,ab,kw OR 'angina, preinfarction':ti,ab,kw OR 'anginas, preinfarction':ti,ab,kw OR 'preinfarction angina':ti,ab,kw OR 'preinfarction anginas':ti,ab,kw OR 'unstable angina':ti,ab,kw OR 'angina pectoris, unstable':ti,ab,kw OR 'angina pectori, unstable':ti,ab,kw OR 'unstable angina pectori':ti,ab,kw OR 'unstable angina pectoris':ti,ab,kw OR 'myocardial preinfarction syndrome':ti,ab,kw OR 'myocardial preinfarction syndromes':ti,ab,kw OR 'preinfarction syndrome, myocardial':ti,ab,kw OR 'preinfarction syndromes, myocardial':ti,ab,kw OR 'syndrome, myocardial preinfarction':ti,ab,kw OR 'syndromes, myocardial preinfarction':ti,ab,kw	23,613
#20	'unstable angina pectoris'/exp OR 'unstable angina pectoris'	30,077
#19	#1 OR #2 OR #3 OR #4 OR #8 OR #11 OR #12 OR #15 OR #16 OR #17 OR #18	17,930
#18	'maixuekang':ti,ab,kw OR 'mai xue kang':ti,ab,kw	17
#17	'dazhuhongjingtian':ti,ab,kw OR 'dazhu hongjingtian':ti,ab,kw OR 'da zhu hong jing tian':ti,ab,kw OR 'hongjingtian':ti,ab,kw OR 'hong jing tian':ti,ab,kw OR 'jingtian':ti,ab,kw OR 'jing tian':ti,ab,kw	61
#16	'xinnaoshutong':ti,ab,kw OR 'xin nao shu tong':ti,ab,kw	2
#15	#13 OR #14	1,446
#14	('xindakang':ti,ab,kw OR 'sindacon':ti,ab,kw OR 'hippophae rhamnoides':ti,ab,kw OR 'hippophaserhamnoides':ti,ab,kw OR 'seabuckthorn flavone':ti,ab,kw OR 'sea buckthorn flavonoid extracted':ti,ab,kw OR 'total flavonoids of hippophae':ti,ab,kw OR 'total flavones of hippophae rhamnoides':ti,ab,kw OR 'total flavonoids of hippophae rhamnoides':ti,ab,kw)	911
#13	'hippophae rhamnoides'/exp OR 'hippophae rhamnoides'	1,442

#12	'Ginkgo ketone ester':ti,ab,kw	3
#11	#9 OR #10	13,313
#10	'yinxing':ti,ab,kw OR 'yinxingye':ti,ab,kw OR 'xingling':ti,ab,kw OR 'ginaton':ti,ab,kw OR 'jinnaduo':ti,ab,kw OR 'danakang':ti,ab,kw OR 'ginkgo biloba':ti,ab,kw OR 'bilobas, ginkgo':ti,ab,kw OR 'ginkgo bilobas':ti,ab,kw OR 'ginkgo':ti,ab,kw OR 'ginkgos':ti,ab,kw OR 'ginko':ti,ab,kw OR 'ginkos':ti,ab,kw OR 'maidenhair tree':ti,ab,kw OR 'maidenhair trees':ti,ab,kw OR 'tree, maidenhair':ti,ab,kw OR 'trees, maidenhair':ti,ab,kw OR 'gingko biloba':ti,ab,kw OR 'bilobas, gingko':ti,ab,kw OR 'gingko bilobas':ti,ab,kw OR 'gingko':ti,ab,kw OR 'gingkos':ti,ab,kw OR 'ginkgophyta':ti,ab,kw OR 'ginkgophytas':ti,ab,kw	7,724
#9	'ginkgo biloba'/exp OR 'ginkgo biloba'	12,636
#8	#5 OR #6 OR #7	2,196
#7	'xuezhikang':ti,ab,kw OR 'xue zhi kang':ti,ab,kw OR 'red yeast rice':ti,ab,kw OR 'monascus':ti,ab,kw OR 'monascus purpureus':ti,ab,kw OR 'monascus albidus':ti,ab,kw OR 'monascus anka':ti,ab,kw OR 'monascus araneosus':ti,ab,kw OR 'monascus rubiginosus':ti,ab,kw	1,942
#6	'monascus'/exp OR 'monascus'	1,437
#5	'xuezhikang'/exp OR 'xuezhikang'	371
#4	'diaoxinxuekang':ti,ab,kw OR 'di ao xin xue kang':ti,ab,kw OR 'di*ao xin xue kang':ti,ab,kw	8
#3	'xinyue':ti,ab,kw OR 'xin yue':ti,ab,kw OR 'panax quinquefolium saponins':ti,ab,kw	44
#2	'xuesaitong':ti,ab,kw OR 'xue sai tong':ti,ab,kw OR 'panax notoginseng saponins':ti,ab,kw OR 'total saponins of panax notoginseng':ti,ab,kw OR 'panax notoginsenoside':ti,ab,kw OR 'panax notoginsenosidum':ti,ab,kw OR 'sanqi total saponins':ti,ab,kw OR 'total saponins from sanqi':ti,ab,kw	883
#1	'zhenyuan':ti,ab,kw OR 'zhen yuan':ti,ab,kw OR 'total saponins in the fruit of panax ginseng':ti,ab,kw OR 'ginseng fruit saponins':ti,ab,kw OR 'panax ginseng berry extract':ti,ab,kw	55

Table S8. The search strategy for Cochrane.

**Search**	**Query**	**Results**
#34	#16 AND #32 AND #33	131
#33	(Randomized controlled trial):ti,ab,kw OR (Randomized):ti,ab,kw OR (Random*):ti,ab,kw OR (Clinical trial):ti,ab,kw OR (Trial*):ti,ab,kw OR (Placebo):ti,ab,kw OR (Placebo*):ti,ab,kw OR (Groups):ti,ab,kw	1,701,055
#32	#19 or #22 or #25 or #28 or #31	73,940
#31	#29 OR #30	11,917
#30	(Angina Pectoris):ti,ab,kw OR (Angor Pectoris):ti,ab,kw OR (Stenocardia):ti,ab,kw OR (Stenocardias):ti,ab,kw	10,900

#29	MeSH descriptor: [Angina Pectoris] explode all trees	5,691
#28	#26 OR #27	51,110
#27	(Myocardial Ischemia):ti,ab,kw OR (Heart Disease, Ischemic):ti,ab,kw OR (Disease, Ischemic Heart):ti,ab,kw OR (Diseases, Ischemic Heart):ti,ab,kw OR (Heart Diseases, Ischemic):ti,ab,kw OR (Ischemic Heart Diseases):ti,ab,kw OR (Ischemia, Myocardial):ti,ab,kw OR (Ischemias, Myocardial):ti,ab,kw OR (Myocardial Ischemias):ti,ab,kw OR (Ischemic Heart Disease):ti,ab,kw	22,113
#26	MeSH descriptor: [Myocardial Ischemia] explode all trees	39,287
#25	#23 OR #24	45,994
#24	(Coronary Disease):ti,ab,kw OR (Coronary Diseases):ti,ab,kw OR (Disease, Coronary):ti,ab,kw OR (Diseases, Coronary):ti,ab,kw OR (Coronary Heart Disease):ti,ab,kw OR (Coronary Heart Diseases):ti,ab,kw OR (Disease, Coronary Heart):ti,ab,kw OR (Diseases, Coronary Heart):ti,ab,kw OR (Heart Disease, Coronary):ti,ab,kw OR (Heart Diseases, Coronary):ti,ab,kw	19,248
#23	MeSH descriptor: [Coronary Disease] explode all trees	19,248
#22	#20 OR #21	9,654
#21	(Acute Coronary Syndrome):ti,ab,kw OR (Acute Coronary Syndromes):ti,ab,kw OR (Coronary Syndrome, Acute):ti,ab,kw OR (Coronary Syndromes, Acute):ti,ab,kw OR (Syndrome, Acute Coronary):ti,ab,kw OR (Syndromes, Acute Coronary):ti,ab,kw	9,654
#20	MeSH descriptor: [Acute Coronary Syndrome] explode all trees	3,128
#19	#17 OR #18	5924
#18	(Angina, Unstable):ti,ab,kw OR (Anginas, Unstable):ti,ab,kw OR (Unstable Anginas):ti,ab,kw OR (Angina at Rest):ti,ab,kw OR (Angina, Preinfarction):ti,ab,kw OR (Anginas, Preinfarction):ti,ab,kw OR (Preinfarction Angina):ti,ab,kw OR (Preinfarction Anginas):ti,ab,kw OR (Unstable Angina):ti,ab,kw OR (Angina Pectoris, Unstable):ti,ab,kw OR (Angina Pectori, Unstable):ti,ab,kw OR (Unstable Angina Pectori):ti,ab,kw OR (Unstable Angina Pectoris):ti,ab,kw OR (Myocardial Preinfarction Syndrome):ti,ab,kw OR (Myocardial Preinfarction Syndromes):ti,ab,kw OR (Preinfarction Syndrome, Myocardial):ti,ab,kw OR (Preinfarction Syndromes, Myocardial):ti,ab,kw OR (Syndrome, Myocardial Preinfarction):ti,ab,kw OR (Syndromes, Myocardial Preinfarction):ti,ab,kw OR (Syndromes, Myocardial Preinfarction):ti,ab,kw	5,850
#17	MeSH descriptor: [Angina, Unstable] explode all trees	1,462
#16	#1 OR #2 OR #3 OR #4 OR #7 OR #10 OR #11 OR #12 OR #13 OR #14 OR #15	2,132
#15	(Maixuekang):ti,ab,kw OR (Mai xue kang):ti,ab,kw	12
#14	(Dazhuhongjingtian):ti,ab,kw OR (Dazhu hongjingtian):ti,ab,kw OR (Da zhu hong jing tian):ti,ab,kw OR (Hongjingtian):ti,ab,kw OR (Hong	14

	jing tian):ti,ab,kw OR (Jingtian):ti,ab,kw OR (Jing tian):ti,ab,kw	
#13	(Xinnaoshutong):ti,ab,kw OR (Xin nao shu tong):ti,ab,kw	3
#12	(Xindakang):ti,ab,kw OR (Sindacon):ti,ab,kw OR (Hippophae rhamnoides):ti,ab,kw OR (Hippophaserhamnoides):ti,ab,kw OR (Seabuckthorn flavone):ti,ab,kw OR (Sea buckthorn flavonoid extracted):ti,ab,kw OR (Total flavonoids of hippophae):ti,ab,kw OR (Total flavones of hippophae rhamnoides):ti,ab,kw OR (Total flavonoids of hippophae rhamnoides):ti,ab,kw	48
#11	(Ginkgo ketone ester):ti,ab,kw	4
#10	#8 OR #9	1,365
#9	(Yinxing):ti,ab,kw OR (Yinxingye):ti,ab,kw OR (Xingling):ti,ab,kw OR (Ginaton):ti,ab,kw OR (Jinnaduo):ti,ab,kw OR (Danakang):ti,ab,kw OR (Ginkgo biloba):ti,ab,kw OR (bilobas, Ginkgo):ti,ab,kw OR (Ginkgo bilobas):ti,ab,kw OR (Ginkgo):ti,ab,kw OR (Ginkgos):ti,ab,kw OR (Ginko):ti,ab,kw OR (Ginkos):ti,ab,kw OR (Maidenhair Tree):ti,ab,kw OR (Maidenhair Trees):ti,ab,kw OR (Tree, Maidenhair):ti,ab,kw OR (Trees, Maidenhair):ti,ab,kw OR (Gingko biloba):ti,ab,kw OR (bilobas, Gingko):ti,ab,kw OR (Gingko bilobas):ti,ab,kw OR (Gingko):ti,ab,kw OR (Gingkos):ti,ab,kw OR (Ginkgophyta):ti,ab,kw OR (Ginkgophytas):ti,ab,kw	1,365
#8	MeSH descriptor: [Ginkgo biloba] explode all trees	398
#7	#5 OR #6	493
#6	(Xuezhikang):ti,ab,kw OR (Xue zhi kang):ti,ab,kw OR (Red yeast rice):ti,ab,kw OR (Monascus):ti,ab,kw OR (Monascus purpureus):ti,ab,kw OR (Monascus albidus):ti,ab,kw OR (Monascus anka):ti,ab,kw OR (Monascus araneosus):ti,ab,kw OR (Monascus rubiginosus):ti,ab,kw	493
#5	MeSH descriptor: [Monascus] explode all trees	10
#4	(Diaoxinxuekang):ti,ab,kw OR (Di ao xin xue kang):ti,ab,kw OR (Diao xin xue kang):ti,ab,kw OR (Di'ao xin xue kang):ti,ab,kw	9
#3	(Xinyue):ti,ab,kw OR (Xin yue):ti,ab,kw OR (Panax quinquefolium saponins):ti,ab,kw	12
#2	(Xuesaitong):ti,ab,kw OR (Xue sai tong):ti,ab,kw OR (Panax notoginseng saponins):ti,ab,kw OR (Total saponins of panax notoginseng):ti,ab,kw OR (Panax notoginsenoside):ti,ab,kw OR (Panax notoginsenosidum):ti,ab,kw OR (Sanqi total saponins):ti,ab,kw OR (Total saponins from sanqi):ti,ab,kw	158
#1	(Zhenyuan):ti,ab,kw OR (Zhen yuan):ti,ab,kw OR (Total saponins in the fruit of panax ginseng):ti,ab,kw OR (Ginseng fruit saponins):ti,ab,kw OR (Panax ginseng berry extract):ti,ab,kw	21
