# Supplementary material for: Efficacy and safety of 11 oral preparations of single-source traditional Chinese medicines in the treatment of unstable angina pectoris: a systematic review and network meta-analysis
Source: Front Pharmacol. 2025 Jun 24;16:1582661. doi: 10.3389/fphar.2025.1582661 (PMC12235920; doi:10.3389/fphar.2025.1582661)
Supplement: Supplementary file 4 [file Supplementaryfile4.docx]

**Supplement Material 4. Surface under the cumulative ranking curve (SUCRA) values of different outcomes**

Table S21. Surface under the cumulative ranking curve (SUCRA) values of different outcomes.

| Interventions | Primary outcome measures | | Secondary outcome measures | | | | | | | | | |
| --- | --- | --- | --- | --- | --- | --- | --- | --- | --- | --- | --- | --- |
|  | Angina efficacy | ECG efficacy | Nitroglycerin dosages | Frequency of angina | Duration of angina | TC | TG | LDL-C | HDL-C | hs-CRP | PV | MACEs |
| ZY+CT | 47.9% | 52.6% | - | 96.9% | 48.5% | - | - | - | - | 35.9% | - | - |
| XST+CT | 46% | 52.6% | 42.2% | 47.5% | 38% | 52.1% | 34% | 47.3% | 47.8% | 91.6% | 16.2% | 42.4% |
| XY+CT | 82.1% | - | 47.9% | 39.1% | 56.3% | - | - | - | - | - | - | - |
| DAXXK+CT | 59.1% | 33% | 54.5% | 79.7% | 56.4% | - | - | - | - | 41.5% | 82.6% | - |
| XZK+CT | 61.3% | 60.7% | - | 36.5% | 35% | 91.6% | 72.5% | 76.8% | 92.5% | 69.8% | - | 97.6% |
| YXY+CT | 30.2% | 41.3% | 37.6% | 38.6% | 34.4% | - | - | - | - | 84.7% | 29.6% | - |
| YXTZ+CT | 55.1% | 81.3% | 66.6% | 64.4% | 79.2% | 71.8% | 82.2% | 55.8% | 40.2% | 47% | 99.9% | - |
| XDK+CT | 72.5% | 99.3% | 92.3% | 62.7% | - | 45.1% | 27.3% | - | 59.1% | - | - | - |
| XNST+CT | 8.2% | - | 28.6% | 17.5% | 86.2% | - | - | - | - | 38.9% | - | - |
| DZHJT+CT | 47.7% | 40.7% | 61% | 38.1% | - | - | - | - | - | 38.3% | 64.4% | - |
| MXK+CT | 86.6% | 38% | - | 67.9% | 60.1% | 49.8% | 75.1% | 67.9% | - | 43.7% | 46.8% | - |

ECG efficacy, Electrocardiogram efficacy; TC, Total cholesterol; TG, Triglyceride; LDL-C, Low-density Lipoprotein Cholesterol; HDL-C, High-density Lipoprotein Cholesterol; hs-CRP, high-sensitivity C-reactive Protein; PV, Plasma viscosity; MACE, Major adverse cardiovascular events; ZY, Zhenyuan oral preparation; XST, Xuesaitong oral preparation; XY, Xinyue oral preparation; DAXXK, Diaoxinxuekang oral preparation; XZK, Xuezhikang oral preparation; YXY, Yinxingye oral preparation; YXTZ, Yinxingtongzhi oral preparation; XDK, Xindakang oral preparation; XNST, Xinnaoshutong oral preparation; DZHJT, Dazhuhongjingtian oral preparation; MXK:Maixuekang oral preparation.
