# Supplementary material for: Efficacy and safety of 11 oral preparations of single-source traditional Chinese medicines in the treatment of unstable angina pectoris: a systematic review and network meta-analysis
Source: Front Pharmacol. 2025 Jun 24;16:1582661. doi: 10.3389/fphar.2025.1582661 (PMC12235920; doi:10.3389/fphar.2025.1582661)
Supplement: Supplementary file 5 [file Supplementaryfile3.pdf]

Supplement material 3

Risk of bias assessment

Figure S1. Risk of bias assessment of included studies

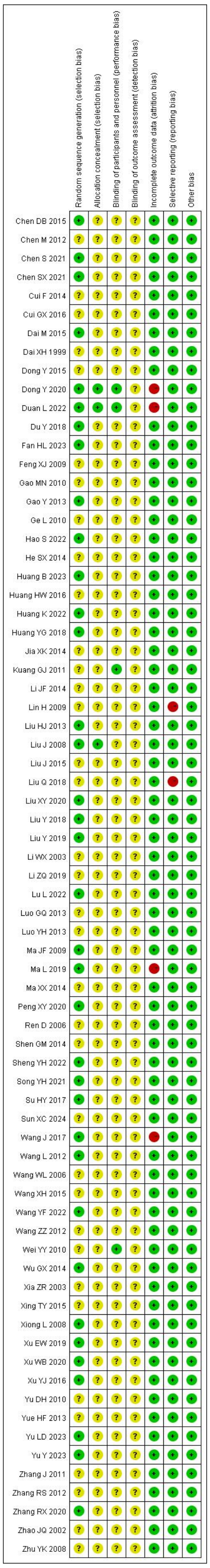

Results of a two-by-two comparison

Figure S2 Angina Efficacy in a two-by-two Comparison

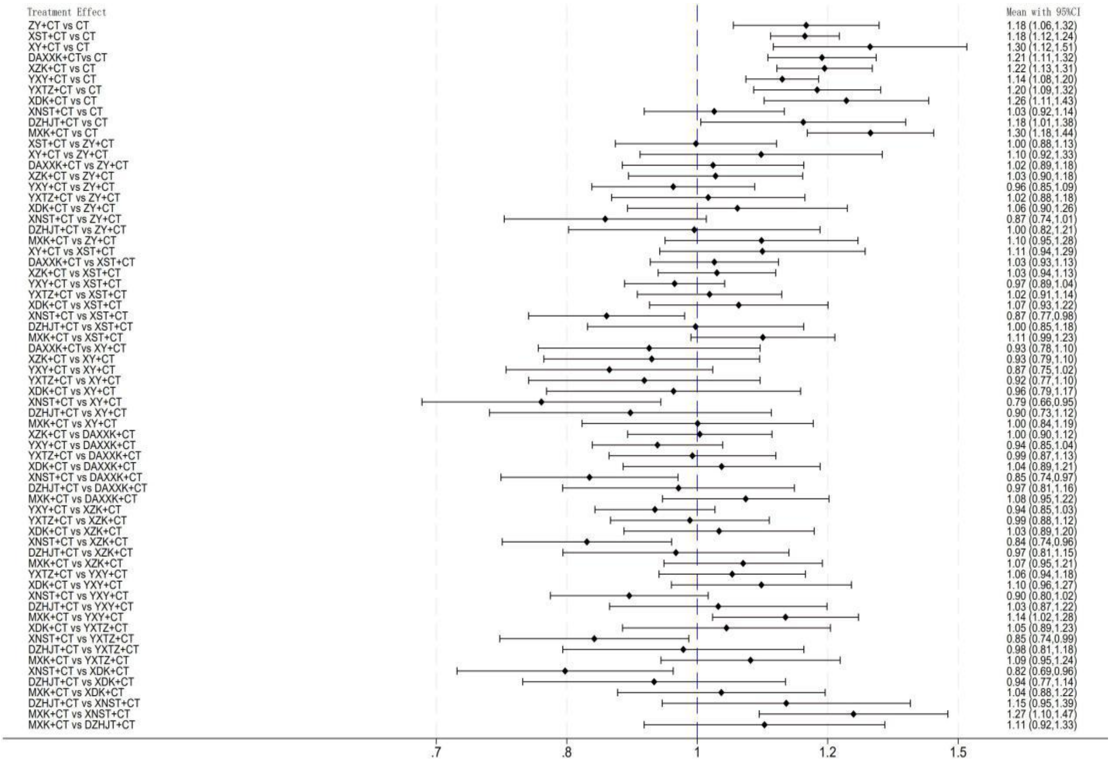

Figure S3 ECG efficacy in a two-by-two Comparison

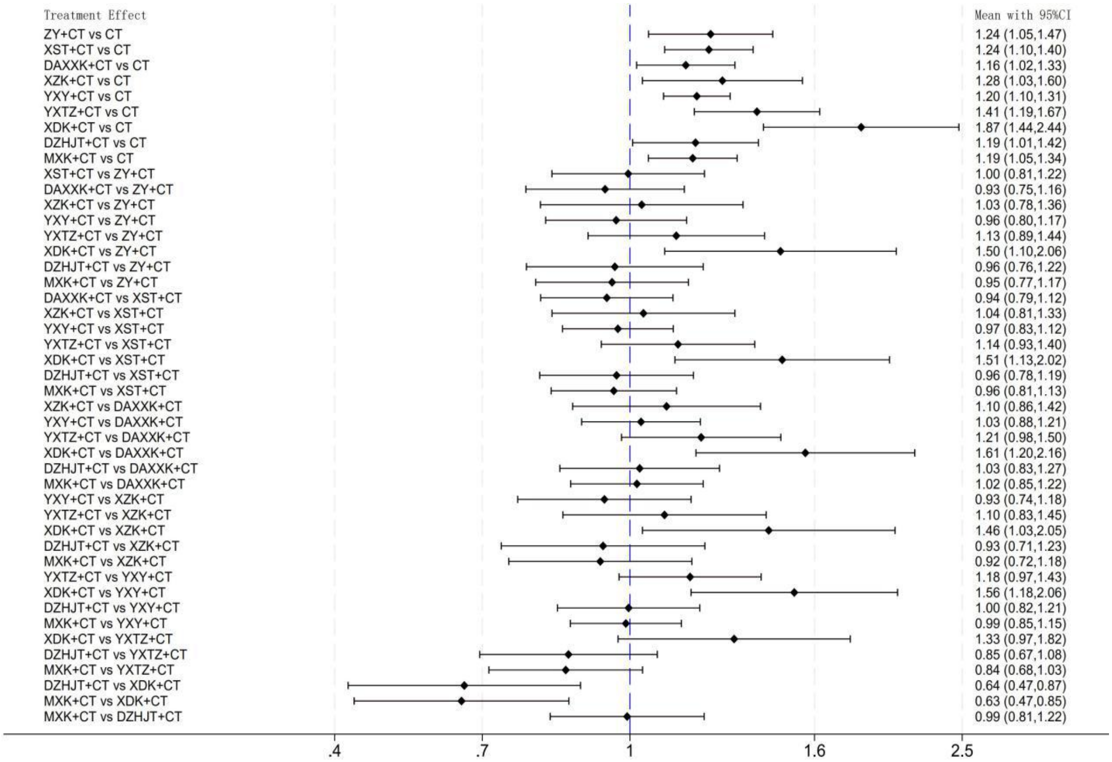

Figure S4 Nitroglycerin dosages in a two-by-two Comparison

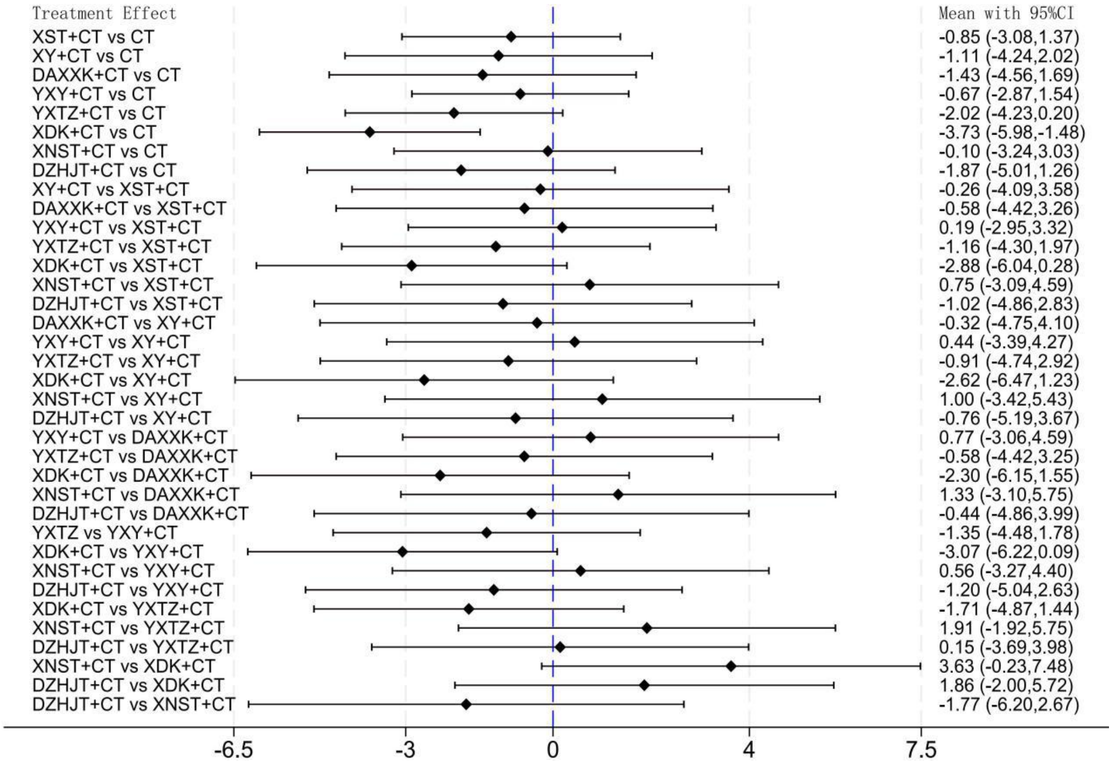

Figure S5 Frequency of angina in a two-by-two Comparison

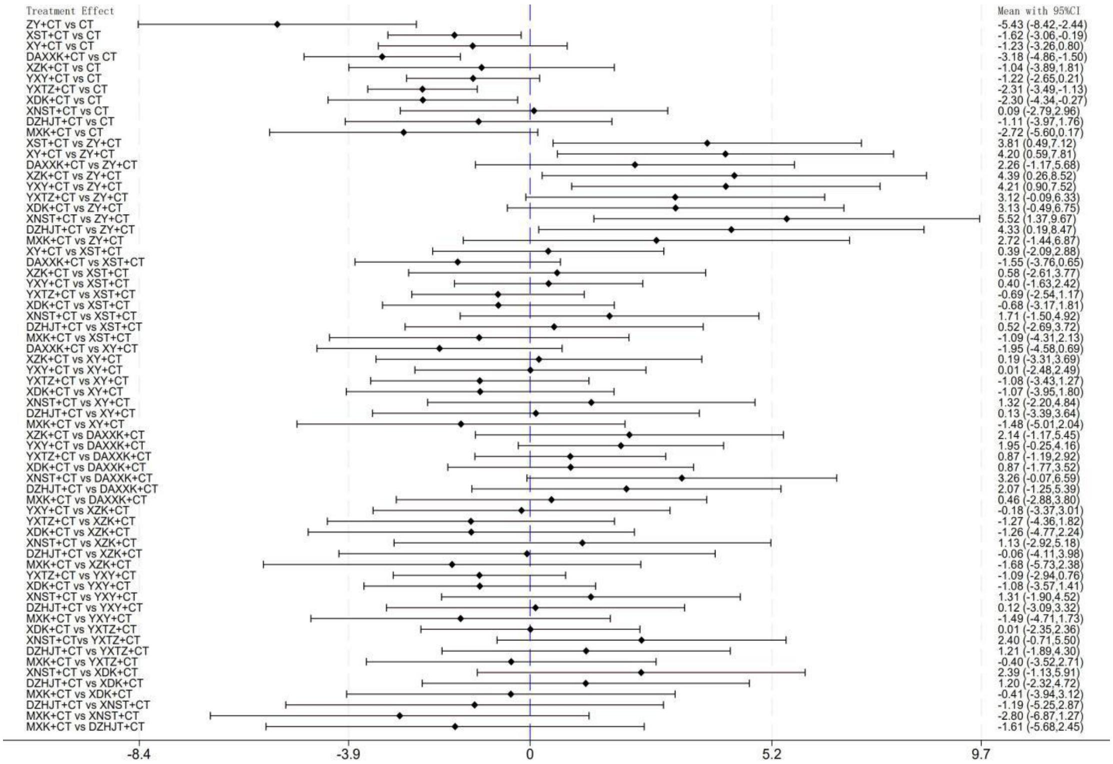

Figure S6 Duration of angina in a two-by-two Comparison

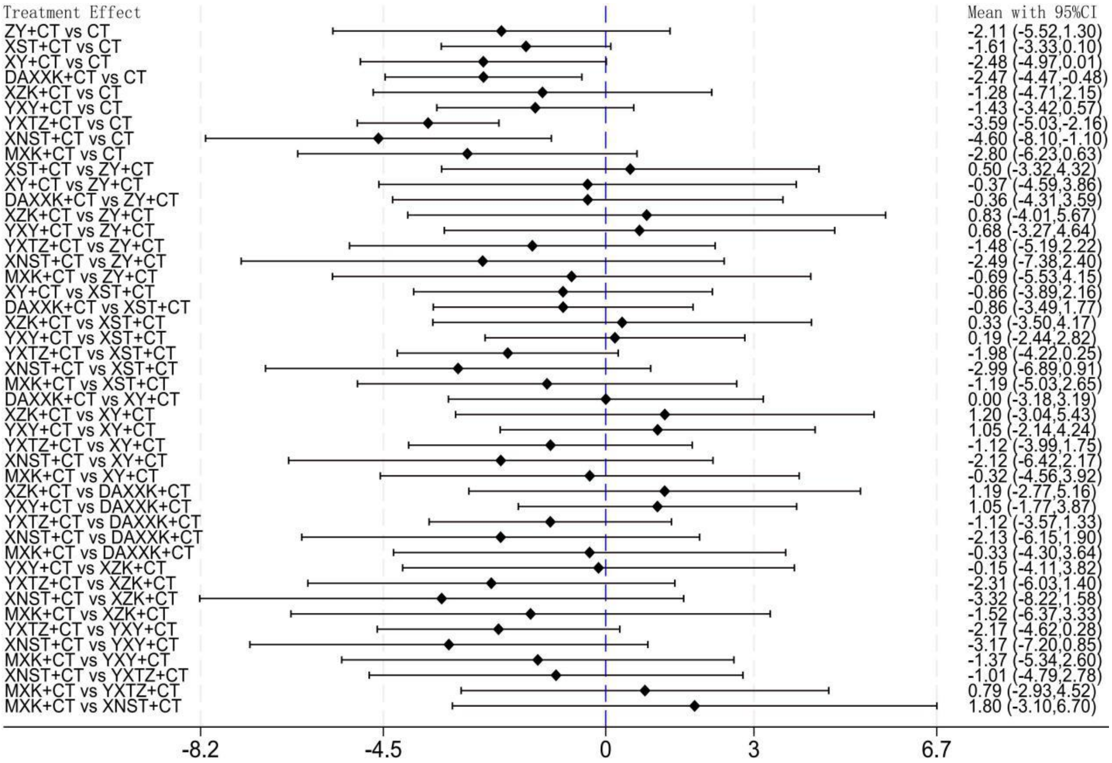

Figure S7 Total cholesterol (TC) in a two-by-two Comparison

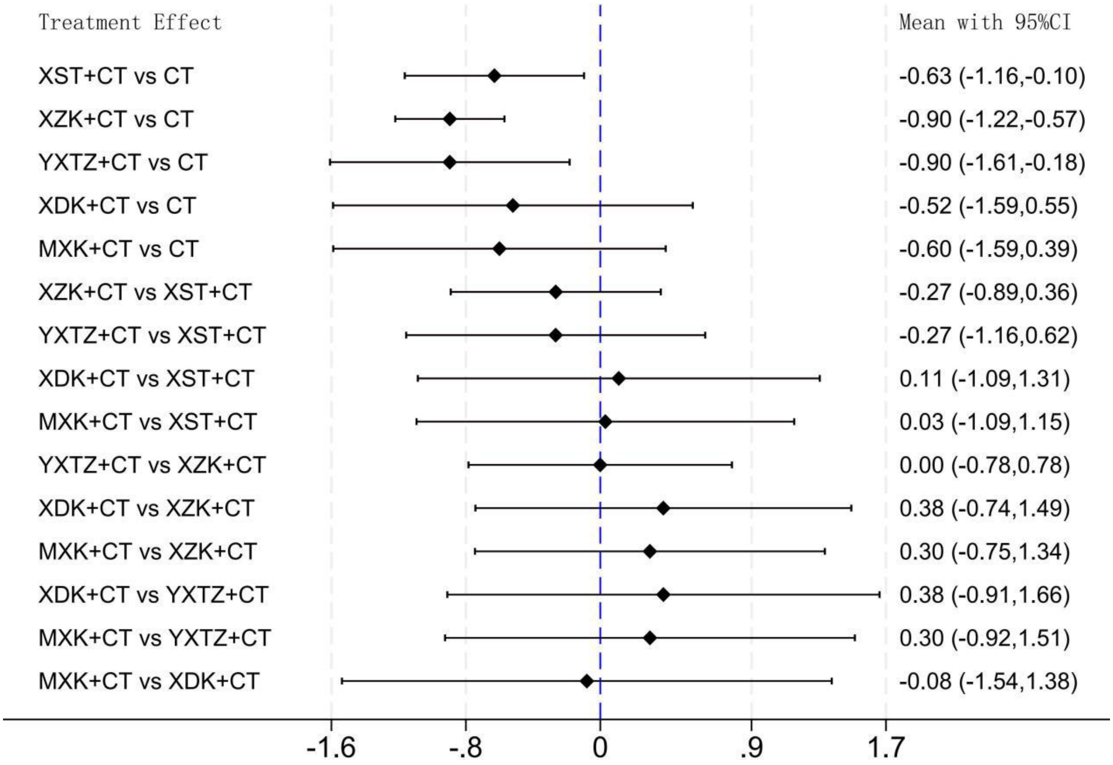

Figure S8 Triglyceride (TG) in a two-by-two Comparison

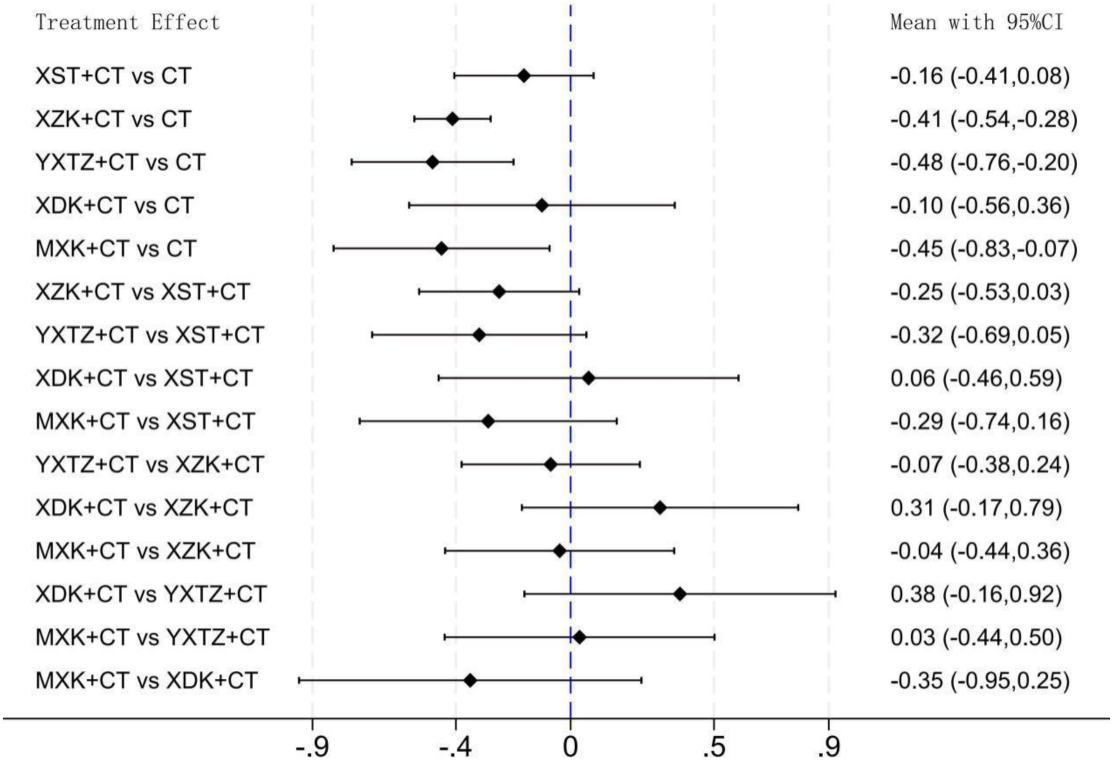

Figure S9 Low-density lipoprotein cholesterol (LDL-C) in a two-by-two Comparison

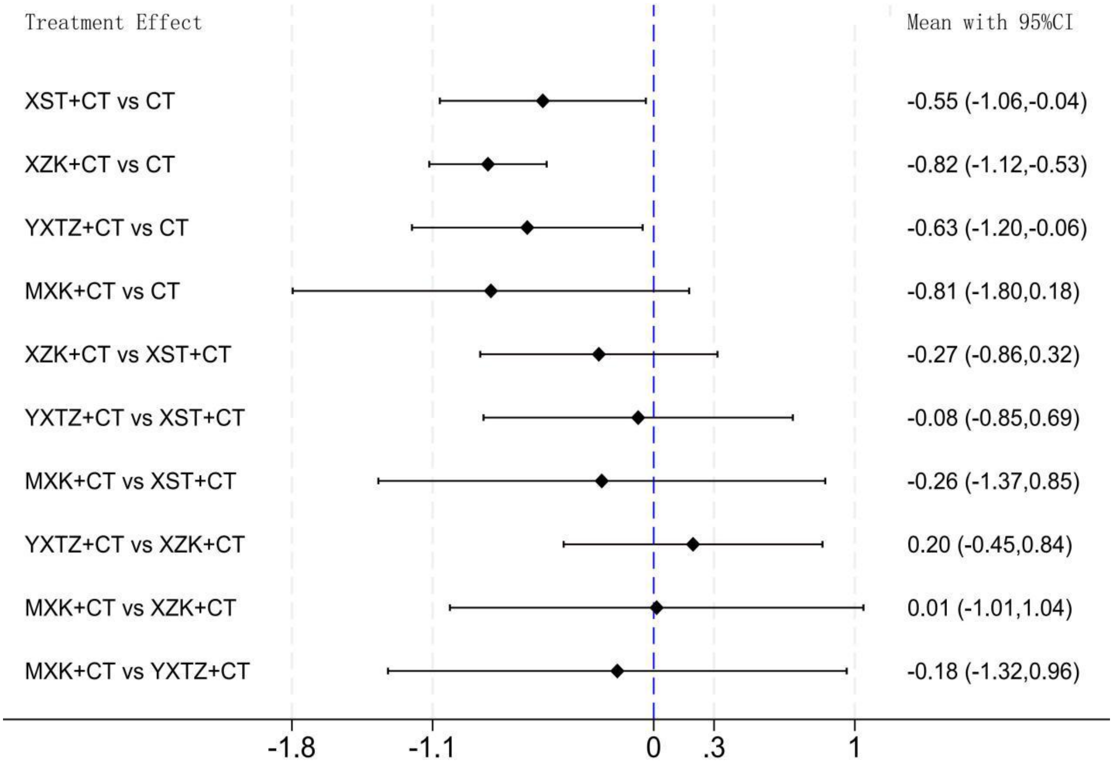

Figure S10 High-density Lipoprotein Cholesterol (HDL-C) in a two-by-two Comparison

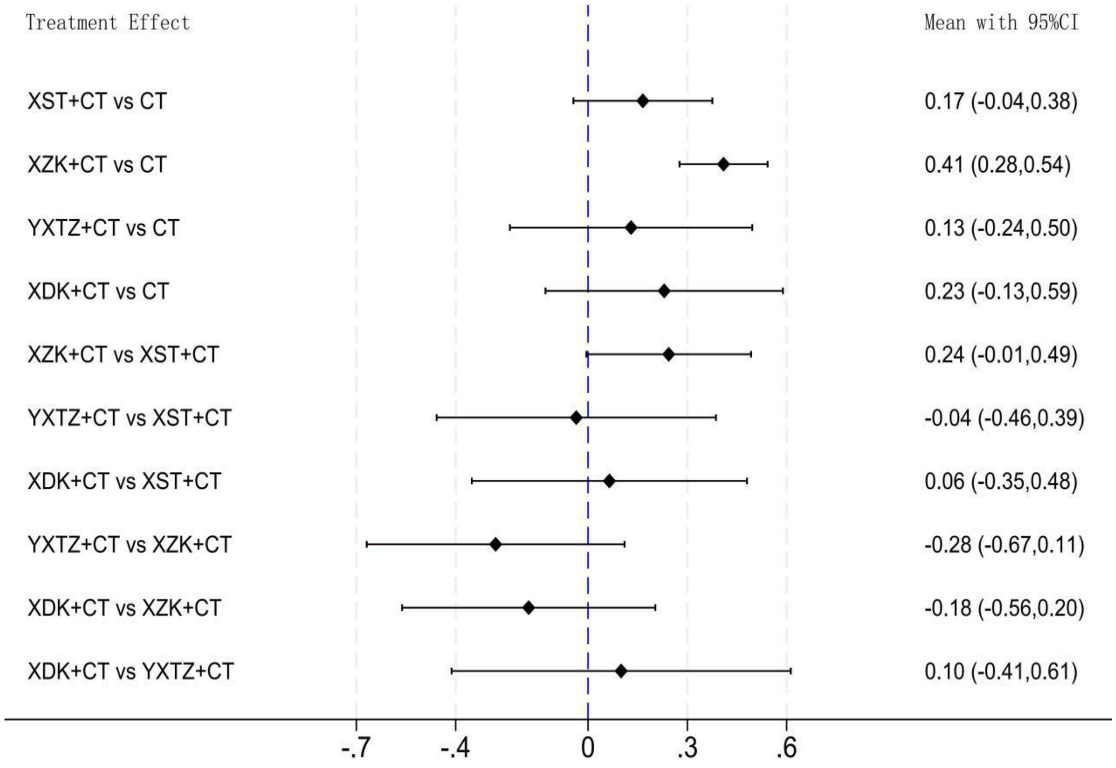

Figure S11 High-sensitivity C-reactive Protein (hs-CRP) in a two-by-two Comparison

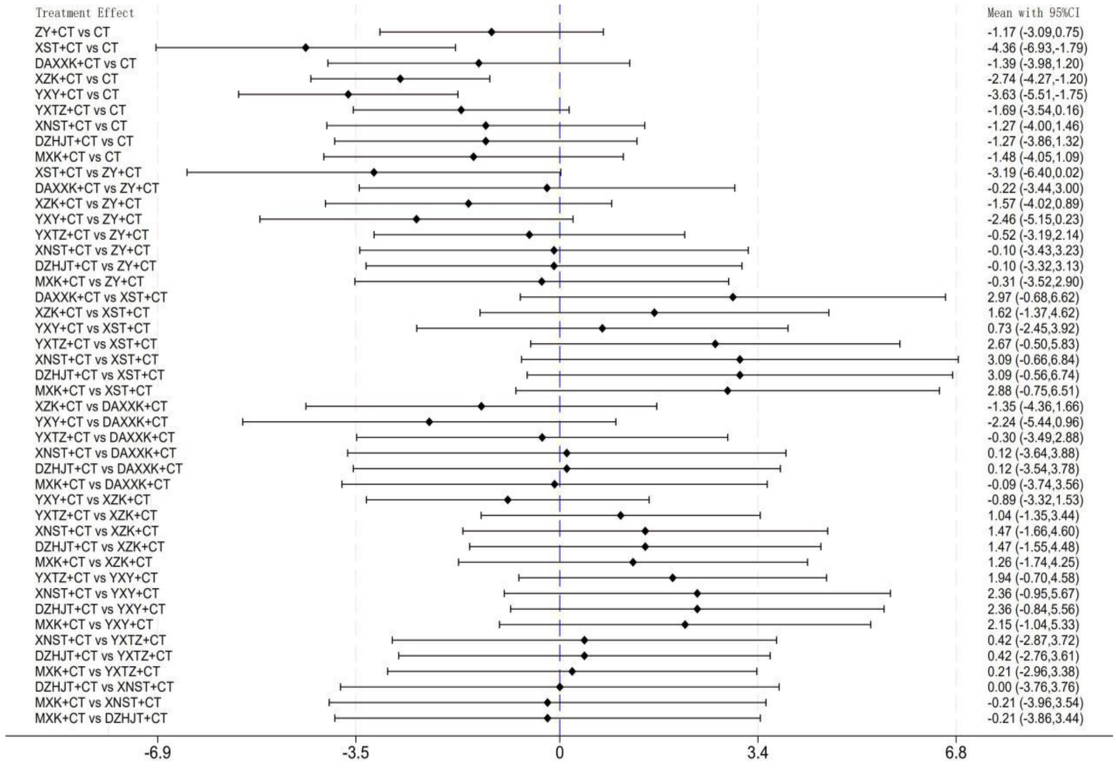

Figure S12 Plasma viscosity (PV) in a two-by-two Comparison

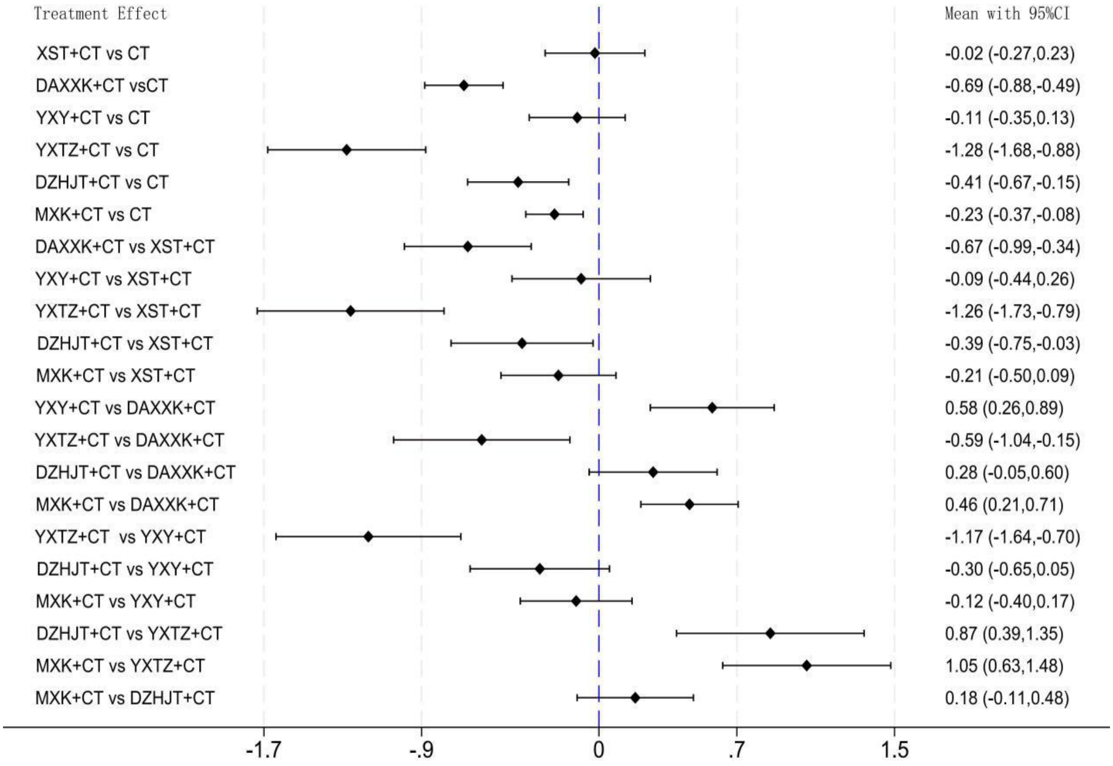

Figure S13 Major adverse cardiovascular events (MACEs) in a two-by-two Comparison

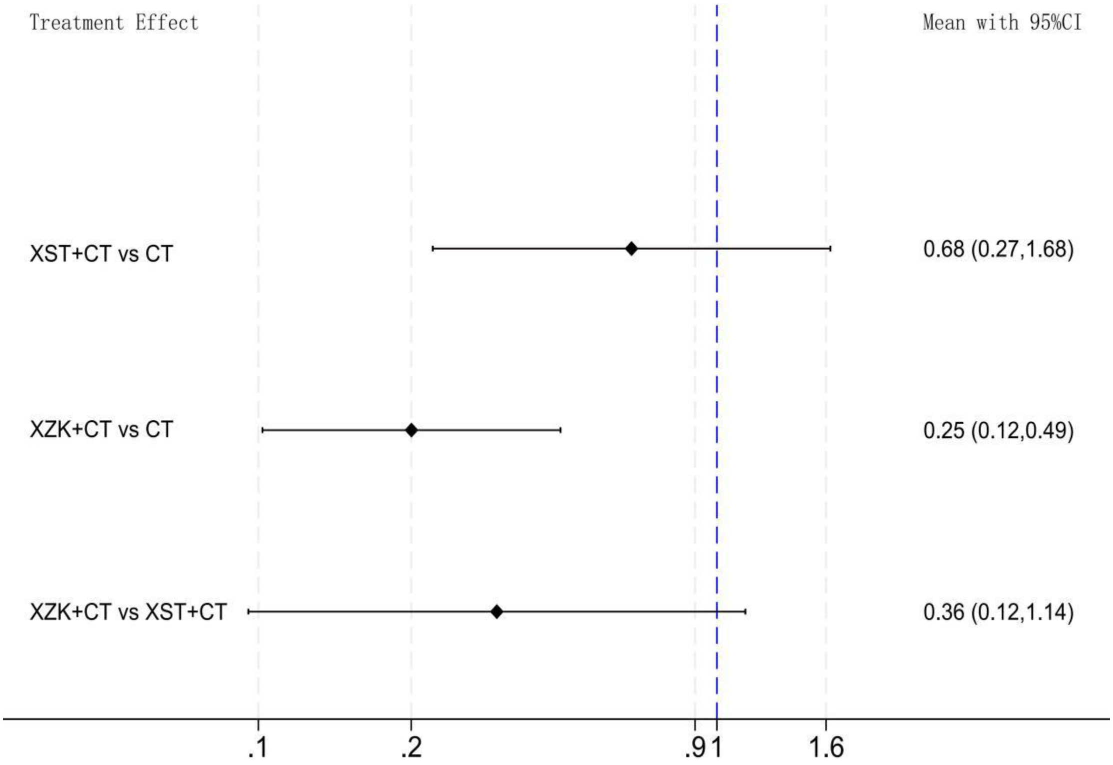

Sensitivity analysis

Figure S14 Sensitivity analysis for angina efficacy

**MXK+CT**								
**1.00 (0.84,1.19)**	**XY+CT**							
**1.03 (0.90,1.17)**	**1.03 (0.86,1.22)**	**XZK+CT**						
**0.99 (0.65,1.51)**	**0.99 (0.64,1.53)**	**0.97 (0.64,1.47)**	**XST+CT**					
**1.07 (0.94,1.23)**	**1.07 (0.90,1.28)**	**1.05 (0.91,1.20)**	**1.08 (0.71,1.64)**	**DAXXK+CT**				
**1.10 (0.95,1.28)**	**1.10 (0.92,1.33)**	**1.08 (0.93,1.25)**	**1.11 (0.73,1.70)**	**1.03 (0.89,1.19)**	**ZY+CT**			
**1.11 (0.92,1.33)**	**1.11 (0.89,1.38)**	**1.08 (0.90,1.30)**	**1.12 (0.72,1.73)**	**1.03 (0.86,1.24)**	**1.00 (0.83,1.22)**	**DZHJT+CT**		
**1.27 (1.10,1.47)***	**1.27 (1.06,1.53)***	**1.24 (1.07,1.43)***	**1.28 (0.84,1.95)**	**1.18 (1.03,1.37)***	**1.15 (0.99,1.34)**	**1.15 (0.95,1.39)**	**XNST+CT**	
**1.30 (1.18,1.44)***	**1.30 (1.12,1.51)***	**1.27 (1.16,1.40)***	**1.31 (0.87,1.97)**	**1.22 (1.10,1.34)***	**1.18 (1.06,1.32)***	**1.18 (1.01,1.38)***	**1.03 (0.92,1.14)**	**CT**

Figure S15 Sensitivity analysis for ECG efficacy

**XZK+CT**					
**1.03 (0.78,1.36)**	**ZY+CT**				
**1.08 (0.82,1.42)**	**1.04 (0.82,1.32)**	**DZHJT+CT**			
**1.08 (0.85,1.39)**	**1.05 (0.85,1.29)**	**1.01 (0.82,1.24)**	**MXK+CT**		
**1.10 (0.86,1.42)**	**1.07 (0.86,1.33)**	**1.03 (0.83,1.27)**	**1.02 (0.85,1.22)**	**DAXXK+CT**	
**1.28 (1.03,1.60)***	**1.24 (1.05,1.47)***	**1.19 (1.01,1.42)***	**1.19 (1.05,1.34)***	**1.16 (1.02,1.33)***	**CT**

Subgroup analysis

Figure S16 Subgroup analysis for angina efficacy (Treatment courses≤4 weeks)

XZK+CT										
1.04 (0.80,1.36)	MXK+CT									
1.04 (0.78,1.38)	1.00 (0.83,1.20)	XY+CT								
1.04 (0.77,1.42)	1.00 (0.81,1.24)	1.01 (0.79,1.28)	XDK+CT							
1.12 (0.86,1.45)	1.07 (0.94,1.23)	1.08 (0.91,1.28)	1.07 (0.87,1.31)	DAXXK+CT						
1.13 (0.87,1.49)	1.09 (0.93,1.27)	1.09 (0.91,1.31)	1.09 (0.87,1.35)	1.01 (0.88,1.16)	YXTZ+CT					
1.15 (0.87,1.50)	1.10 (0.94,1.28)	1.10 (0.92,1.33)	1.10 (0.88,1.36)	1.02 (0.89,1.18)	1.01 (0.86,1.18)	ZY+CT				
1.15 (0.89,1.48)	1.10 (0.98,1.24)	1.11 (0.94,1.29)	1.10 (0.91,1.33)	1.03 (0.93,1.13)	1.01 (0.90,1.14)	1.00 (0.89,1.13)	XST+CT			
1.21 (0.94,1.56)	1.16 (1.03,1.31)*	1.17 (0.99,1.37)	1.16 (0.95,1.41)	1.08 (0.98,1.20)	1.07 (0.94,1.21)	1.06 (0.93,1.20)	1.06 (0.97,1.15)	YXY+CT		
1.32 (1.01,1.72)*	1.27 (1.09,1.47)*	1.27 (1.06,1.53)*	1.26 (1.02,1.56)*	1.18 (1.03,1.35)*	1.16 (1.00,1.36)*	1.15 (0.99,1.34)	1.15 (1.02,1.30)*	1.09 (0.96,1.23)	XNST+CT	
1.35 (1.06,1.73)*	1.30 (1.17,1.44)*	1.30 (1.12,1.51)*	1.30 (1.08,1.56)*	1.21 (1.11,1.32)*	1.19 (1.07,1.33)*	1.18 (1.06,1.32)*	1.18 (1.12,1.24)*	1.12 (1.05,1.19)*	1.03 (0.92,1.14)	CT

Figure S17 Subgroup analysis for angina efficacy (Treatment courses>4 weeks)

**MXK+CT**						
**1.05 (0.73,1.53)**	**YXY+CT**					
**1.08 (0.71,1.66)**	**1.03 (0.73,1.45)**	**YXTZ+CT**				
**1.09 (0.77,1.53)**	**1.03 (0.82,1.29)**	**1.00 (0.74,1.36)**	**XZK+CT**			
**1.09 (0.72,1.64)**	**1.03 (0.75,1.43)**	**1.00 (0.69,1.47)**	**1.00 (0.75,1.33)**	**XDK+CT**		
**1.13 (0.76,1.70)**	**1.08 (0.78,1.48)**	**1.05 (0.72,1.52)**	**1.04 (0.79,1.38)**	**1.04 (0.73,1.49)**	**DZHJT+CT**	
**1.33 (0.97,1.83)**	**1.27 (1.04,1.54)***	**1.23 (0.93,1.63)**	**1.23 (1.09,1.38)***	**1.23 (0.95,1.59)**	**1.18 (0.92,1.51)**	**CT**

Figure S18 Subgroup analysis for ECG efficacy (Treatment courses≤4 weeks)

**XDK+CT**								
**1.33 (0.97,1.82)**	**YXTZ+CT**							
**1.32 (0.91,1.91)**	**0.99 (0.73,1.36)**	**XZK+CT**						
**1.50 (1.10,2.06)***	**1.13 (0.89,1.44)**	**1.14 (0.84,1.56)**	**ZY+CT**					
**1.51 (1.13,2.02)***	**1.14 (0.93,1.40)**	**1.15 (0.86,1.53)**	**1.00 (0.82,1.24)**	**XST+CT**				
**1.50 (1.00,2.23)***	**1.13 (0.80,1.59)**	**1.14 (0.76,1.69)**	**1.00 (0.71,1.40)**	**0.99 (0.72,1.37)**	**MXK+CT**			
**1.61 (1.20,2.16)***	**1.21 (0.98,1.50)**	**1.22 (0.91,1.64)**	**1.07 (0.86,1.33)**	**1.06 (0.89,1.27)**	**1.07 (0.77,1.49)**	**DAXXK+CT**		
**1.60 (1.21,2.12)***	**1.21 (0.99,1.47)**	**1.22 (0.92,1.61)**	**1.07 (0.88,1.29)**	**1.06 (0.91,1.24)**	**1.07 (0.78,1.47)**	**1.00 (0.85,1.17)**	**YXY+CT**	
**1.87 (1.44,2.44)***	**1.41 (1.19,1.67)***	**1.42 (1.09,1.85)***	**1.24 (1.05,1.47)***	**1.24 (1.10,1.40)***	**1.25 (0.93,1.69)**	**1.16 (1.02,1.33)***	**1.17 (1.06,1.29)***	**CT**

Figure S19 Subgroup analysis for ECG efficacy (Treatment courses>4 weeks)

**YXY+CT**				
**1.24 (0.90,1.70)**	**DZHJT+CT**			
**1.26 (0.93,1.70)**	**1.02 (0.82,1.26)**	**MXK+CT**		
**1.42 (0.89,2.26)**	**1.15 (0.76,1.74)**	**1.13 (0.75,1.68)**	**XZK+CT**	
**1.48 (1.13,1.93)***	**1.19 (1.01,1.42)***	**1.17 (1.03,1.34)***	**1.04 (0.71,1.52)**	**CT**

Meta-Regression Analysis

Figure S20 Details of meta-regression analysis

```
1. Empirical mean and standard deviation for each variable,
   plus standard error of the mean:

            Mean     SD  Naive SE Time-series SE
d.1.CT  0.83048 0.1976 0.0004419      0.0004924
sd.d    0.63557 0.1872 0.0004185      0.0013993
beta[1] 0.09849 0.3905 0.0008731      0.0103290

2. Quantiles for each variable:

           2.5%     25%     50%    75%  97.5%
d.1.CT   0.4386  0.7073 0.82848 0.9518 1.2292
sd.d     0.3662  0.5048 0.60379 0.7298 1.0895
beta[1] -0.7007 -0.1454 0.09937 0.3450 0.8787

-- Model fit (residual deviance):

    Dbar       pD      DIC
24.59819 23.40560 48.00380

24 data points, ratio 1.025, I^2 = 6%

-- Regression settings:

Regression on "course", unrelated coefficients, "CT" as control
Input standardized: x' = (course - 18) / 32.40651
Estimates at the centering value: course = 18
```

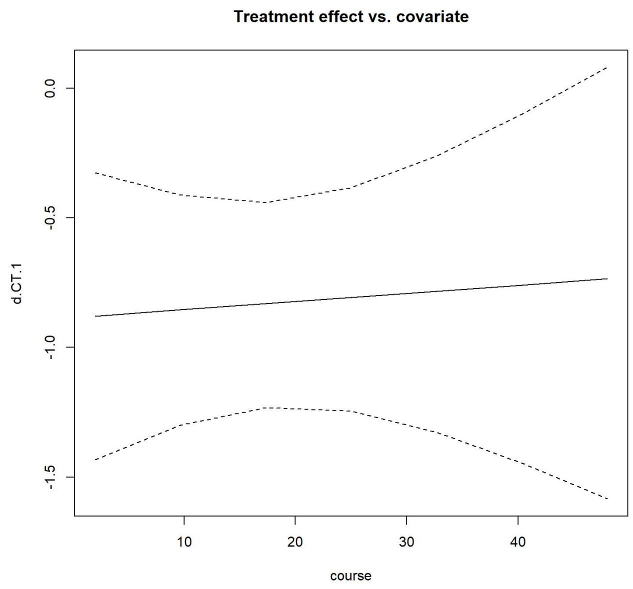

Certainty of evidence

The certainty of evidence was downgraded based on predefined criteria: 1) Risk of Bias: ① No points were deducted if all or most (two-thirds) of the data was from low-risk studies; ② -1 point if most (two-thirds) of the data was from moderate-risk bias studies; ③ -1 point if most (two-thirds) of the data was from high-risk bias studies. 2) Inconsistency (based on heterogeneity test I^2): ① No points were deducted if $I^2 \leq 50\%$; ② -1 point if $50\% < I^2 < 75\%$; ③ -2 points if $I^2 \geq 75\%$. 3) Indirectness (based on comparison type): ① No points were deducted for direct comparison between oral preparation and conventional treatment (CT); ② -1 point for indirect comparison between two oral preparations. 4) Imprecision (confidence interval crossing 1 or 0; total sample size below the optimal data size): ① -1 point if one criterion was met; ② -2 points if two criteria were met. 5) Other considerations (number of included RCTs < 30, especially with industry sponsorship; funnel plot asymmetry): -1 point if both criteria were met simultaneously.

Table S9.Certainty of evidence for angina efficacy

**Comparison**	**Risk of Bias**	**Inconsistency**	**Indirectness**	**Imprecision**	**Other considerations**	**Quality of evidence**
ZY+CT vs CT	Serious	No	No	Serious	Undetected	Low
XST+CT vs CT	Serious	No	No	No	Undetected	Moderate
XY+CT vs CT	Serious	No	No	No	Undetected	Low
DAXXK+CT vs CT	Serious	No	No	No	Undetected	Moderate
XZK+CT vs CT	Serious	No	No	No	Undetected	Moderate
YXY+CT vs CT	Serious	No	No	No	Undetected	Moderate
YXTZ+CT vs CT	Serious	No	No	No	Undetected	Moderate
XDK+CT vs CT	Serious	No	No	No	Undetected	Moderate
XNST+CT vs CT	Serious	No	No	No	Undetected	Moderate
DZHJT+CT vs CT	Serious	No	No	Serious	Undetected	Low
MXK+CT vs CT	Serious	No	No	No	Undetected	Moderate

XST+CT vs ZY+CT	Serious	No	Serious	Serious	Undetected	Very low
XY+CT vs ZY+CT	Serious	No	Serious	Serious	Undetected	Very low
DAXXK+CT vs ZY+CT	Serious	No	Serious	Very serious	Undetected	Very low
XZK+CT vs ZY+CT	Serious	No	Serious	Very serious	Undetected	Very low
YXY+CT vs ZY+CT	Serious	No	Serious	Serious	Undetected	Very low
YXTZ+CT vs ZY+CT	Serious	No	Serious	Very serious	Undetected	Very low
XDK+CT vs ZY+CT	Serious	No	Serious	Very serious	Undetected	Very low
XNST+CT vs ZY+CT	Serious	No	Serious	Very serious	Undetected	Very low
DZHJT+CT vs ZY+CT	Serious	No	Serious	Very serious	Undetected	Very low
MXK+CT vs ZY+CT	Serious	No	Serious	Very serious	Undetected	Very low
XY+CT vs XST+CT	Serious	No	Serious	Serious	Undetected	Very low
DAXXK+CT vs XST+CT	Serious	No	Serious	Serious	Undetected	Very low
XZK+CT vs XST+CT	Serious	No	Serious	Serious	Undetected	Very low
YXY+CT vs XST+CT	Serious	No	Serious	Serious	Undetected	Very low
YXTZ+CT vs XST+CT	Serious	No	Serious	Serious	Undetected	Very low
XDK+CT vs XST+CT	Serious	No	Serious	Serious	Undetected	Very low
XNST+CT vs XST+CT	Serious	No	Serious	No	Undetected	Low
DZHJT+CT vs XST+CT	Serious	No	Serious	Very serious	Undetected	Very low
MXK+CT vs XST+CT	Serious	No	Serious	Serious	Undetected	Very low
DAXXK+CT vs XY+CT	Serious	No	Serious	Serious	Undetected	Very low
XZK+CT vs XY+CT	Serious	No	Serious	Serious	Undetected	Very low
YXY+CT vs XY+CT	Serious	No	Serious	Serious	Undetected	Very low
YXTZ+CT vs XY+CT	Serious	No	Serious	Serious	Undetected	Very low
XDK+CT vs XY+CT	Serious	No	Serious	Serious	Undetected	Very low
XNST+CT vs XY+CT	Serious	No	Serious	No	Undetected	Low
DZHJT+CT vs XY+CT	Serious	No	Serious	Serious	Undetected	Very low
MXK+CT vs XY+CT	Serious	No	Serious	Serious	Undetected	Very low
XZK+CT vs DAXXK+CT	Serious	No	Serious	Serious	Undetected	Very low

YXY+CT vs DAXXK+CT	Serious	No	Serious	Serious	Undetected	Very low
YXTZ+CT vs DAXXK+CT	Serious	No	Serious	Serious	Undetected	Very low
XDK+CT vs DAXXK+CT	Serious	No	Serious	Serious	Undetected	Very low
XNST+CT vs DAXXK+CT	Serious	No	Serious	No	Undetected	Low
DZHJT+CT vs DAXXK+CT	Serious	No	Serious	Very serious	Undetected	Very low
MXK+CT vs DAXXK+CT	Serious	No	Serious	Serious	Undetected	Very low
YXY+CT vs XZK+CT	Serious	No	Serious	Serious	Undetected	Very low
YXTZ+CT vs XZK+CT	Serious	No	Serious	Serious	Undetected	Very low
XDK+CT vs XZK+CT	Serious	No	Serious	Serious	Undetected	Very low
XNST+CT vs XZK+CT	Serious	No	Serious	No	Undetected	Low
DZHJT+CT vs XZK+CT	Serious	No	Serious	Very serious	Undetected	Very low
MXK+CT vs XZK+CT	Serious	No	Serious	Serious	Undetected	Very low
YXTZ+CT vs YXY+CT	Serious	No	Serious	Serious	Undetected	Very low
XDK+CT vs YXY+CT	Serious	No	Serious	Serious	Undetected	Very low
XNST+CT vs YXY+CT	Serious	No	Serious	Serious	Undetected	Very low
DZHJT+CT vs YXY+CT	Serious	No	Serious	Very serious	Undetected	Very low
MXK+CT vs YXY+CT	Serious	No	Serious	No	Undetected	Low
XDK+CT vs YXTZ+CT	Serious	No	Serious	Serious	Undetected	Very low
XNST+CT vs YXTZ+CT	Serious	No	Serious	No	Undetected	Low
DZHJT+CT vs YXTZ+CT	Serious	No	Serious	Very serious	Undetected	Very low
MXK+CT vs YXTZ+CT	Serious	No	Serious	Serious	Undetected	Very low
XNST+CT vs XDK+CT	Serious	No	Serious	No	Undetected	Low
DZHJT+CT vs XDK+CT	Serious	No	Serious	Very serious	Undetected	Very low
MXK+CT vs XDK+CT	Serious	No	Serious	Serious	Undetected	Very low
DZHJT+CT vs XNST+CT	Serious	No	Serious	Very serious	Undetected	Very low
MXK+CT vs XNST+CT	Serious	No	Serious	No	Undetected	Low
MXK+CT vs DZHJT+CT	Serious	No	Serious	Very serious	Undetected	Very low

Table S10.Certainty of evidence for ECG efficacy

**Comparison**	**Risk of Bias**	**Inconsistency**	**Indirectness**	**Imprecision**	**Other considerations**	**Quality of evidence**
ZY+CT vs CT	Serious	No	No	No	Undetected	Moderate
XST+CT vs CT	Serious	No	No	No	Undetected	Moderate
DAXXK+CT vs CT	Serious	No	No	Serious	Undetected	Low
XZK+CT vs CT	Serious	No	No	Serious	Undetected	Low
YXY+CT vs CT	Serious	No	No	No	Undetected	Moderate
YXTZ+CT vs CT	Serious	No	No	No	Undetected	Moderate
XDK+CT vs CT	Serious	No	No	Serious	Undetected	Low
DZHJT+CT vs CT	Serious	No	No	Serious	Undetected	Low
MXK+CT vs CT	Serious	No	No	No	Undetected	Moderate
XST+CT vs ZY+CT	Serious	No	Serious	Serious	Undetected	Very low
DAXXK+CT vs ZY+CT	Serious	No	Serious	Very serious	Undetected	Very low
XZK+CT vs ZY+CT	Serious	No	Serious	Very serious	Undetected	Very low
YXY+CT vs ZY+CT	Serious	No	Serious	Serious	Undetected	Very low
YXTZ+CT vs ZY+CT	Serious	No	Serious	Serious	Undetected	Very low
XDK+CT vs ZY+CT	Serious	No	Serious	Serious	Undetected	Very low
DZHJT+CT vs ZY+CT	Serious	No	Serious	Very serious	Undetected	Very low
MXK+CT vs ZY+CT	Serious	No	Serious	Serious	Undetected	Very low
DAXXK+CT vs XST+CT	Serious	No	Serious	Very serious	Undetected	Very low
XZK+CT vs XST+CT	Serious	No	Serious	Very serious	Undetected	Very low
YXY+CT vs XST+CT	Serious	No	Serious	Serious	Undetected	Very low
YXTZ+CT vs XST+CT	Serious	No	Serious	Serious	Undetected	Very low
XDK+CT vs XST+CT	Serious	No	Serious	Serious	Undetected	Very low
DZHJT+CT vs XST+CT	Serious	No	Serious	Very serious	Undetected	Very low

MXK+CT vs XST+CT	Serious	No	Serious	Serious	Undetected	Very low
XZK+CT vs DAXXK+CT	Serious	No	Serious	Very serious	Undetected	Very low
YXY+CT vs DAXXK+CT	Serious	No	Serious	Very serious	Undetected	Very low
YXTZ+CT vs DAXXK+CT	Serious	No	Serious	Very serious	Undetected	Very low
XDK+CT vs DAXXK+CT	Serious	No	Serious	Very serious	Undetected	Very low
DZHJT+CT vs DAXXK+CT	Serious	No	Serious	Very serious	Undetected	Very low
MXK+CT vs DAXXK+CT	Serious	No	Serious	Very serious	Undetected	Very low
YXY+CT vs XZK+CT	Serious	No	Serious	Very serious	Undetected	Very low
YXTZ+CT vs XZK+CT	Serious	No	Serious	Very serious	Undetected	Very low
XDK+CT vs XZK+CT	Serious	No	Serious	Very serious	Undetected	Very low
DZHJT+CT vs XZK+CT	Serious	No	Serious	Very serious	Undetected	Very low
MXK+CT vs XZK+CT	Serious	No	Serious	Very serious	Undetected	Very low
YXTZ+CT vs YXY+CT	Serious	No	Serious	Serious	Undetected	Very low
XDK+CT vs YXY+CT	Serious	No	Serious	Serious	Undetected	Very low
DZHJT+CT vs YXY+CT	Serious	No	Serious	Very serious	Undetected	Very low
MXK+CT vs YXY+CT	Serious	No	Serious	No	Undetected	Low
XDK+CT vs YXTZ+CT	Serious	No	Serious	Very serious	Undetected	Very low
DZHJT+CT vs YXTZ+CT	Serious	No	Serious	Very serious	Undetected	Very low
MXK+CT vs YXTZ+CT	Serious	No	Serious	Serious	Undetected	Very low
DZHJT+CT vs XDK+CT	Serious	No	Serious	Very serious	Undetected	Very low
MXK+CT vs XDK+CT	Serious	No	Serious	Serious	Undetected	Very low
MXK+CT vs DZHJT+CT	Serious	No	Serious	Very serious	Undetected	Very low

Table S11.Certainty of evidence for nitroglycerin dosages

**Comparison**	**Risk of Bias**	**Inconsistency**	**Indirectness**	**Imprecision**	**Other considerations**	**Quality of evidence**

XST+CT vs CT	Serious	No	No	Serious	Suspected	Very low
XY+CT vs CT	Serious	No	No	Serious	Suspected	Very low
DAXXK+CT vs CT	Serious	No	No	Serious	Suspected	Very low
YXY+CT vs CT	Serious	No	No	Serious	Suspected	Very low
YXTZ+CT vs CT	Serious	Very serious	No	Serious	Suspected	Very low
XDK+CT vs CT	Serious	Very serious	No	No	Suspected	Very low
XNST+CT vs CT	Serious	No	No	Very serious	Suspected	Very low
DZHJT+CT vs CT	Serious	No	No	Serious	Suspected	Very low
XY+CT vs XST+CT	Serious	No	Serious	Serious	Suspected	Very low
DAXXK+CT vs XST+CT	Serious	No	Serious	Serious	Suspected	Very low
YXY+CT vs XST+CT	Serious	No	Serious	Serious	Suspected	Very low
YXTZ+CT vs XST+CT	Serious	Very serious	Serious	Serious	Suspected	Very low
XDK+CT vs XST+CT	Serious	Very serious	Serious	Serious	Suspected	Very low
XNST+CT vs XST+CT	Serious	No	Serious	Very serious	Suspected	Very low
DZHJT+CT vs XST+CT	Serious	No	Serious	Serious	Suspected	Very low
DAXXK+CT vs XY+CT	Serious	No	Serious	Serious	Suspected	Very low
YXY+CT vs XY+CT	Serious	No	Serious	Serious	Suspected	Very low
YXTZ+CT vs XY+CT	Serious	Very serious	Serious	Serious	Suspected	Very low
XDK+CT vs XY+CT	Serious	Very serious	Serious	Serious	Suspected	Very low
XNST+CT vs XY+CT	Serious	No	Serious	Very serious	Suspected	Very low
DZHJT+CT vs XY+CT	Serious	No	Serious	Serious	Suspected	Very low
YXY+CT vs DAXXK+CT	Serious	No	Serious	Serious	Suspected	Very low
YXTZ+CT vs DAXXK+CT	Serious	Very serious	Serious	Serious	Suspected	Very low
XDK+CT vs DAXXK+CT	Serious	Very serious	Serious	Serious	Suspected	Very low
XNST+CT vs DAXXK+CT	Serious	No	Serious	Very serious	Suspected	Very low
DZHJT+CT vs DAXXK+CT	Serious	No	Serious	Serious	Suspected	Very low
YXTZ vs YXY+CT	Serious	No	Serious	Serious	Suspected	Very low

XDK+CT vs YXY+CT	Serious	Very serious	Serious	Serious	Suspected	Very low
XNST+CT vs YXY+CT	Serious	No	Serious	Very serious	Suspected	Very low
DZHJT+CT vs YXY+CT	Serious	No	Serious	Serious	Suspected	Very low
XDK+CT vs YXTZ+CT	Serious	Very serious	Serious	Serious	Suspected	Very low
XNST+CT vs YXTZ+CT	Serious	Very serious	Serious	Very serious	Suspected	Very low
DZHJT+CT vs YXTZ+CT	Serious	Very serious	Serious	Serious	Suspected	Very low
XNST+CT vs XDK+CT	Serious	Very serious	Serious	Very serious	Suspected	Very low
DZHJT+CT vs XDK+CT	Serious	Very serious	Serious	Serious	Suspected	Very low
DZHJT+CT vs XNST+CT	Serious	No	Serious	Very serious	Suspected	Very low

Table S12.Certainty of evidence for frequency of angina

**Comparison**	**Risk of Bias**	**Inconsistency**	**Indirectness**	**Imprecision**	**Other considerations**	**Quality of evidence**
ZY+CT vs CT	Serious	No	No	No	Suspected	Low
XST+CT vs CT	Serious	Very serious	No	No	Suspected	Very low
XY+CT vs CT	Serious	No	No	Serious	Suspected	Very low
DAXXK+CT vs CT	Serious	Very serious	No	No	Suspected	Very low
XZK+CT vs CT	Serious	No	No	Serious	Suspected	Very low
YXY+CT vs CT	Serious	Very serious	No	Serious	Suspected	Very low
YXTZ+CT vs CT	Serious	Very serious	No	No	Suspected	Very low
XDK+CT vs CT	Serious	Very serious	No	No	Suspected	Very low
XNST+CT vs CT	Serious	No	No	Serious	Suspected	Very low
DZHJT+CT vs CT	Serious	No	No	Serious	Suspected	Very low
MXK+CT vs CT	Serious	No	No	Serious	Suspected	Very low
XST+CT vs ZY+CT	Serious	Very serious	Serious	No	Suspected	Very low
XY+CT vs ZY+CT	Serious	No	Serious	No	Suspected	Very low

DAXXK+CT vs ZY+CT	Serious	Very serious	Serious	Serious	Suspected	Very low
XZK+CT vs ZY+CT	Serious	No	Serious	No	Suspected	Very low
YXY+CT vs ZY+CT	Serious	Very serious	Serious	No	Suspected	Very low
YXTZ+CT vs ZY+CT	Serious	Very serious	Serious	Serious	Suspected	Very low
XDK+CT vs ZY+CT	Serious	Very serious	Serious	Serious	Suspected	Very low
XNST+CT vs ZY+CT	Serious	No	Serious	No	Suspected	Very low
DZHJT+CT vs ZY+CT	Serious	No	Serious	No	Suspected	Very low
MXK+CT vs ZY+CT	Serious	No	Serious	Serious	Suspected	Very low
XY+CT vs XST+CT	Serious	Very serious	Serious	Serious	Suspected	Very low
DAXXK+CT vs XST+CT	Serious	Very serious	Serious	Serious	Suspected	Very low
XZK+CT vs XST+CT	Serious	Very serious	Serious	Serious	Suspected	Very low
YXY+CT vs XST+CT	Serious	Very serious	Serious	Serious	Suspected	Very low
YXTZ+CT vs XST+CT	Serious	Very serious	Serious	Serious	Suspected	Very low
XDK+CT vs XST+CT	Serious	Very serious	Serious	Serious	Suspected	Very low
XNST+CT vs XST+CT	Serious	Very serious	Serious	Serious	Suspected	Very low
DZHJT+CT vs XST+CT	Serious	Very serious	Serious	Serious	Suspected	Very low
MXK+CT vs XST+CT	Serious	Very serious	Serious	Serious	Suspected	Very low
DAXXK+CT vs XY+CT	Serious	Very serious	Serious	Serious	Suspected	Very low
XZK+CT vs XY+CT	Serious	No	Serious	Serious	Suspected	Very low
YXY+CT vs XY+CT	Serious	Very serious	Serious	Serious	Suspected	Very low
YXTZ+CT vs XY+CT	Serious	Very serious	Serious	Serious	Suspected	Very low
XDK+CT vs XY+CT	Serious	Very serious	Serious	Serious	Suspected	Very low
XNST+CT vs XY+CT	Serious	No	Serious	Serious	Suspected	Very low
DZHJT+CT vs XY+CT	Serious	No	Serious	Serious	Suspected	Very low
MXK+CT vs XY+CT	Serious	No	Serious	Serious	Suspected	Very low
XZK+CT vs DAXXK+CT	Serious	Very serious	Serious	Serious	Suspected	Very low
YXY+CT vs DAXXK+CT	Serious	Very serious	Serious	Serious	Suspected	Very low
YXTZ+CT vs DAXXK+CT	Serious	Very serious	Serious	Serious	Suspected	Very low

XDK+CT vs DAXXK+CT	Serious	Very serious	Serious	Serious	Suspected	Very low
XNST+CT vs DAXXK+CT	Serious	Very serious	Serious	Serious	Suspected	Very low
DZHJT+CT vs DAXXK+CT	Serious	No	Serious	Serious	Suspected	Very low
MXK+CT vs DAXXK+CT	Serious	No	Serious	Serious	Suspected	Very low
YXY+CT vs XZK+CT	Serious	Very serious	Serious	Serious	Suspected	Very low
YXTZ+CT vs XZK+CT	Serious	Very serious	Serious	Serious	Suspected	Very low
XDK+CT vs XZK+CT	Serious	Very serious	Serious	Serious	Suspected	Very low
XNST+CT vs XZK+CT	Serious	No	Serious	Serious	Suspected	Very low
DZHJT+CT vs XZK+CT	Serious	No	Serious	Serious	Suspected	Very low
MXK+CT vs XZK+CT	Serious	No	Serious	Serious	Suspected	Very low
YXTZ+CT vs YXY+CT	Serious	Very serious	Serious	Serious	Suspected	Very low
XDK+CT vs YXY+CT	Serious	Very serious	Serious	Serious	Suspected	Very low
XNST+CT vs YXY+CT	Serious	Very serious	Serious	Serious	Suspected	Very low
DZHJT+CT vs YXY+CT	Serious	Very serious	Serious	Serious	Suspected	Very low
MXK+CT vs YXY+CT	Serious	Very serious	Serious	Serious	Suspected	Very low
XDK+CT vs YXTZ+CT	Serious	Very serious	Serious	Serious	Suspected	Very low
XNST+CT vs YXTZ+CT	Serious	Very serious	Serious	Serious	Suspected	Very low
DZHJT+CT vs YXTZ+CT	Serious	Very serious	Serious	Serious	Suspected	Very low
MXK+CT vs YXTZ+CT	Serious	Very serious	Serious	Serious	Suspected	Very low
XNST+CT vs XDK+CT	Serious	Very serious	Serious	Serious	Suspected	Very low
DZHJT+CT vs XDK+CT	Serious	Very serious	Serious	Serious	Suspected	Very low
MXK+CT vs XDK+CT	Serious	Very serious	Serious	Serious	Suspected	Very low
DZHJT+CT vs XNST+CT	Serious	No	Serious	Serious	Suspected	Very low
MXK+CT vs XNST+CT	Serious	No	Serious	Serious	Suspected	Very low
MXK+CT vs DZHJT+CT	Serious	No	Serious	Serious	Suspected	Very low

Table S13.Certainty of evidence for duration of angina

Comparison	Risk of Bias	Inconsistency	Indirectness	Imprecision	Other considerations	Quality of evidence
ZY+CT vs CT	Serious	No	No	No	Suspected	Low
XST+CT vs CT	Serious	Very serious	No	No	Suspected	Very low
XY+CT vs CT	Serious	Very serious	No	Serious	Suspected	Very low
DAXXK+CT vs CT	Serious	Very serious	No	No	Suspected	Very low
XZK+CT vs CT	Very serious	No	No	Serious	Suspected	Very low
YXY+CT vs CT	Serious	Very serious	No	No	Suspected	Very low
YXTZ+CT vs CT	Serious	Very serious	No	No	Suspected	Very low
XNST+CT vs CT	Serious	No	No	No	Suspected	Low
MXK+CT vs CT	Serious	No	No	No	Suspected	Low
XST+CT vs ZY+CT	Serious	Very serious	Serious	No	Suspected	Very low
XY+CT vs ZY+CT	Serious	Very serious	Serious	No	Suspected	Very low
DAXXK+CT vs ZY+CT	Serious	Very serious	Serious	No	Suspected	Very low
XZK+CT vs ZY+CT	Serious	No	Serious	No	Suspected	Very low
YXY+CT vs ZY+CT	Serious	Very serious	Serious	No	Suspected	Very low
YXTZ+CT vs ZY+CT	Serious	Very serious	Serious	No	Suspected	Very low
XNST+CT vs ZY+CT	Serious	No	Serious	Serious	Suspected	Very low
MXK+CT vs ZY+CT	Serious	No	Serious	No	Suspected	Very low
XY+CT vs XST+CT	Serious	Very serious	Serious	Serious	Suspected	Very low
DAXXK+CT vs XST+CT	Serious	Very serious	Serious	Serious	Suspected	Very low
XZK+CT vs XST+CT	Serious	Very serious	Serious	Serious	Suspected	Very low
YXY+CT vs XST+CT	Serious	Very serious	Serious	Serious	Suspected	Very low
YXTZ+CT vs XST+CT	Serious	Very serious	Serious	Serious	Suspected	Very low
XNST+CT vs XST+CT	Serious	Very serious	Serious	Serious	Suspected	Very low
MXK+CT vs XST+CT	Serious	Very serious	Serious	Serious	Suspected	Very low
DAXXK+CT vs XY+CT	Serious	Very serious	Serious	Serious	Suspected	Very low

XZK+CT vs XY+CT	Serious	Very serious	Serious	Serious	Suspected	Very low
YXY+CT vs XY+CT	Serious	Very serious	Serious	No	Suspected	Very low
YXTZ+CT vs XY+CT	Serious	Very serious	Serious	Serious	Suspected	Very low
XNST+CT vs XY+CT	Serious	Very serious	Serious	Serious	Suspected	Very low
MXK+CT vs XY+CT	Serious	Very serious	Serious	Serious	Suspected	Very low
XZK+CT vs DAXXK+CT	Serious	Very serious	Serious	Serious	Suspected	Very low
YXY+CT vs DAXXK+CT	Serious	Very serious	Serious	Serious	Suspected	Very low
YXTZ+CT vs DAXXK+CT	Serious	Very serious	Serious	Serious	Suspected	Very low
XNST+CT vs DAXXK+CT	Serious	Very serious	Serious	Serious	Suspected	Very low
MXK+CT vs DAXXK+CT	Serious	Very serious	Serious	Serious	Suspected	Very low
YXY+CT vs XZK+CT	Serious	Very serious	Serious	Serious	Suspected	Very low
YXTZ+CT vs XZK+CT	Serious	Very serious	Serious	Serious	Suspected	Very low
XNST+CT vs XZK+CT	Serious	No	Serious	Serious	Suspected	Very low
MXK+CT vs XZK+CT	Serious	No	Serious	Serious	Suspected	Very low
YXTZ+CT vs YXY+CT	Serious	Very serious	Serious	Serious	Suspected	Very low
XNST+CT vs YXY+CT	Serious	Very serious	Serious	Serious	Suspected	Very low
MXK+CT vs YXY+CT	Serious	Very serious	Serious	Serious	Suspected	Very low
XNST+CT vs YXTZ+CT	Serious	Very serious	Serious	Serious	Suspected	Very low
MXK+CT vs YXTZ+CT	Serious	Very serious	Serious	Serious	Suspected	Very low
MXK+CT vs XNST+CT	Serious	No	Serious	Serious	Suspected	Very low

Table S14.Certainty of evidence for TC

**Comparison**	**Risk of Bias**	**Inconsistency**	**Indirectness**	**Imprecision**	**Other considerations**	**Quality of evidence**
XST+CT vs CT	Very serious	Very serious	No	No	Suspected	Very low
XZK+CT vs CT	Serious	Serious	No	No	Suspected	Very low

YXTZ+CT vs CT	Serious	No	No	No	Suspected	Low
XDK+CT vs CT	Serious	No	No	Very serious	Suspected	Very low
MXK+CT vs CT	Serious	No	No	Very serious	Suspected	Very low
XZK+CT vs XST+CT	Very serious	Very serious	Very serious	Very serious	Suspected	Very low
YXTZ+CT vs XST+CT	Very serious	Very serious	Serious	Very serious	Suspected	Very low
XDK+CT vs XST+CT	Very serious	Very serious	Serious	Very serious	Suspected	Very low
MXK+CT vs XST+CT	Very serious	Very serious	Serious	Very serious	Suspected	Very low
YXTZ+CT vs XZK+CT	Serious	No	Serious	No	Suspected	Very low
XDK+CT vs XZK+CT	Serious	Very serious	Serious	Very serious	Suspected	Very low
MXK+CT vs XZK+CT	Serious	Very serious	Serious	Very serious	Suspected	Very low
XDK+CT vs YXTZ+CT	Serious	No	Serious	Very serious	Suspected	Very low
MXK+CT vs YXTZ+CT	Serious	No	Serious	Very serious	Suspected	Very low
MXK+CT vs XDK+CT	Serious	No	Serious	Very serious	Suspected	Very low

Table S15.Certainty of evidence for TG

**Comparison**	**Risk of Bias**	**Inconsistency**	**Indirectness**	**Imprecision**	**Other considerations**	**Quality of evidence**
XST+CT vs CT	Very serious	No	No	No	Undetected	Low
XZK+CT vs CT	Serious	No	No	No	Undetected	Moderate
YXTZ+CT vs CT	Serious	Serious	No	Serious	Undetected	Very low
XDK+CT vs CT	Serious	No	No	Very serious	Undetected	Very low
MXK+CT vs CT	Serious	No	No	Serious	Undetected	Low
XZK+CT vs XST+CT	Very serious	No	Serious	No	Undetected	Very low
YXTZ+CT vs XST+CT	Very serious	Very serious	Serious	Serious	Undetected	Very low
XDK+CT vs XST+CT	Very serious	No	Serious	Very serious	Undetected	Very low
MXK+CT vs XST+CT	Very serious	No	Serious	Very serious	Undetected	Very low

YXTZ+CT vs XZK+CT	Serious	Very serious	Serious	Very serious	Undetected	Very low
XDK+CT vs XZK+CT	Serious	No	Serious	Very serious	Undetected	Very low
MXK+CT vs XZK+CT	Serious	No	Serious	Very serious	Undetected	Very low
XDK+CT vs YXTZ+CT	Serious	Serious	Serious	Very serious	Undetected	Very low
MXK+CT vs YXTZ+CT	Serious	Serious	Serious	Very serious	Undetected	Very low
MXK+CT vs XDK+CT	Serious	No	Serious	Very serious	Undetected	Very low

Table S16.Certainty of evidence for LDL-C

**Comparison**	**Risk of Bias**	**Inconsistency**	**Indirectness**	**Imprecision**	**Other considerations**	**Quality of evidence**
XST+CT vs CT	Very serious	Very serious	No	No	Suspected	Very low
XZK+CT vs CT	Serious	Very serious	No	No	Suspected	Very low
YXTZ+CT vs CT	Serious	No	No	Serious	Suspected	Very low
MXK+CT vs CT	Serious	No	No	Serious	Suspected	Very low
XZK+CT vs XST+CT	Very serious	Very serious	Serious	Serious	Suspected	Very low
YXTZ+CT vs XST+CT	Very serious	Very serious	Serious	Very serious	Suspected	Very low
MXK+CT vs XST+CT	Very serious	Very serious	Serious	Very serious	Suspected	Very low
YXTZ+CT vs XZK+CT	Serious	Very serious	Serious	Very serious	Suspected	Very low
MXK+CT vs XZK+CT	Serious	Very serious	Serious	Very serious	Suspected	Very low
MXK+CT vs YXTZ+CT	Serious	No	Serious	Very serious	Suspected	Very low

Table S17.Certainty of evidence for HDL-C

**Comparison**	**Risk of Bias**	**Inconsistency**	**Indirectness**	**Imprecision**	**Other considerations**	**Quality of evidence**
XST+CT vs CT	Very serious	Very serious	No	Very serious	Undetected	Very low
XZK+CT vs CT	Serious	Very serious	No	No	Undetected	Very low

YXTZ+CT vs CT	Serious	No	No	Very serious	Undetected	Very low
XDK+CT vs CT	Serious	No	No	Very serious	Undetected	Very low
XZK+CT vs XST+CT	Very serious	Very serious	Serious	Very serious	Undetected	Very low
YXTZ+CT vs XST+CT	Very serious	Very serious	Serious	Very serious	Undetected	Very low
XDK+CT vs XST+CT	Very serious	Very serious	Serious	Very serious	Undetected	Very low
YXTZ+CT vs XZK+CT	Serious	Very serious	Serious	Very serious	Undetected	Very low
XDK+CT vs XZK+CT	Serious	Very serious	Serious	Very serious	Undetected	Very low
XDK+CT vs YXTZ+CT	Serious	No	Serious	Very serious	Undetected	Very low

Table S18.Certainty of evidence for hs-CRP

**Comparison**	**Risk of Bias**	**Inconsistency**	**Indirectness**	**Imprecision**	**Other considerations**	**Quality of evidence**
ZY+CT vs CT	Serious	No	No	Serious	Undetected	Low
XST+CT vs CT	Serious	No	No	No	Undetected	Moderate
DAXXK+CT vs CT	Serious	No	No	Very serious	Undetected	Very low
XZK+CT vs CT	Serious	Very serious	No	No	Undetected	Very low
YXY+CT vs CT	Serious	No	No	No	Undetected	Moderate
YXTZ+CT vs CT	Serious	Very serious	No	Serious	Undetected	Very low
XNST+CT vs CT	Serious	No	No	Very serious	Undetected	Very low
DZHJT+CT vs CT	Serious	No	No	Very serious	Undetected	Very low
MXK+CT vs CT	Serious	No	No	Very serious	Undetected	Very low
XST+CT vs ZY+CT	Serious	No	Serious	Very serious	Undetected	Very low
DAXXK+CT vs ZY+CT	Serious	No	Serious	Very serious	Undetected	Very low
XZK+CT vs ZY+CT	Serious	Very serious	Serious	Very serious	Undetected	Very low
YXY+CT vs ZY+CT	Serious	No	Serious	Very serious	Undetected	Very low
YXTZ+CT vs ZY+CT	Serious	Very serious	Serious	Very serious	Undetected	Very low
XNST+CT vs ZY+CT	Serious	No	Serious	Very serious	Undetected	Very low

DZHJT+CT vs ZY+CT	Serious	No	Serious	Very serious	Undetected	Very low
DAXXK+CT vs XST+CT	Serious	No	Serious	Very serious	Undetected	Very low
XZK+CT vs XST+CT	Serious	Very serious	Serious	Serious	Undetected	Very low
YXY+CT vs XST+CT	Serious	No	Serious	Serious	Undetected	Very low
YXTZ+CT vs XST+CT	Serious	Very serious	Serious	Very serious	Undetected	Very low
XNST+CT vs XST+CT	Serious	No	Serious	Very serious	Undetected	Very low
DZHJT+CT vs XST+CT	Serious	No	Serious	Very serious	Undetected	Very low
MXK+CT vs XST+CT	Serious	No	Serious	Very serious	Undetected	Very low
XZK+CT vs DAXXK+CT	Serious	Very serious	Serious	Very serious	Undetected	Very low
YXY+CT vs DAXXK+CT	Serious	No	Serious	Very serious	Undetected	Very low
YXTZ+CT vs DAXXK+CT	Serious	Very serious	Serious	Very serious	Undetected	Very low
XNST+CT vs DAXXK+CT	Serious	No	Serious	Very serious	Undetected	Very low
DZHJT+CT vs DAXXK+CT	Serious	No	Serious	Very serious	Undetected	Very low
MXK+CT vs DAXXK+CT	Serious	No	Serious	Very serious	Undetected	Very low
YXY+CT vs XZK+CT	Serious	Very serious	Serious	Serious	Undetected	Very low
YXTZ+CT vs XZK+CT	Serious	Very serious	Serious	Very serious	Undetected	Very low
XNST+CT vs XZK+CT	Serious	Very serious	Serious	Very serious	Undetected	Very low
DZHJT+CT vs XZK+CT	Serious	Very serious	Serious	Very serious	Undetected	Very low
MXK+CT vs XZK+CT	Serious	Very serious	Serious	Very serious	Undetected	Very low
YXTZ+CT vs YXY+CT	Serious	Very serious	Serious	Very serious	Undetected	Very low
XNST+CT vs YXY+CT	Serious	No	Serious	Very serious	Undetected	Very low
DZHJT+CT vs YXY+CT	Serious	No	Serious	Very serious	Undetected	Very low
MXK+CT vs YXY+CT	Serious	No	Serious	Very serious	Undetected	Very low
XNST+CT vs YXTZ+CT	Serious	Very serious	Serious	Very serious	Undetected	Very low
DZHJT+CT vs YXTZ+CT	Serious	Very serious	Serious	Very serious	Undetected	Very low
MXK+CT vs YXTZ+CT	Serious	No	Serious	Very serious	Undetected	Very low
DZHJT+CT vs XNST+CT	Serious	No	Serious	Very serious	Undetected	Very low
MXK+CT vs XNST+CT	Serious	No	Serious	Serious	Undetected	Very low

MXK+CT vs DZHJT+CT	Serious	No	Serious	Very serious	Undetected	Very low

Table S19.Certainty of evidence for PV

**Comparison**	**Risk of Bias**	**Inconsistency**	**Indirectness**	**Imprecision**	**Other considerations**	**Quality of evidence**
XST+CT vs CT	Serious	No	No	Very serious	Undetected	Very low
DAXXK+CT vsCT	Serious	Very serious	No	No	Undetected	Very low
YXY+CT vs CT	Serious	No	No	Serious	Undetected	Low
YXTZ+CT vs CT	Serious	No	No	No	Undetected	Moderate
DZHJT+CT vs CT	Serious	No	No	No	Undetected	Moderate
MXK+CT vs CT	Serious	Serious	No	No	Undetected	Low
DAXXK+CT vs XST+CT	Serious	Very serious	Serious	Serious	Undetected	Very low
YXY+CT vs XST+CT	Serious	No	Serious	Very serious	Undetected	Very low
YXTZ+CT vs XST+CT	Serious	No	Serious	Serious	Undetected	Very low
DZHJT+CT vs XST+CT	Serious	No	Serious	Serious	Undetected	Very low
MXK+CT vs XST+CT	Serious	Serious	Serious	Very serious	Undetected	Very low
YXY+CT vs DAXXK+CT	Serious	Very serious	Serious	No	Undetected	Very low
YXTZ+CT vs DAXXK+CT	Serious	Very serious	Serious	No	Undetected	Very low
DZHJT+CT vs DAXXK+CT	Serious	Very serious	Serious	Serious	Undetected	Very low
MXK+CT vs DAXXK+CT	Serious	Very serious	Serious	No	Undetected	Very low
YXTZ+CT vs YXY+CT	Serious	No	Serious	No	Undetected	Low
DZHJT+CT vs YXY+CT	Serious	No	Serious	Serious	Undetected	Very low
MXK+CT vs YXY+CT	Serious	Serious	Serious	Serious	Undetected	Very low
DZHJT+CT vs YXTZ+CT	Serious	No	Serious	No	Undetected	Low
MXK+CT vs YXTZ+CT	Serious	Serious	Serious	No	Undetected	Very low
MXK+CT vs DZHJT+CT	Serious	Serious	Serious	Serious	Undetected	Very low

Table S20.Certainty of evidence for MACEs

**Comparison**	**Risk of Bias**	**Inconsistency**	**Indirectness**	**Imprecision**	**Other considerations**	**Quality of evidence**
XST+CT vs CT	Serious	No	No	Serious	Undetected	Low
XZK+CT vs CT	Serious	No	No	No	Undetected	Moderate
XZK+CT vs XST+CT	Serious	No	Serious	Serious	Undetected	Very low
